# Genome-specific differential gene expressions in resynthesized *Brassica* allotetraploids from pair-wise crosses of three cultivated diploids revealed by RNA-seq

**DOI:** 10.3389/fpls.2015.00957

**Published:** 2015-11-04

**Authors:** Dawei Zhang, Qi Pan, Cheng Cui, Chen Tan, Xianhong Ge, Yujiao Shao, Zaiyun Li

**Affiliations:** ^1^National Key Lab of Crop Genetic Improvement, National Center of Crop Molecular Breeding Technology, National Center of Oil Crop Improvement (Wuhan), College of Plant Science and Technology, Huazhong Agricultural UniversityWuhan, China; ^2^Crop Research Institute, Sichuan Academy of Agricultural SciencesChengdu, China; ^3^College of Chemistry and Life Science, Hubei University of EducationWuhan, China

**Keywords:** *Brassica* species, allopolyploidization, differential gene expressions, transgressive gene expression, transcriptome

## Abstract

Polyploidy is popular for the speciation of angiosperms but the initial stage of allopolyploidization resulting from interspecific hybridization and genome duplication is associated with different extents of changes in genome structure and gene expressions. Herein, the transcriptomes detected by RNA-seq in resynthesized *Brassica* allotetraploids (*Brassica juncea*, AABB; *B. napus*, AACC; *B. carinata*, BBCC) from the pair-wise crosses of the same three diploids (*B. rapa*, AA; *B. nigra*, BB; *B. oleracea*, CC) were compared to reveal the patterns of gene expressions from progenitor genomes and the effects of different types of genome combinations and cytoplasm, upon the genome merger and duplication. From transcriptomic analyses for leaves and silique walls, extensive expression alterations were revealed in these resynthesized allotetraploids relative to their diploid progenitors, as well as during the transition from vegetative to reproductive development, for differential and transgressive gene expressions were variable in numbers and functions. Genes involved in glucosinolates and DNA methylation were transgressively up-regulated among most samples, suggesting that gene expression regulation was immediately established after allopolyploidization. The expression of ribosomal protein genes was also tissue-specific and showed a similar expression hierarchy of rRNA genes. The balance between the co-up and co-down regulation was observed between reciprocal *B. napus* with different types of the cytoplasm. Our results suggested that gene expression changes occurred after initial genome merger and such profound alterations might enhance the growth vigor and adaptability of *Brassica* allotetraploids.

## Introduction

Allopolyploidization which is realized through the merger and duplication of distinct parental genomes following interspecific hybridizations of two or more related species results in the origin of new allopolyploid species (Otto, 2007; Doyle et al., 2008). This pattern of speciation occurs widely in angiosperms, largely because the allopolyploids relative to their progenitors show the enhanced growth vigor (the phenomenon of heterosis) and the advantage in ecological adaptation (Chen, 2007; Leitch and Leitch, 2008). The obvious success of allopolyploidy in nature has allured the extensive investigations of genetic effects caused by genome merger at levels of the chromosomes, DNA sequences, gene expression, proteins, small RNA, and DNA methylation during the last two decades, by utilizing the continuously improved approaches of molecular biology and genome sequencing (Song et al., 1995; Soltis and Soltis, 2012; Li et al., 2014; Soltis et al., 2014). The results from recent and synthetic allopolyploids of different taxa (*Arabidopsis, Brassica*, cotton, wheat, triticale, etc.) demonstrate that the initial stage of allopolyploidization is accompanied by the various genetic, epigenetic and transcriptome changes, but the degrees of variations are obviously different between allopolyploids (Soltis and Soltis, 2012). So the new allopolyploids respond to the genome merger by rapid alterations in genomic structure and function, in order to coordinate the divergent genome at different aspects and to establish the novel plants for further evolution. As to the crucial gene expression in hybrids and new allopolyploids, widespread changes are revealed by transcriptomic analysis and extensive gene expression changes are non-additive in allopolyploids relative to their parents, including expression level dominance and transgression (outside the range of either parent), such profound changes to gene expression may enable new hybrids to survive in novel environments not accessible to their parent species (Wang et al., 2006; Chen, 2007; Rapp et al., 2009; Flagel and Wendel, 2010; Yoo et al., 2013; Li et al., 2014).

The six cultivated *Brassica* species offer a text book system of allopolyploidization through the pair-wise crosses of three diploids, which is illustrated as U-triangle (U 1935). *Brassica carinata* (2*n* = 34, BBCC), *B. juncea* (2*n* = 36, AABB), and *B. napus* (2*n* = 38, AACC) are three allotetraploids, which are derived from various two-way combinations of the three diploids *B. nigra* (2*n* = 16, BB), *B. oleracea* (2*n* = 18, CC), and *B. rapa* (2*n* = 20, AA). Resynthesized *Brassica* allotetraploids through the interspecific hybridizations between three extant *Brassica* diploid progenitors have been widely investigated to elucidate the genetic alterations during the initial stage of allopolyploid formation, since the seminal work of Song et al. (1995). The reciprocal synthetics of allotetraploids would remedy the drawback of unprecise progenitors for natural counterparts and also uniparental cytoplasm background. The results obtained provide many new insights into the genome regulations following genome merger, though the studies focused mainly on the younger *B. napus* with much more agricultural and economic importance (Gaeta et al., 2007; Nicolas et al., 2007, 2012; Xu et al., 2009; Szadkowski et al., 2010, 2011; Xiong et al., 2011; Cui et al., 2012, 2013; Ge et al., 2013). The genome of *B. rapa* has been sequenced, which should enhance the evolutionary analysis of these *Brassica* allotetraploids (Wang et al., 2011). By using the *B. rapa* as reference genome, the analysis on resynthesized *B. napus* across four generations revealed that the gene expression was more complicated than the simple combination of two genomes, and non-additive gene regulation was also detected (Jiang et al., 2013). Another transcriptomic study on synthesized *Brassica* allohexaploid and its parents showed that genome-wide changes in gene expression were involved in adaptation and evolution processes, and non-additive genes associated with important biological processes were identified (Zhao et al., 2013). An intriguing genetic interaction in three *Brassica* allopolyploids was the hierarchy of nucleolar dominance (genomes BB> AA> CC) in three allotetraploids, in which both *B. juncea* (BB> AA) and *B. carinata* (BB> CC) expressed the rRNA genes from *B. nigra*, and *B. napus* (AA> CC) expressed those from *B. rapa*, but the genes from another parent were silenced (Chen and Pikaard, 1997; Ge et al., 2013). Meanwhile, the rRNA genes silenced in vegetative tissues were expressed in reproductive tissues, indicated that the expression of rRNA genes could be tissue-specific (Chen and Pikaard, 1997). However, whether the parent-specific expression of the genes for ribosomal protein also occurs is still an open question.

In this study, the gene expressions detected by RNA-seq in three resynthesized *Brassica* allotetraploids prior to meiosis from the pair-wise crosses of the same three diploids are compared, with the aims: (1) to reveal the differential gene expressions from progenitor genomes after genome merger and duplication and with the exclusion of meiotic homoeologous exchanges; (2) to assess transgressive gene expression and its effect on adaption environment changes in *Brassica* allotetraploids; (3) to identify the expression of ribosomal protein genes after genome merger and the relationship with the nucleolar dominance. The results should give new insights into the genetic contribution of three progenitors and their interactions at the beginning of *Brassica* allopolyploidization.

## Materials and methods

### Plant materials and sample preparations

Four *Brassica* allotetraploids (AACC/CCAA, AABB, and BBCC) used in this study were previously synthesized from pair-wise crosses of three cultivated diploids, *B. rapa* L. (2*n* = 20, AA genome, genotype 3H120), *B. nigra* (L.) Koch cv. Giebra (2*n* = 16, BB)*, B. oleracea* var. *alboglabra* L. (2*n* = 18, CC, genotype Chi Jie Lan), with the aid of embryo rescue and colchicine inducing chromosome doubling *in vitro* (Cui et al., 2012, 2013). Three allotetraploids (AABB, AACC, and BBCC) were synthesized by doubling the chromosome numbers of the respective digenomic hybrids, and CCAA was directly obtained from the cultured embryo derived plantlets, probably by the spontaneous chromosome doubling *in vitro*. The plants of each synthetic were derived from single hybrid embryo by successive subculturing on MS medium, and those of each parent were derived from one seed. The rooted plantlets of four allotetraploids and three parents cultured on the medium were transferred to the pots and kept in the unheated greenhouse. Leaves at the same stage were collected from these seven materials, while the silique walls after 21 days of pollination were collected. All samples were stored in liquid nitrogen and kept at −80 until RNA extraction.

### RNA-seq library preparation and sequencing

Total RNAs were extracted by TRIzol reagent (invitrogen) and were then treated with RQ1 DNase (promega) to remove DNA. The quality and quantity of the purified RNA were determined by measuring the absorbance at 260/280 nm (A260/A280) using smartspec plus (BioRad). RNA integrity was further verified by 1.5% agrose gel electrophoresis.

For each sample, 10 μg of total RNA was used for RNA-seq library preparation. Polyadenylated mRNAs were purified and concentrated with oligo(dT)-conjugated magnetic beads (invitrogen) before used for directional RNA-seq library preparation. Purified mRNAs were iron fragmented at 95°C followed by end repair and 5′ adaptor ligation. Then reverse transcription was performed with RT primer harboring 3′ adaptor sequence and randomized hexamer. The cDNAs were purified and amplified and PCR products corresponding to 200–500 bps were purified, quantified, and stored at −80°C until used for sequencing.

For high-throughput sequencing, the libraries were prepared following the manufacturer's instructions and applied to illumina GAIIx system for 80 nt single-end sequencing by ABlife. Inc (Wuhan, China). Owing to the high cost for sequencing 2 years ago, the total of 14 (2 tissues × 7 plant samples) libraries without replicates were made for RNA-seq.

### Reads filtering and alignment

After the high-throughput sequencing, the raw data which contained adapter and low quality cycle of reads were filtered. Due to lack of reference genome from *B. nigra* and the genome sequence of *B. oleracea* was still unpublished yet 2 years ago (Liu et al., 2014), the clean reads were aligned to the *B.rapa var. pekinensis Chiifu-401* genome as described in previse study (Jiang et al., 2013; Zhao et al., 2013) using the default parameters for TopHat, allowing no more than two mismatched bases. Subsequently, only unique mapped reads were used in further study to provide sensitive and accurate alignment results, even for highly repetitive genomes. The gene expression level was calculated by using RPKM method (Reads Per kb per Million reads). If there were more than one transcript for a gene, the longest one was used to calculate its expression level and coverage.

### Analysis of differentially expressed genes (DEGs)

Program edgerR was used to analyze the differentially expressed genes between samples. Fold change ≥ 2 and *P* ≤ 0.01 between compared samples were considered as simulated biological variation in DEG analysis. GO enrichment was performed using blast2Go (http://www.blast2go.com/b2ghome). In addition, the web-based *Brassica* database (http://brassicadb.org/brad/index.php) and WEGO (Ye et al., 2006) server for gene ontology analysis was also used in this study (*P* < 0.05). We also used DAVID to investigate the transgressively expressed genes, GO terms with enrichment score ≥ 0.5 and *P* < 0.05 were considered significantly enriched (Huang et al., 2008).

Pearson correlation between biological samples was calculated using IBM SPSS V19.0 software. Additionally, the Venn diagrams in this study were performed using online tool (http://bioinfogp.cnb.csic.es/tools/venny/).

### Analysis of r-protein genes

All the mapped genes were blasted to the *Arabidopsis* genome, those genes matching the *Arabidopsis* ribosomal protein genes were considered to be r-protein genes in *Brassica*. To group r-protein genes with similar expression patterns, a hierarchical clustering was generated using the normalized expression values (log_2_ RPKM) from each library. The analysis was conducted using HemI software with Pearson correlation as the distance measure (Deng et al., 2014).

### Real-time quantitative RT-PCR (qRT-PCR) analysis

The RNA samples used for the qRT-PCR assays were the same as for the RNA-seq experiments. First-strand cDNA synthesis was performed with 1500 ng of total RNA using Thermo Scientific RevertAid First Strand cDNA Synthesis Kit, total RNA (0.5 μg) was reverse-transcribed with oligo (dT)18 primer (0.5 μg/μl) according to the described protocol. Gene-specific primers were designed according to the reference unigene sequences using the Primer 3.0, all primer sequences are listed in Supplementary Table 5. A primer was also designed for *B. napus* actin gene to normalize the amplification efficiency. QRT-PCR assays in triplicate were performed using Kapa Probe Fast qPCR Kit with a Bio-Rad CFX96 Real-Time Detection System. The actin gene was used as an internal control for data normalization, and quantitative variation in the different replicates was calculated using the delta-delta threshold cycle relative quantification method.

### Availability of supporting data

The datasets supporting the results of this article are available in GenBank SRA under accession ID PRJNA281555. Other supporting data are included within the article and its additional files.

## Results

### Gene expressions between nascent allotetraploids and parents

**Figure 1:**
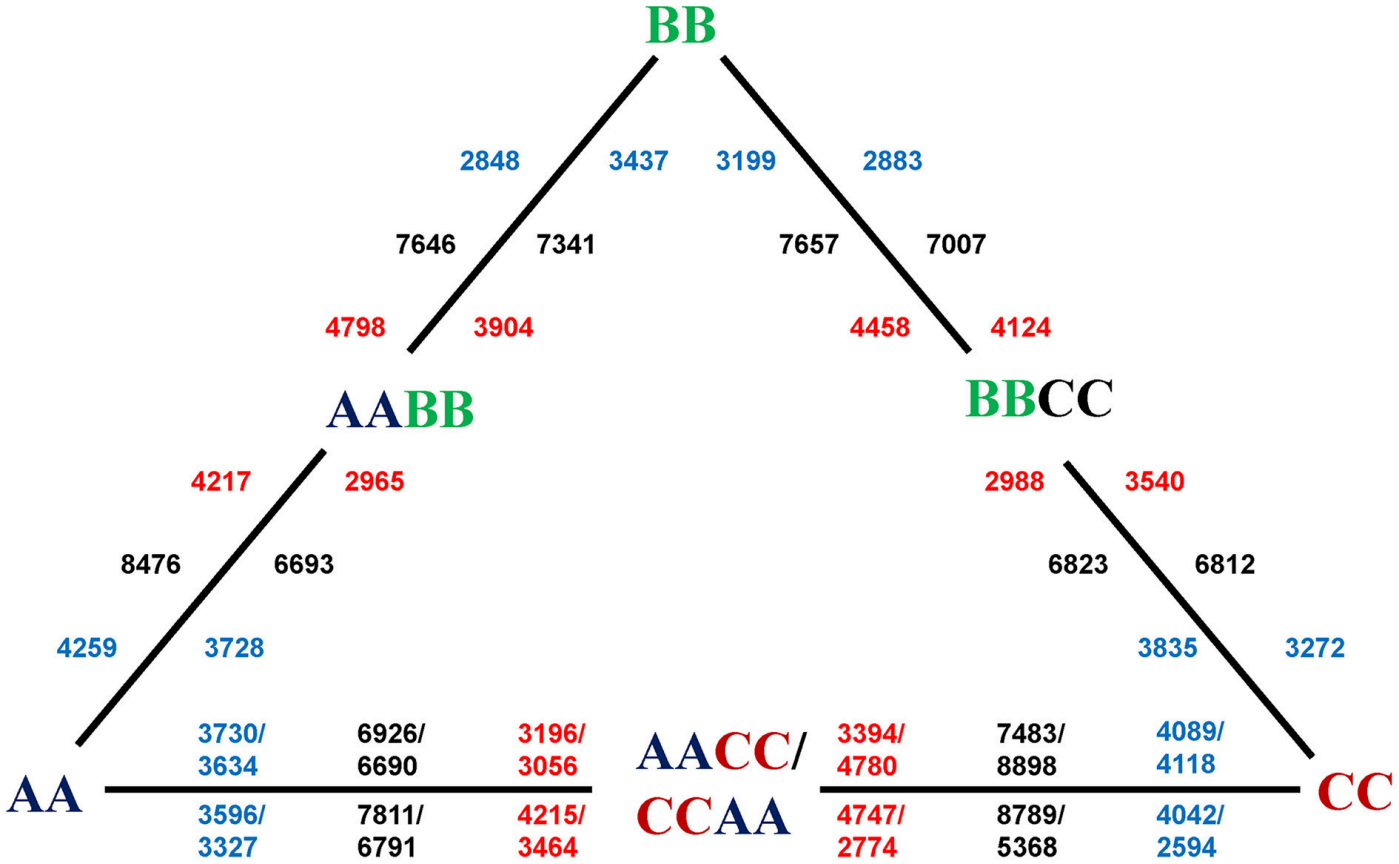
**DEGs between synthetic allotetraploids and their diploid parents**. The numbers in the inner circle of the triangle are DEGs in leaves, and those on the periphery are DEGs in silique walls. The total number (black) of DEGs between each allotetraploid and one of parent is in the middle, the number of up-regulation (red) is close to the allotetraploid, and the number of down-regulation (blue) is near to the parent. The lower number of DEGs for the silique sample of CCAA might be caused by the fewer raw reads for the sequencing problems.

To investigate and compare the mRNA expression levels in the resynthesized *Brassica* allotetraploids relative to their diploid progenitors (Figure 1), 14 RNA-seq libraries were constructed for two types of tissue: leaves (L. for short) and silique walls (S.), respectively. As a result, we obtained an average of 12,748,770 (84.02%) high-quality and clean reads from the raw reads (Supplementary Table 1). Among the clean reads, the average 41.76% reads were matched either to unique or multiple genomic positions using the *B. rapa* reference genome. As most of uniquely mapped reads were mature mRNA or ncRNA and multiple mapped reads were mainly rRNA and tRNA, only uniquely and perfectly mapped reads (3,696,642) were used to measure the transcriptional activity of each gene. Finally, our RNA-Seq data revealed an average of 28944 genes in *Brassica* allotetraploids and its progenitors, accounting for 70.56% of the total genes in *B. rapa* reference genome. For further comparative analysis, the gene expression level was calculated using RPKM method (Reads Per kb per Million reads).

**Figure 2:**
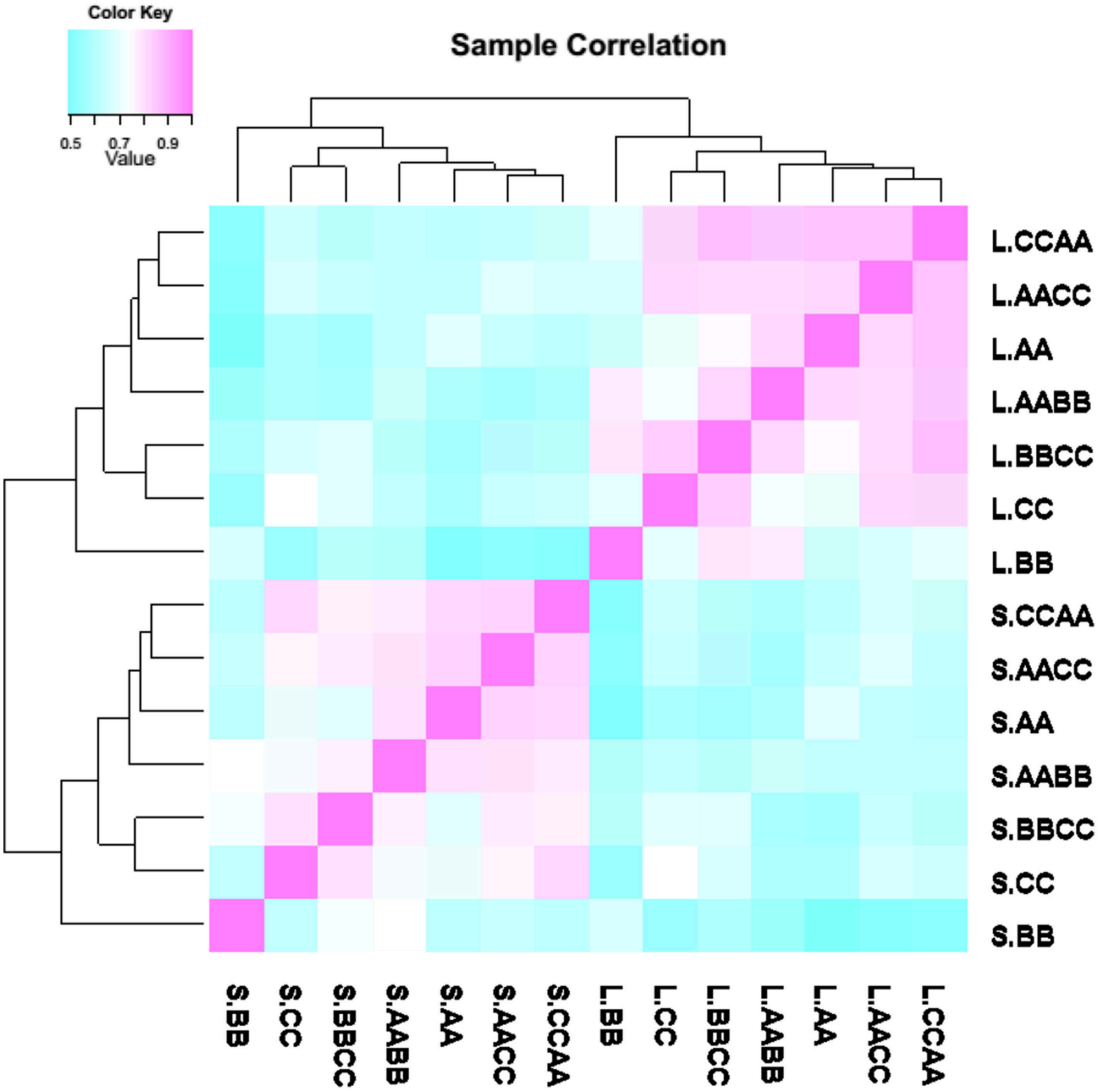
**Gene expression correlations between nascent allotetraploids and parents**. The branch length represents the extent of gene expression variance among samples.

A correlation dendrogram showed that the gene expression patterns were distinct between leaves and silique walls, for the samples of the two tissues were clustered separately (Figure 2). However, the global relationships of gene expression among the synthetics and their parents were the same in two tissues. In the dendrogram, AACC and CCAA which were most closely correlated were clustered firstly with AA and then with AABB, and BBCC was clustered with CC, while BB appeared as the outlier.

### Differentially expressed genes (DEGs) in nascent allotetraploids

To study the gene expression patterns during allopolyploidization process, we first performed pair-wise comparisons between allotetraploids to their parents to identify differentially expressed genes (DEGs) using edgerR (fold change ≥ 2 and *P* ≤ 0.01 as criteria; Supplementary Figure 1). As a result, average 3791 (13.1% of expressed genes) DEGs were up-regulated and 3534(12.2% of expressed genes) DEGs were down-regulated in allotetraploids, respectively (Figure 1). There was no significant difference between the average number of up and down-regulated genes in leaves (3593 vs. 3721; *t*-test, *P* > 0.05), however, the difference was statistically significant (3990 vs. 3347; *t*-test, *P* < 0.05) in silique walls, suggesting that the direction of DEGs was affected by tissue type. The maximum number (8898) of DEGs was observed between L.CCAA and L.CC among all comparisons, including 4780 up-regulated and 4118 down-regulated. But the minimum number (5368) of DEGs was between S.CCAA and S.CC, only 2774 genes were up-regulated and 2594 were down-regulated.

Notably, by comparing the total number of genes showing differential expression (both up and down regulation) between the allotetraploids and their parents, there was a bias in the direction of differential expression relative to the parents (Table 1). For example, more expressed genes remained statistically unchanged (less differential expression) between L.AACC/CCAA and L.AA than between L.AACC/CCAA and L.CC (Chi square test, *P* < 0.01). This asymmetric gene expression was also observed in L.AABB and L.BBCC, the global expression patterns were closer to either L.AA in L.AABB or L.CC in L.BBCC (Chi square test, *P* < 0.01). Whereas, the expression bias in silique walls was not as tangible as in leaves, the global expression patterns were closer to either S.BB in S.AABB or S.CC in L.AACC.

**Table 1:** **Summary of differential expression between the *Brassica* allotetraploids and their parents**.

	**Maternal parent^a^**	**Paternal parent**	**Less differential expression genome**
L.AABB	6693	7341	A^*^
L.BBCC	7657	6823	C^*^
L.CCAA	8898	6687	A^*^
L.AACC	6926	7483	A^*^
S.AABB	8476	7646	B^*^
S.BBCC	7007	6812	C
S.CCAA	5368	6791	C^*^
S.AACC	7098	8019	A^*^

a *The total number of genes showing differential expression between the allotetraploids and their maternal parent, including both up and down-regulated genes*.

* *Chi square test with expected ration of 1:1, P < 0.01*.

### Identification of transgressively expressed genes

Among the differentially expressed genes, we then filtered the transgressively expressed genes in allotetraploids which showed more than two-fold changes in expression relative to both parents (Table 2). Briefly, on average 878 genes (3.0% of total expressed genes) were transgressively up-regulated while 652 genes (2.3% of total expressed genes) were transgressively down-regulated. When we compared the number of genes for up and down-regulation in each sample, no significant bias was observed in leaves, but there were more genes exhibiting transgressive upregulation in silique walls.

**Table 2:** **Transgressive expressions in *Brassica* allotetraploids**.

	**Up-regulation (%)^a^**	**Down-regulation (%)**	**Biased regulation**
L.AABB	566(2.0)	489(1.8)	Up
L.BBCC	828(2.9)	841(2.9)	Down
L.CCAA	676(2.3)	692(2.3)	Down
L.AACC	649(2.3)	957(3.3)	Down^*^
S.AABB	1172(4.0)	467(1.6)	Up^*^
S.BBCC	1021(3.4)	391(1.3)	Up^*^
S.CCAA	647(2.3)	482(1.7)	Up^*^
S.AACC	1469(4.8)	896(2.9)	Up^*^
Average	878(3.0)	652(2.3)	Up^*^

a *Calculated by dividing the number of expressed genes in each Brassica allotetraploid*.

* *Chi square test with expected ration of 1:1, P < 0.01*.

**Figure 3:**
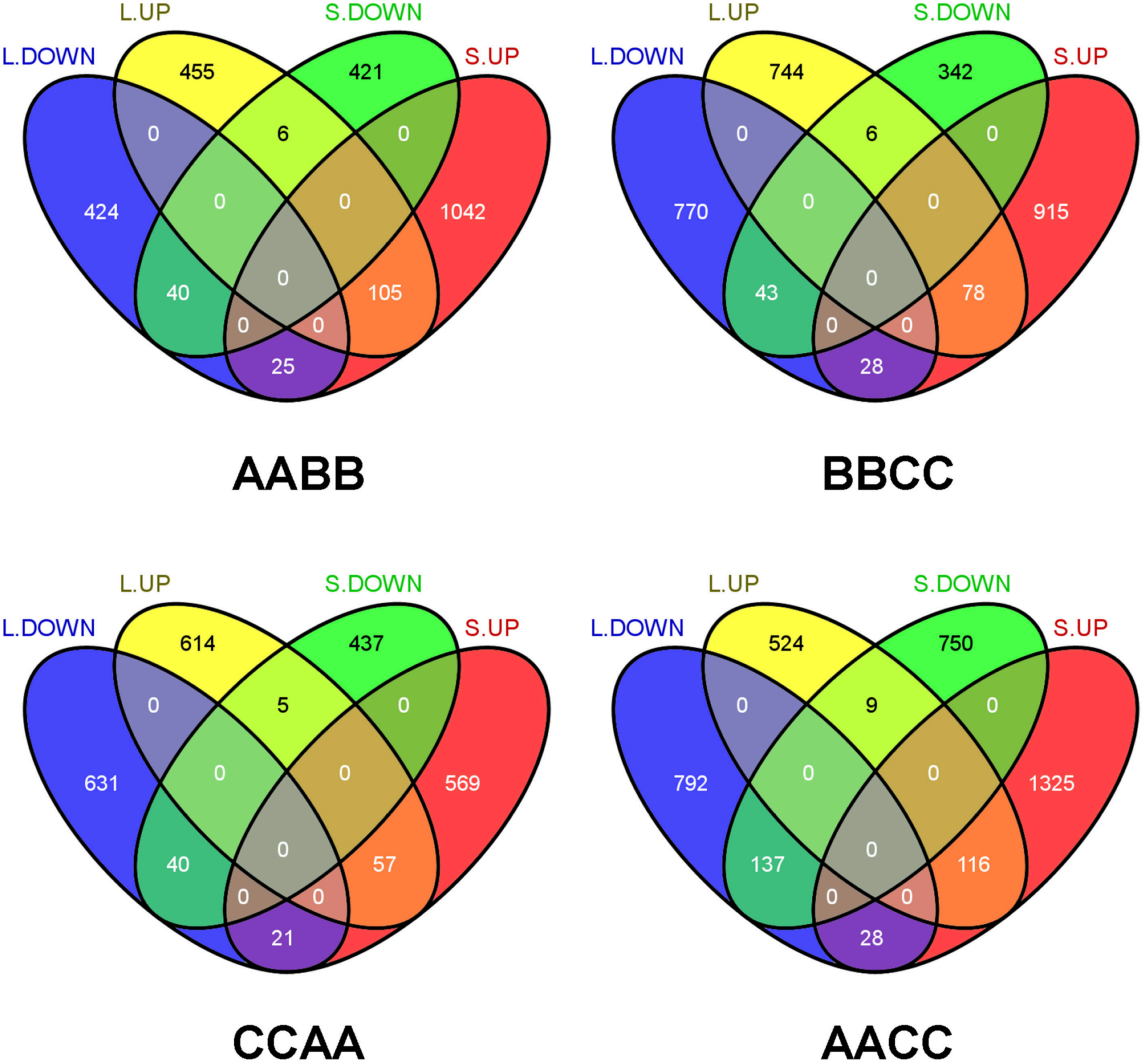
**Comparison of transgressive expression between leaves and silique walls in each *Brassica* allotetraploid**. L.UP and L.DOWN: up- and down-regulation in leaves. S.UP and S.DOWN: up-/down regulation in silique walls.

Moreover, the comparisons of transgressively expressed genes in each *Brassica* allotetraploids revealed that transgressive expression varied between tissues (Figure 3). For instance, more genes showed transgressive up-regulation in silique walls than leaves in AABB (1172 vs. 566). Among those genes, a multitude of them were specifically expressed in different direction and tissues, suggesting that the majority of transgressively expressed genes were tissue-specific. In addition, certain number of genes showed co-transgressive up- or down-regulation (105 and 40) regardless of tissue type, likely due to genome merger. However, 28 genes showing transgressive down-regulation in leaves displayed up-regulation in silique walls, while only nine genes showed expression changes in the opposite direction (From up in leaves to down in silique walls; Figure 3). The similar tendency of gene expression changes was observed in other *Brassica* allotetraploids (28 vs. 6 in BBCC; 21 vs. 5 in CCAA; 28 vs. 9 in AACC), suggesting that genes in silique walls tended to be transgressively up-regulated.

Transgressively expressed genes were further functionally classified according to Gene Ontology (GO) terms using DAVID (Huang et al., 2008). Ten functional clusters with the highest enrichment score were selected with criteria that enrichment score > 0.5 and *P* < 0.05(see more details in Supplementary Table 2). Although remarkable difference was observed among samples, it was interesting to note that a certain functional cluster encoding glucosinolates was up-regulated among a group of allotetraploids (L.BBCC, L.CCAA, L.AACC, S.AABB, S.AACC), suggesting that the increased expression of resistance-related genes, especially glucosinolates, was common in *Brassica* allotetraploids (Supplementary Table 2).

### Co-transgressive gene expression in *Brassica* allotetraploids

To further ascertain which genes were specially expressed in tissues regardless of genome composition and what the function of those genes was, we investigated genes showing co-transgressive expression in leaves and silique walls, respectively. To avoid confusion, three representative allotetraploids (AABB, BBCC, and CCAA) with different cytoplasm were selected for further comparative analysis.

**Figure 4:**
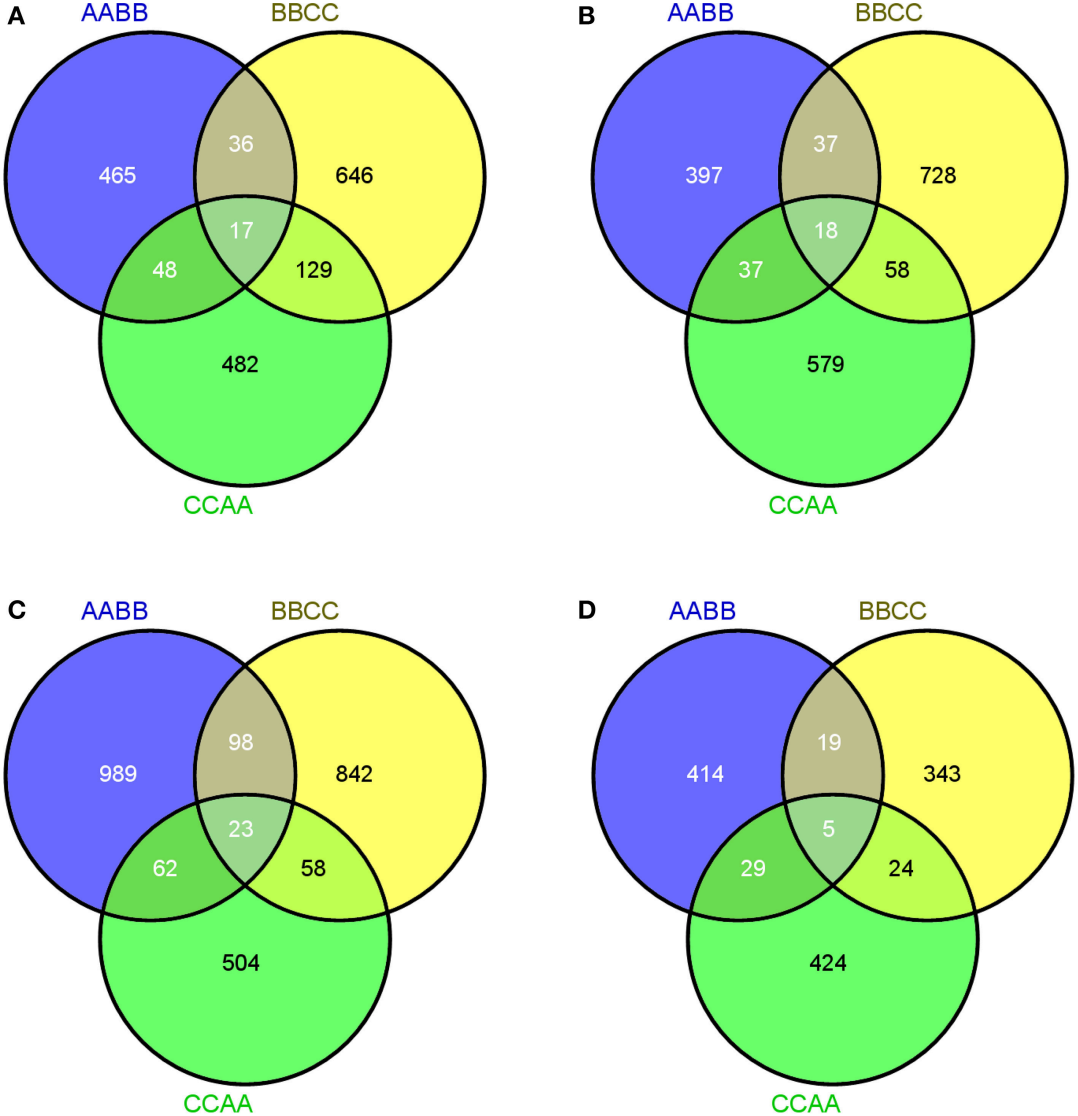
**Co-transgressive gene expressions of *Brassica* allotetraploids**. **(A,B)** Up- and down-regulated genes in leaves. **(C,D)** Up- and down-regulated genes in silique walls.

As illustrated in the Venn diagrams, 17 genes exhibited co-transgressive up-regulation and 18 genes gave co-transgressive down-regulation among the allotetraploids in leaves, respectively (Figures 4A,B). Interestingly, two key methylase genes (Bra002610 and Bra022537) which were involved in DNA methylation on cytosine and methyltransferase activity reflected co-transgressive up-regulation in the allotetraploids, suggesting that methylation-related genes were up-regulated during *Brassica* allopolyploidization in leaves (Supplementary Table 4). In silique walls, there were more genes displaying co-transgressive up-regulation than down-regulation (23 vs. 5, Figures 4C,D).

### Novel gene expression and silencing in allotetraploids

To explore novel gene expression and gene silencing in the allotetraploids, a strict criterion was set: novel expression was defined when both parental lines had no reads but in allotetraploids and the genes displayed more than 10 reads and RPKM ≥ 2. On the contrary, if both parental lines contained genes with more than 10 reads and RPKM ≥ 2, yet allotetraploids had no reads, we considered this situation as gene silencing.

**Figure 5:**
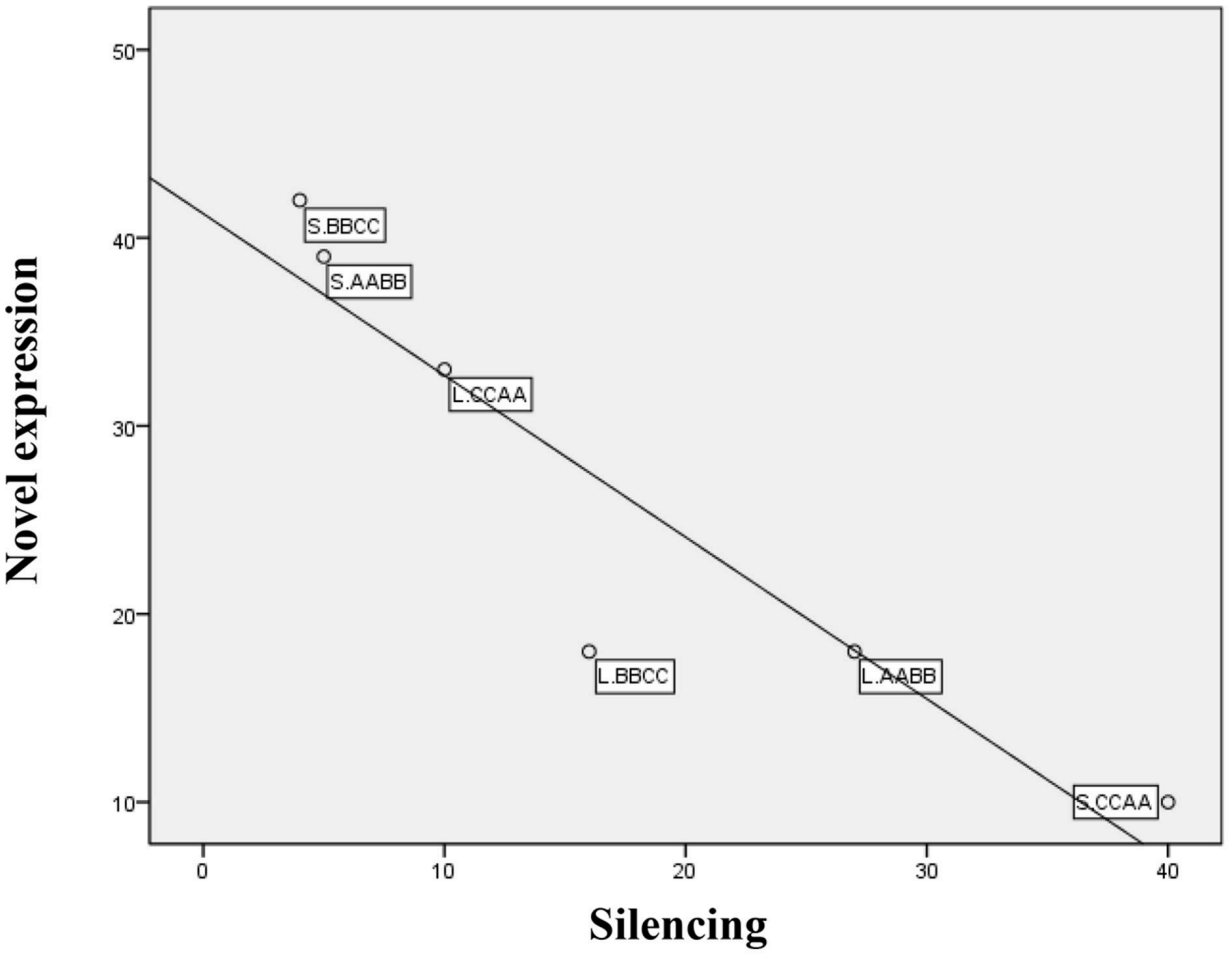
**Inverse correlations between number of genes for novel expression and silencing among samples**.

Overall, a total of 160 genes were found to be novel expression, while 102 genes exhibited silencing in allotetraploids, suggesting that these changes were tightly controlled (Table 3). There was no significant difference between the total number of genes showing novel expression and silencing in both tissues, but considerable variation between the two different expression patterns was found in silique walls: more genes exhibited silencing than novel expression in S.CCAA, whereas more genes showed novel expression than silencing in S.BBCC and S.AABB. Furthermore, the number of genes showing novel expression in each sample was negatively correlated with those genes being silenced (Pearson correlation, *r* = −0.92, *P* = 0.008 < 0.01; Figure 5).

**Table 3:** **Genes showing novel expression and silencing in three *Brassica* allotetraploids**.

**Samples**	**Novel expression(%)^a^**	**Silencing(%)^b^**
L.AABB	18(0.06)	27(0.10)
L.BBCC	18(0.06)	16(0.06)
L.CCAA	33(0.12)	10(0.04)
S.AABB	39(0.13)	5(0.01)
S.BBCC	42(0.14)	4(0.01)
S.CCAA	10(0.04)	40(0.14)
Total	160	102

a, b *Calculated by dividing the number of expressed genes in each Brassica allotetraploid*.

The biological functions and processes of the two expression patterns were analyzed using gene ontology annotations according to function annotation convention (Supplementary Figure 2). The enriched GO terms were similar between tissues, while the number of genes was various in the enriched GO terms. The genes associated with cell part, cellular, as well as metabolic process and response to stimulus were over-represented in both tissues. However, there were more genes showing novel expression than silencing in those GO terms in leaves, but more genes exhibited silencing than novel expression in silique walls.

### Differentially expressed genes between reciprocal *B. napus*

To evaluate how gene expression was affected by cytoplasm types, we examined gene expression changes between the reciprocal synthetics of *B. napus* (AACC/CCAA) which kept the same nuclear genomes but the cytoplasm from *B. rapa* or *B. oleracea*. Though a large proportion of genes showed differential expression, the total number of up-regulated genes was similar to that of down-regulated genes (6210 vs. 6270), suggesting that there was no bias in the direction of differential expression between AACC and CCAA (Chi square text,χ^2^ = 0.29, *P* > 0.01; Table 4). Among these differentially expressed genes, only those showing co-regulation were selected for subsequent function analysis to avoid the tissues specific effects, the difference between co-up (581) and co-down (530) regulation was also insignificant (Chi square text, χ^2^ = 2.25, *P* > 0.01; Table 4).

**Table 4:** **Genes showing co-regulation in leaves and silique walls between AACC and CCAA**.

	**Up-regulation**	**Down-regulation**	**Biased regulation**
Leaves	2633	3285	Down^*^
Silique walls	3577	2985	Up^*^
Total	6210	6270	Up
Co-regulation	581(9.36%)	530(8.45%)	Up

* *Chi square test with expected ration of 1:1, P < 0.01*.

**Figure 6:**
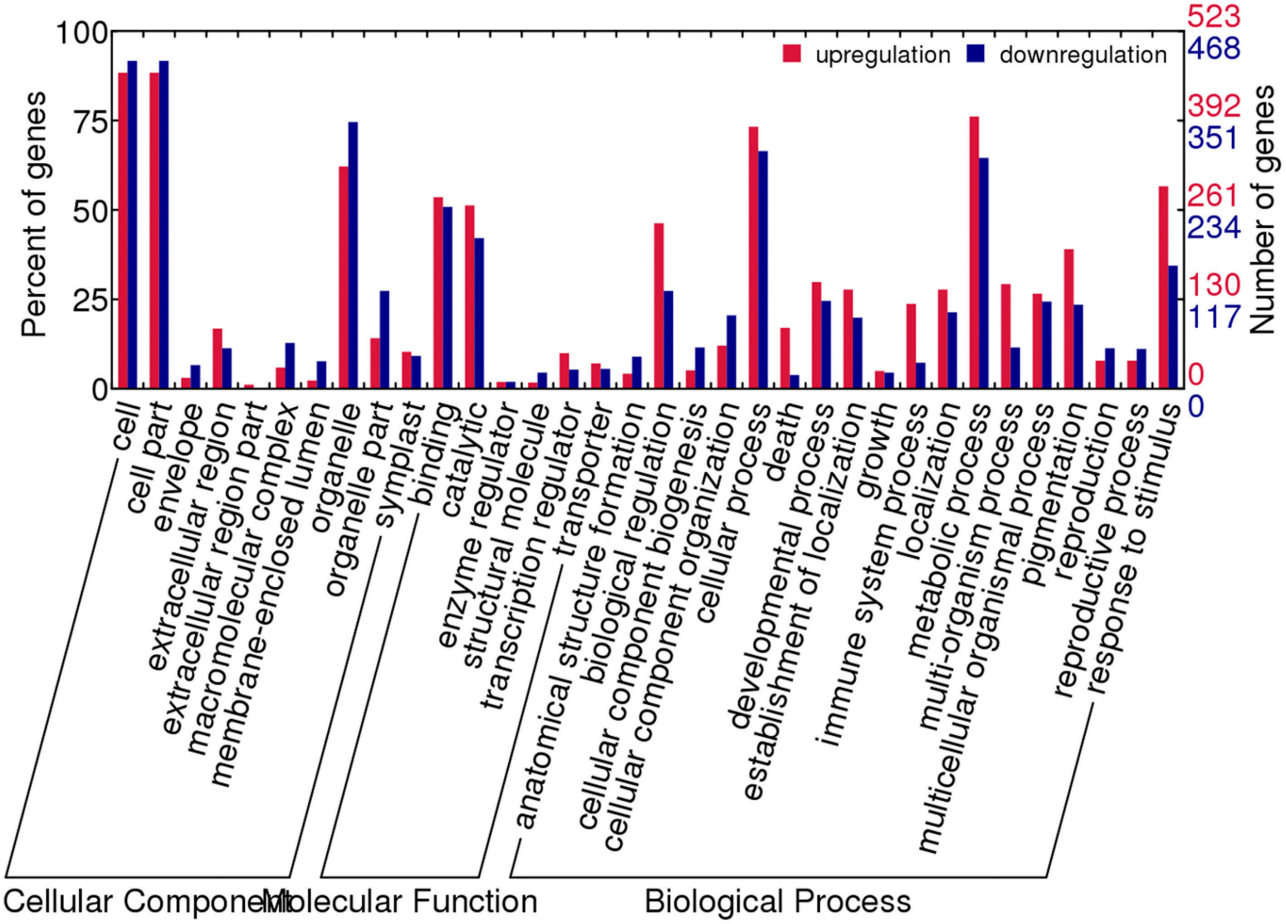
**GO functional categories of genes showing co-differential expression between AACC and CCAA**.

To have an overview of the major differences between the reciprocal synthetics, we performed GO analysis of the differential expressed genes. We found that the number of co-up regulated genes was similar to that of co-down regulated in the most enriched classes (Figure 6). Among the cellular component categories, cell and cell parts were the largest enrichment, followed by organelle. As to the molecular function categories, the most over-represented GO terms were binding and catalytic, far more than others. In the biological process categories, there were more GO terms compared to the other two categories, and slightly more genes involved in the biological regulation, cellular process, metabolic process as well as response to stimulus were found to be up-regulated in these GO terms.

### Analysis of r-protein gene expression

Besides the afore-mentioned expression patterns, we further identified ribosomal protein genes among samples to evaluate whether the genome merger has similar effect on housekeeping genes. We compared the *Arabidopsis* r-proteins against the above mapped genes based on the sequencing data. 363 genes were identified in our libraries which matched a total of 79 of the 80 r-proteins identified in *Arabidopsis*, suggesting that there was a high number of homologous r-proteins genes between *Brassica* and *Arabidopsis* (Supplementary Table 4; Whittle and Krochko, 2009). However, the expression of r-protein genes varied extensively across 14 libraries, the average expression level of r-protein genes in leaves was generally higher than that in silique walls (Supplementary Figure 3). The highest expression level of r-proteins genes were detected in L.AABB and L.BBCC, however, it was notable that the lowest expression level were also found in their silique walls (S.AABB and S.BBCC).

**Figure 7:**
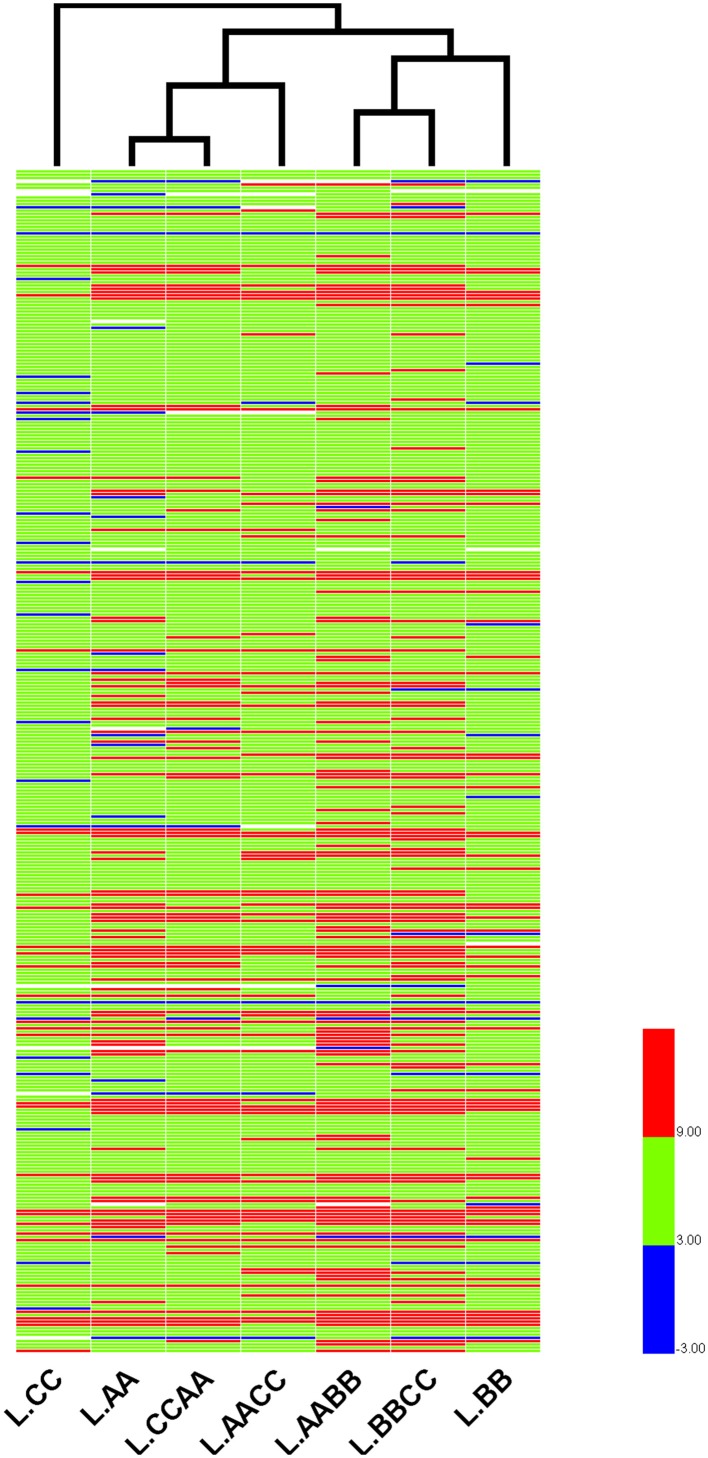
**Hierarchical clustering of r-protein genes among allotetraploids and diploids in leaves**.

**Figure 8:**
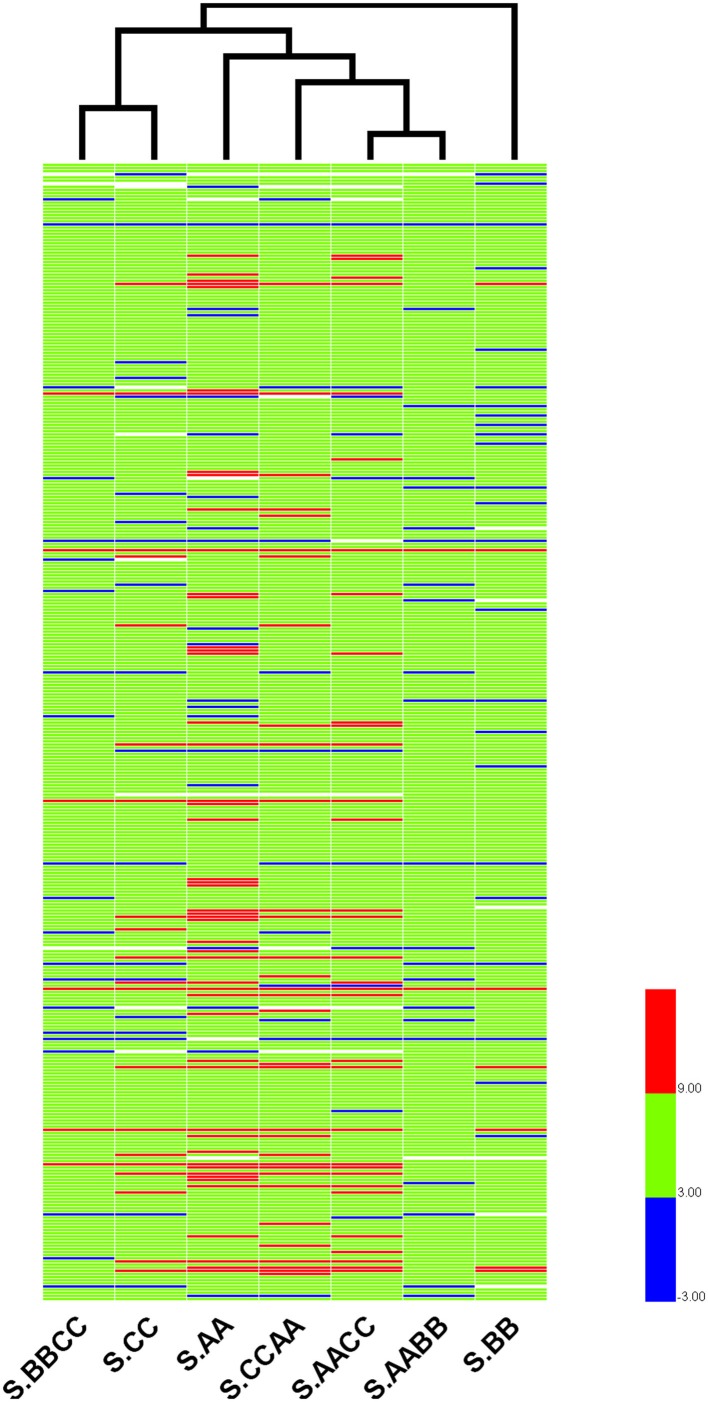
**Hierarchical clustering of r-protein genes among allotetraploids and diploids in silique walls**.

To further reveal the relationships of r-proteins gene expression among *Brassica* allotetraploids and diploids in different tissue, hierarchical clustering of expression level (standardized by log_2_ RPKM) was conducted for the 363 expressed r-proteins genes (encoding 79 r-proteins) using HemI (Deng et al., 2014). Those *Brassica* allotetraploids and diploids with similar expression profiles of r-proteins genes were more closely clustered in leaves and silique walls, respectively (Figures 7, 8). In leaves, two allotetraploids with B-genome (L.AABB and L.BBCC) were closer to each other and clustered with L.BB, while reciprocal *B. napus* (L.AACC/L.CCAA) exhibited similar transcription profiles with L.AA and diverged markedly from L.CC. In silique walls, r-protein genes retained similar expression pattern to that in leaves on whole genome scale. Three allotetraploids with A-genome (S.AACC, S.CCAA, and S.AABB) were closer to S.AA and separated from S.CC and S.BBCC, while all of them were quite distinct from S.BB.

### Verification of DEGs by qRT-PCR

To confirm that the above results of differentially expressed genes, a set of gene-specific primers were designed for quantitative RT-PCR assays (Supplementary Table 5). The relative transcript levels in the allotetraploids and their parents were compared with those of RNA-seq data (RPKM value). For 24 out of the 30 comparisons, qRT-PCR analysis revealed the same expression trends as the RNA-seq data, despite some quantitative differences (Supplementary Figure 4). Among 25 genes showing transgressive expression in the allotetraploids relative to their parents using RNA-Seq, 12 were up-regulated, eight were down-regulated, and five were differentially expressed to only one of their parents, respectively. Moreover, four of five genes showing differential expression between the reciprocal synthetics of *B. napus* also showed the same expression trends revealed by qRT-PCR and RNA-seq, confirming the reliability of RNA-seq data.

## Discussion

Widespread changes of gene expression during allopolyploidization have been revealed in different aspects, including homoeolog expression bias, novel gene expression, or silencing, transgressive up or down-regulation, expression level dominance, and altered expression times and locations (Doyle et al., 2008). In this study, global transcriptome analyses through RNA-Seq were made for serial synthetic *Brassica* allotetraploids derived from the same three diploid parents (Figure 1; Cui et al., 2012). We focused on general analysis of transgressive gene expression, novel gene expression, and gene silencing, r-protein genes expression upon *Brassica* allopolyploidization. In addition, qRT-PCR analysis was also performed to confirm the reliability of RNA-seq data.

### Effects of transgressive gene expression during allopolyploidization

In consistence with other findings of gene expressions in cotton (Yoo et al., 2013), wheat (Li et al., 2014), and *Senecio* (Hegarty et al., 2008), transgressive gene expression in our *Brassica* allotetraploids was genome-wide and temporal. A lot of genes exhibited opposite expression direction between tissues, suggesting that transgressively expressed genes had different roles in development (Table 2). Additionally, transgressively up-regulated gene expression increased over time, more genes were or reversed to be transgressively up-regulated in silique walls (Figure 3). Moreover, wide ranges of alterations occurred for the functional clusters of transgressively expressed genes between tissues (Supplementary Table 2). All these changes indicated that transgressive gene expression was sophisticated in different genome background and different growth phases. In particular, transgressive up-regulation of resistance-related genes including for the synthesis of glucosinolates could be responsible for immediate physiological pre-adaptation of *Brassica* allotetraploids, since glucosinolates and their breakdown products played a role in the defense of plants against pathogen (Brader et al., 2001), fungi (Hiruma et al., 2010), and insects (Ahuja et al., 2015).

Interestingly, the genes which involved in DNA methylation were transgressively up-regulated in leaves across three types of *Brassica* allotetraploid (Figure 4A; Supplementary Table 3). DNA methylation was an important and best-studied epigenetic phenomenon in allopolyploids. Allopolyploids (e.g., wheat, *Senecio, Spartina*) underwent rapid and widespread metylation state changes and significant proportion of these changes showed non-additivity in previous studies (Dong et al., 2005; Salmon et al., 2005; Hegarty et al., 2011). Recent investigations in synthesized *Brassica* hybrids and allotetraploids also provided evidence for rapid changes in DNA methylation by transcriptome analysis or genetic analysis (Xu et al., 2012; Cui et al., 2013; Jiang et al., 2013; Zhao et al., 2013). Consistent with these studies, our data further demonstrated that the expression of methylation-related genes was non-additive and indeed transgressively up-regulated. DNA methylation was vitally important in silencing transposons and regulating gene expression, typically reducing expression (Zilberman et al., 2007; Zhang, 2008), as a consequence, the reduced expression and transposon silencing could prove capacity for adaptation.

Together, these data suggested that transgressive gene expression could be various in number and function between tissues. Such variation (including DNA methylation) might play a causative role in regulation of gene expressions in allopolyploids and increase their relative fitness over their parents in novel environment.

### Negative correlation between novel gene expression and silencing

Evidence for rapid gene silencing and novel expression came from the detection of missing parental fragments by cDNA -AFLP screens and verification by RT-PCR in *Arabidopsis* and cotton, and these expression changes could be variable in different parts of the plants (Adams et al., 2003; Wang et al., 2004). Recently, reciprocal gene silencing and novel expression were also found both in natural and synthetic cottons by using RNA-Seq, and specifically increased in natural allopolyploids (Yoo et al., 2013).

To explore patterns of novel gene expression and silencing during the *Brassica* allopolyploidization, synthetic *Brassica* allotetraploids (U 1935) were examined using RNA-Seq data, since RNA-Seq technology could provide more data than cDNA -AFLP and RT-PCR. By restrictive criteria, the percentage of genes showing novel expression and silencing was significantly less than that of *Arabidopsis* and cotton using AFLP-cDNA and RT-PCR method (Adams et al., 2003; Wang et al., 2004), while similar to that of cotton from RNA-Seq (Yoo et al., 2013). This difference may be explained by technical consideration and various restrictive criteria. As a check in present study, we have also validated of five genes using qRT-PCR, four genes and one gene, respectively, were confirmed as novel expression and silencing (Supplementary Figure 4). Our results also found a wide range of variations between the two different expression patterns and different tissues of the allotetraploids (Table 4). More genes displayed novel expression or silencing in silique walls than in leaves, suggesting that tissue-specific expression partitioning could arise quickly after the onset of allotetraploids formation, which was consistent with the previous study (Adams et al., 2003; Wang et al., 2004; Adams and Wendel, 2005; Buggs et al., 2009). Interestingly, there was no significant difference between the total number of genes, but inverse correlation between the two expression patterns was observed among samples, indicating that there was a tradeoff between novel gene expression and silencing during plant development (Figure 5).

### Cytoplasmic effects on genes expression in reciprocal *B. napus*

The cytoplasm has exhibited considerable influence on the evolution of nuclear genomes of allopolyploids (Prakash et al., 2009), because the presence of the paternal nuclear genome in the maternal cytoplasm could result in nuclear-cytoplasmic incompatibilities. Extensive and rapid genomic changes as well as the variations in chromosome meiotic pairings were observed in the reciprocal *Brassica* allopolyploids, but the gene expression changes were not as obvious as the genomic changes (Song et al., 1995; Cui et al., 2012, 2013). Although large-scale differences in gene expression existed between the reciprocal synthetics of *B. napus* (AACC and CCAA), the numbers of genes showing co-up and co-down regulation gave insignificant difference (Table 4). It was interesting to see that these co-regulated genes (co-up and co-down) were enriched in the similar functional classes with similar numbers, revealing that certain gene networks may be particularly susceptible by cytoplasm (Figure 6). Overall, the equivalence in either number or function of co-regulated genes indicated that there might be a balance of differentially expressed genes in nuclear-cytoplasmic interactions.

### Tissue-specific and differential expression of r-protein genes

Although the ribosome was well-known to be an immensely complex “molecular machine” in translating the genetic code, recent studies indicated that the r-protein genes were likely involved in tissue-specific process and had a regulatory role in the development of plant (Byrne, 2009; Whittle and Krochko, 2009). Our data indicated that those sets of r-protein genes which represented a wide range of r-proteins were differently expressed between tissues (Supplementary Table 4). In general, the average expression level of r-protein genes in leaves was higher than that in silique walls (Supplementary Figure 3). Nevertheless, an early finding showed that r-protein gene expression was greatest among highly differentiating reproductive tissues (not including silique walls), for example, microspores, embryos, and seeds, using available EST data. However, the lowest levels were also detected in some reproductive tissues, such as anthers, pollen, seed coats (Whittle and Krochko, 2009). Our results demonstrated that the r-protein gene expression levels in silique walls were lower than that in leaves and such difference could be attributable to the fact that silique walls were more mature and the translation activity was not as high as in young leaves. Additionally, in two *Brassica* allotetraploids with B-genome (AABB and BBCC), the expression level of r-protein genes which were highest among samples in leaves turned to be lowest in silique walls. An recent study in the same synthesized allotetraploids showed higher number and percentage of absent bands of B genome than that of A or C genome in AABB and BBCC using AFLP-cDNA, suggesting that gene expression was particularly susceptible to perturbation when B-genome was present in *Brassica* allotetraploids during plant development (Cui et al., 2013).

Hierarchical clustering of these genes indicated that the expression relationships among *Brassica* allotetraploids and diploids were diverse in different tissues (Figures 7, 8). The hierarchy of r-protein genes in leaves (B > A > C) was consistent with that of the expression of rRNA genes (Chen and Pikaard, 1997). However, the expression of rRNA genes could be developmentally regulated and tissue-specific, because the rRNA genes silenced in vegetative tissues were found to be expressed in reproductive tissues, including sepals and petals (Chen and Pikaard, 1997). Thus, the expression divergence of r-protein genes could be associated with the expression change of parental rRNA genes.

In summary, widespread changes of gene expression were observed among the serial resynthesized *Brassica* allotetraploids relative to their diploid progenitors. There were considerable alterations on temporal and spatial expressions, and the range of variations in silique walls was much wider than in leaves. The expression of r-protein genes was tissue-specific and associated with nucleolar dominance. Furthermore, novel gene expression was negatively correlated with silencing during the transition from vegetative to reproductive development. The balance between the transgressive up- and down-expressions was also observed in leaves, as well as between the co-up and co-down regulation in different cytoplasm, such profound changes might enhance the fitness and adaptability of *Brassica* allopolyploids.

### Conflict of interest statement

The reviewer Genlou Sun declares that, despite having previously collaborated with the authors Xianhong Ge, Yujiao Shao, and Zaiyun Li, the review process was handled objectively. The authors declare that the research was conducted in the absence of any commercial or financial relationships that could be construed as a potential conflict of interest.

## Supplementary material

Supplementary Figure 1 **Analysis of the differentially expressed genes between each allotetraploid and its parents. (A)** Leaves. **(B)** Slique walls.

Supplementary Figure 2 **GO functional categories of genes showing novel expression and silencing. (A)** Leaves. **(B)** Slique walls.

Supplementary Figure 3 **The average expression level of total r-protein genes among samples**.

Supplementary Figure 4 **RT-PCR confirmation of the differentially expressed genes**. Columns and bars represent the means and standard error (*n* = 3), respectively. The gene expression levels from RNA-Seq data are added on the top of each gene.

Supplementary Table 1 **Summary of alignment statistics of RNA-Seq in the 14 samples**.

Supplementary Table 2 **Top 10 GO items of transgressively regulated genes**.

Supplementary Table 3 **Annotation of co-transgressively regulated genes among allotetraploids**.

Supplementary Table 4 **R-protein genes matched each *Arabidopsis* r-proteins**.

Supplementary Table 5 **The corresponding primers of qRT-PCR**.

## Abbreviations

DEGs: differentially expressed genes
FC: fold change
GO: gene ontology.
