# Supplementary figures and images for: Genome-specific differential gene expressions in resynthesized Brassica allotetraploids from pair-wise crosses of three cultivated diploids revealed by RNA-seq
Source: Front Plant Sci. 2015 Nov 4;6:957. doi: 10.3389/fpls.2015.00957 (PMC4631939; doi:10.3389/fpls.2015.00957)

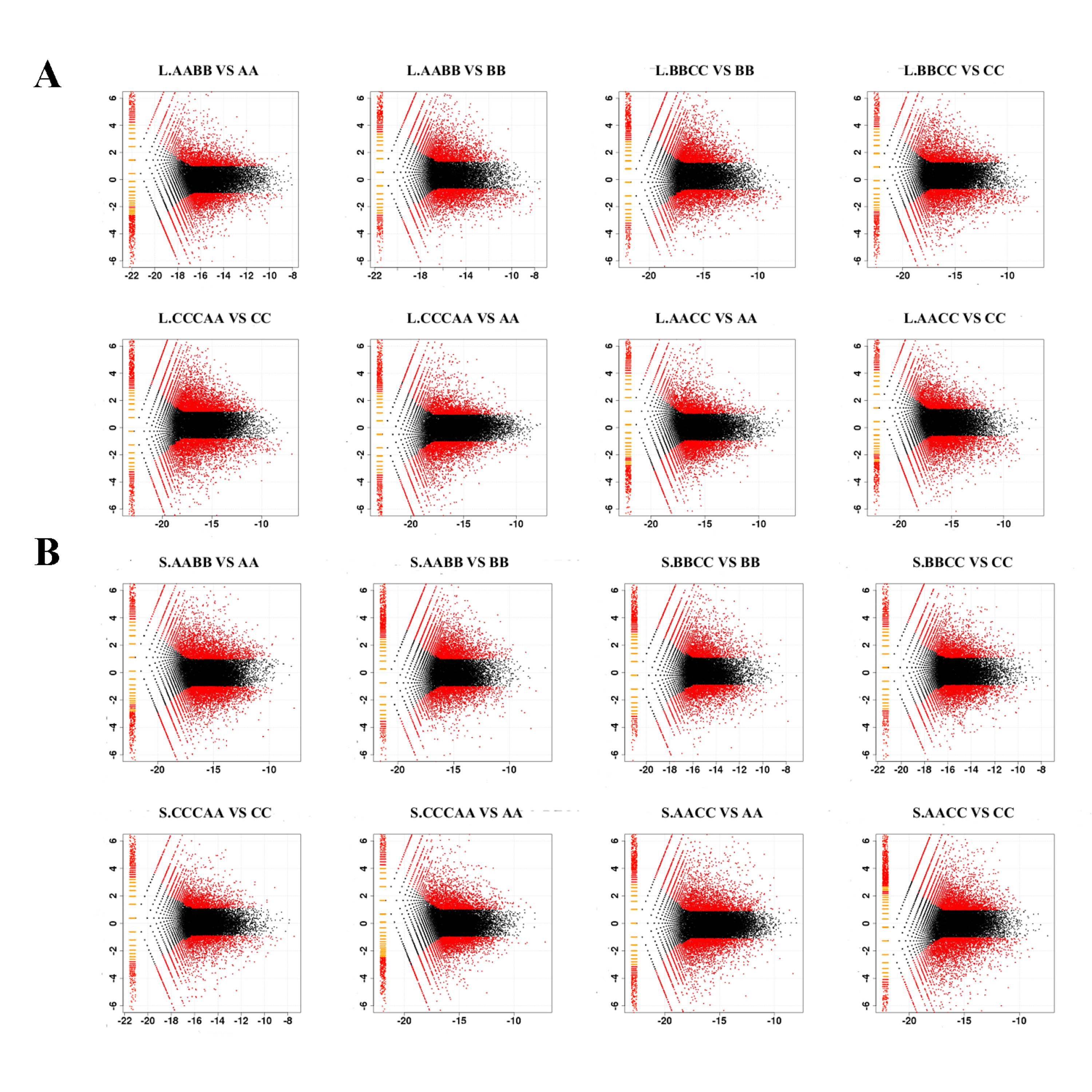

Supplement: Supplementary Figure 1 — Analysis of the differentially expressed genes between each allotetraploid and its parents. (A) Leaves. (B) Slique walls. [file Image1.JPEG]

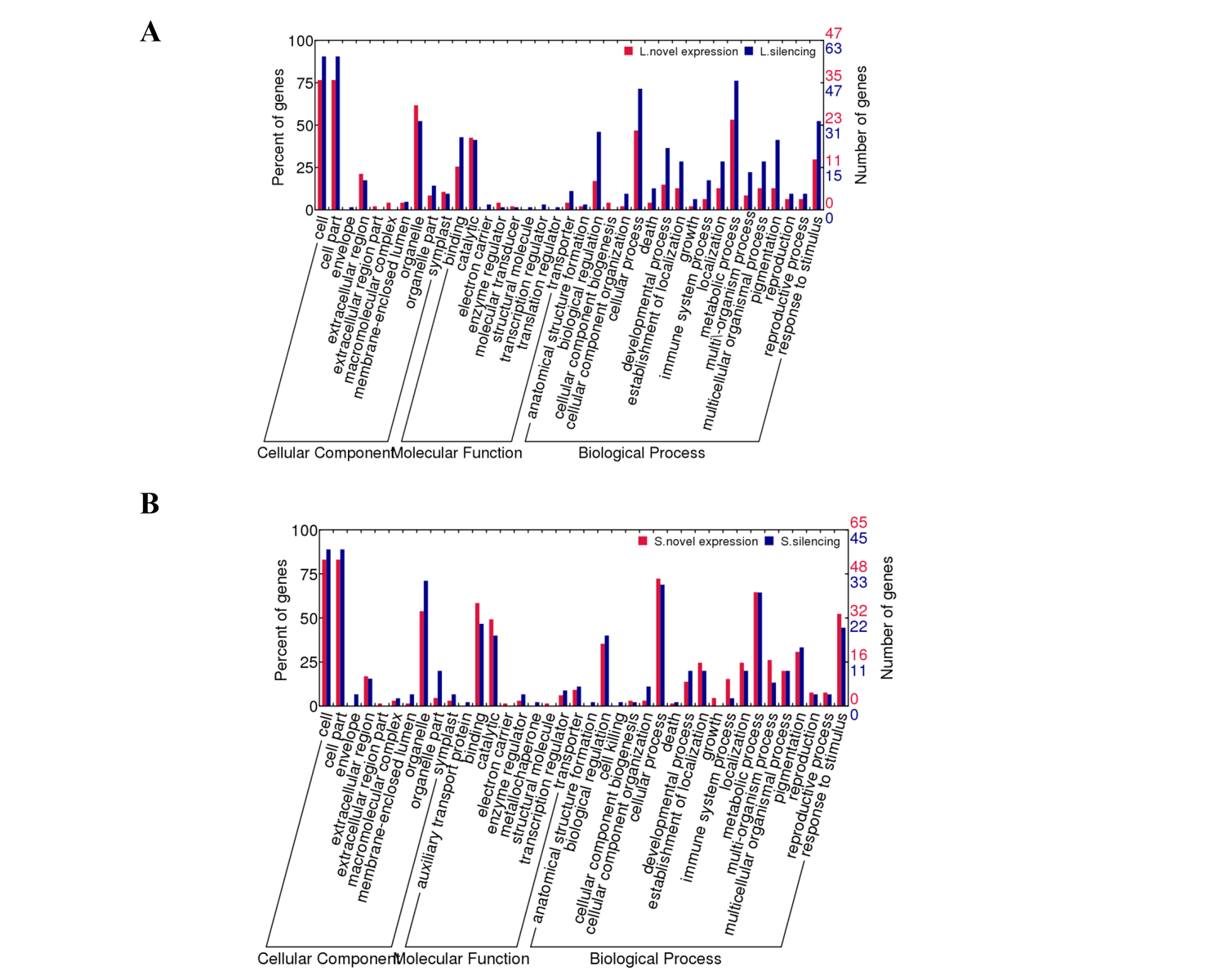

Supplement: Supplementary Figure 2 — GO functional categories of genes showing novel expression and silencing. (A) Leaves. (B) Slique walls. [file Image2.TIF]

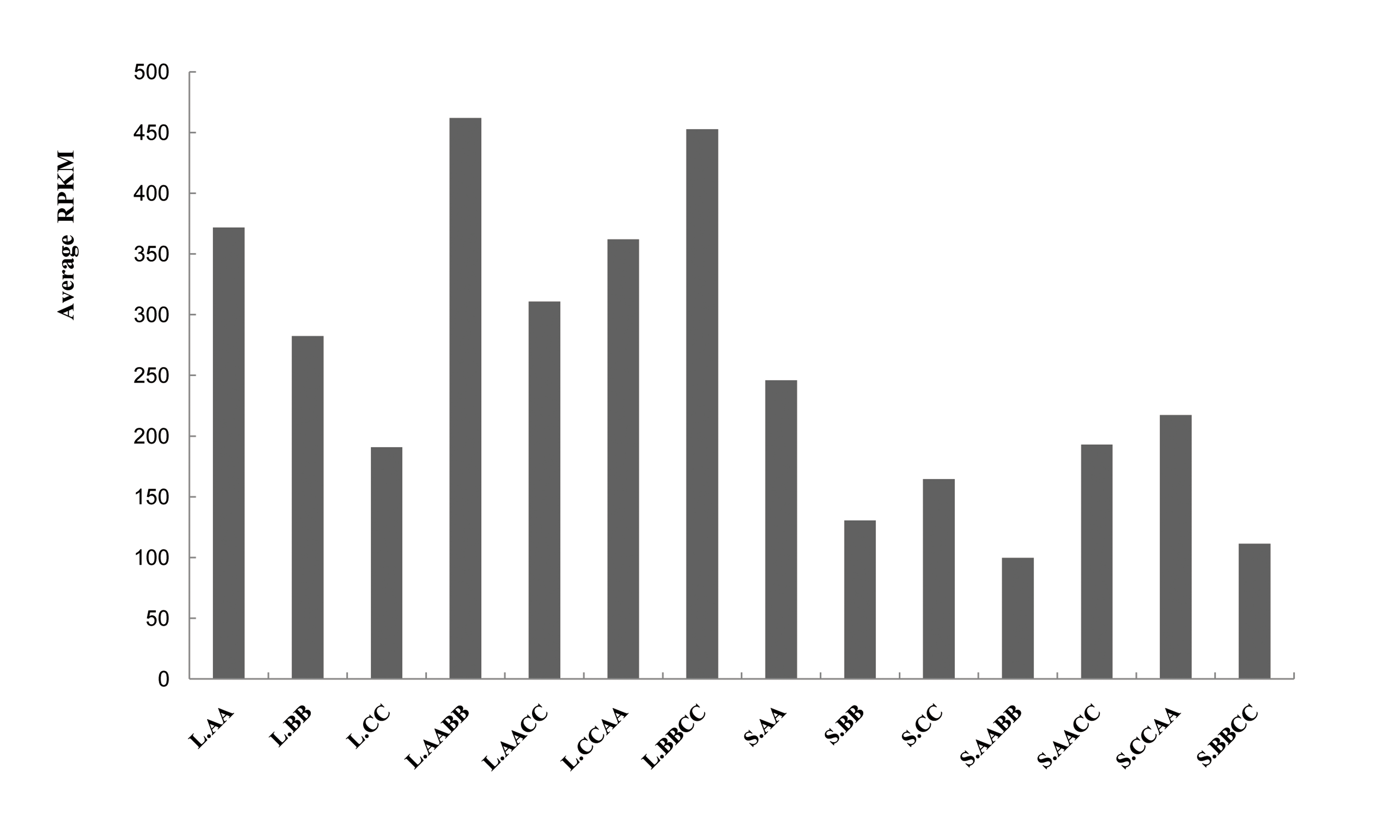

Supplement: Supplementary Figure 3 — The average expression level of total r-protein genes among samples. [file Image3.TIF]
