# Supplementary material for: Genome-specific differential gene expressions in resynthesized Brassica allotetraploids from pair-wise crosses of three cultivated diploids revealed by RNA-seq
Source: Front Plant Sci. 2015 Nov 4;6:957. doi: 10.3389/fpls.2015.00957 (PMC4631939; doi:10.3389/fpls.2015.00957)

Supplementary Figure 4. RT-PCR confirmation of the differentially expressed genes. Columns and bars represent the means and standard error (n = 3), respectively. The gene expression levels from RNA-Seq data are added on the top of each gene.

30.86	0.13	20.74	0	55.47	0	0	128.26	0	7.9	32.41	5.36	7.41	18.43	6.66

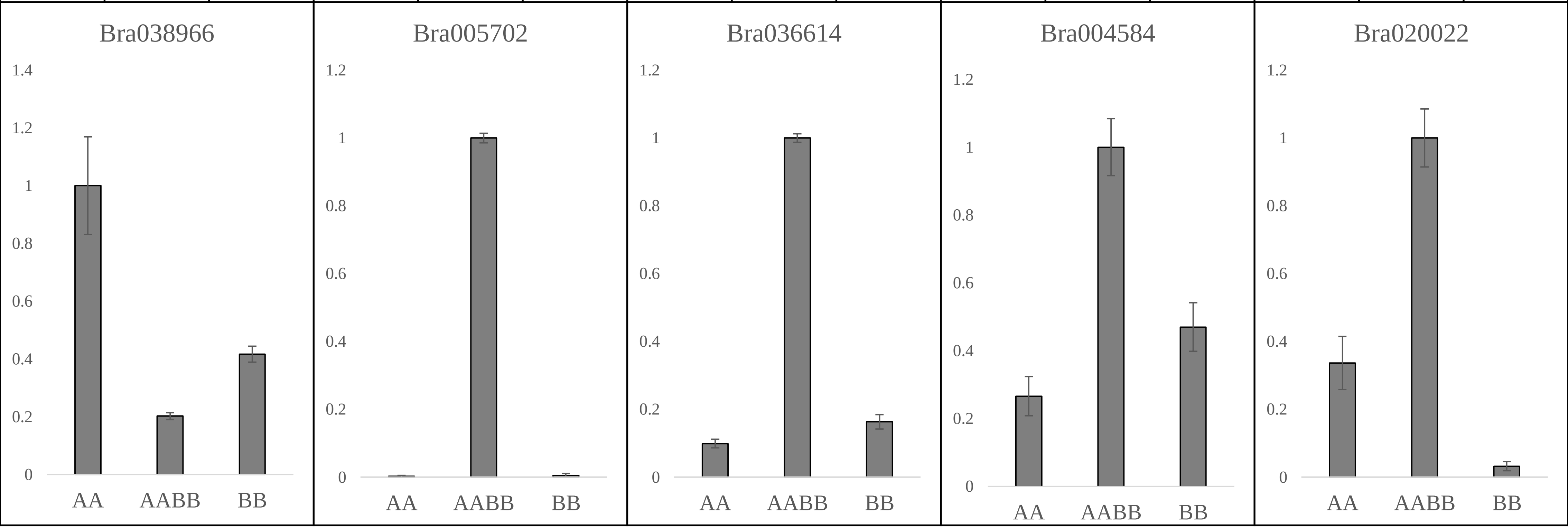

1.57	9.56	2.92	11.97	0	18.46	179.07	37.4	261.97	6.77	2.19	14.05	0.67	55.07	28.09

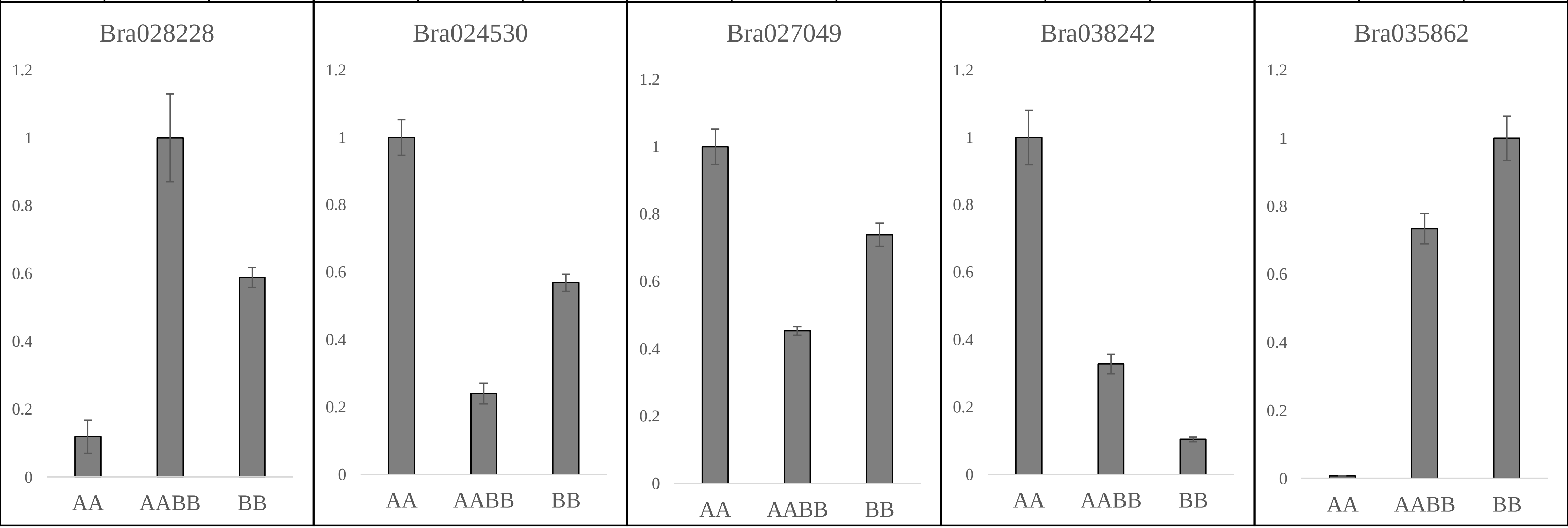

**0**	**59.89**	**0**	**0**	**12.31**	**0**	**179.07**	**63.31**	**212.37**	**2.92**	**10.47**	**0.15**	**56.38**	**24.53**	**353.09**

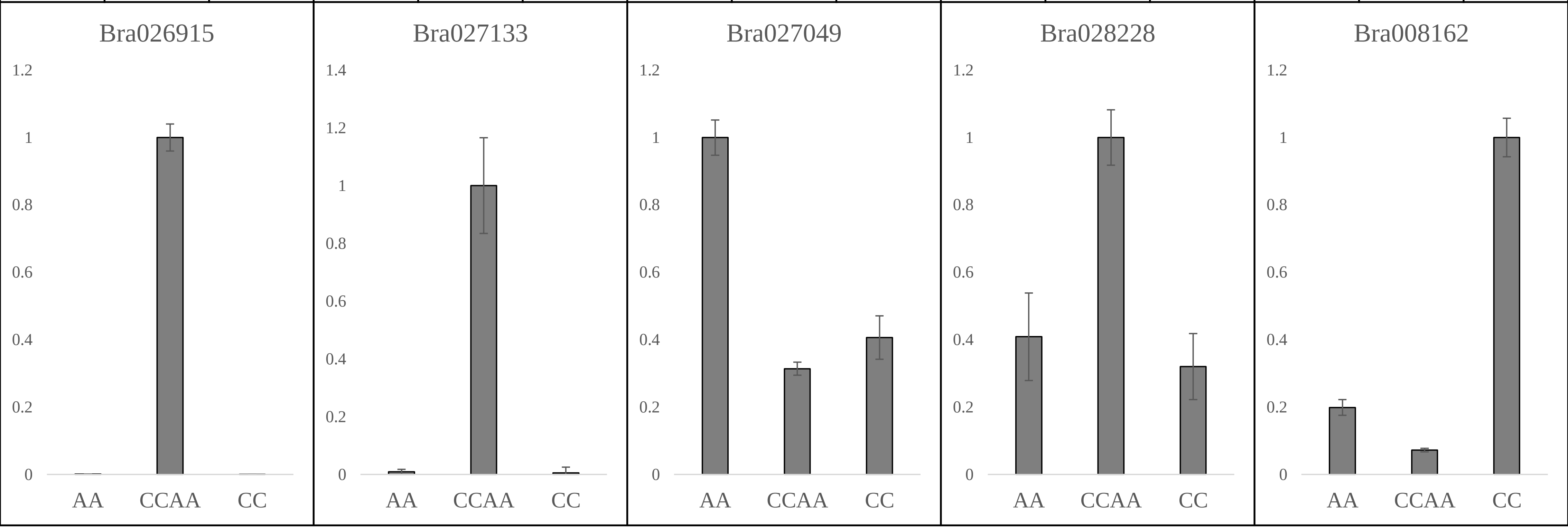

**6.77**	**1.34**	**21.22**	**7.41**	**25.33**	**4.03**	**7.91**	**19.2**	**3.63**	**2.85**	**77.96**	**1.80**	**60.12**	**109.16**	**42.73**

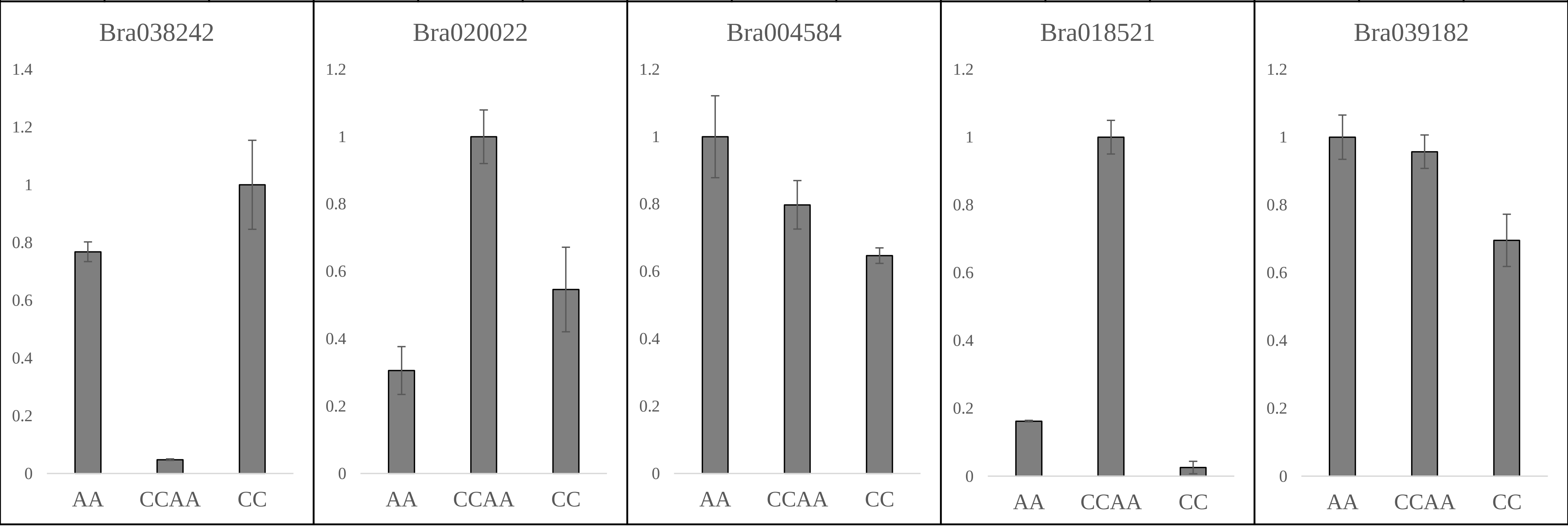

56.38	35.96	353.09	2.22	31.39	1.80	14.05	5.33	21.22	19.48	3.04	18.46	6.66	25.61	4.03

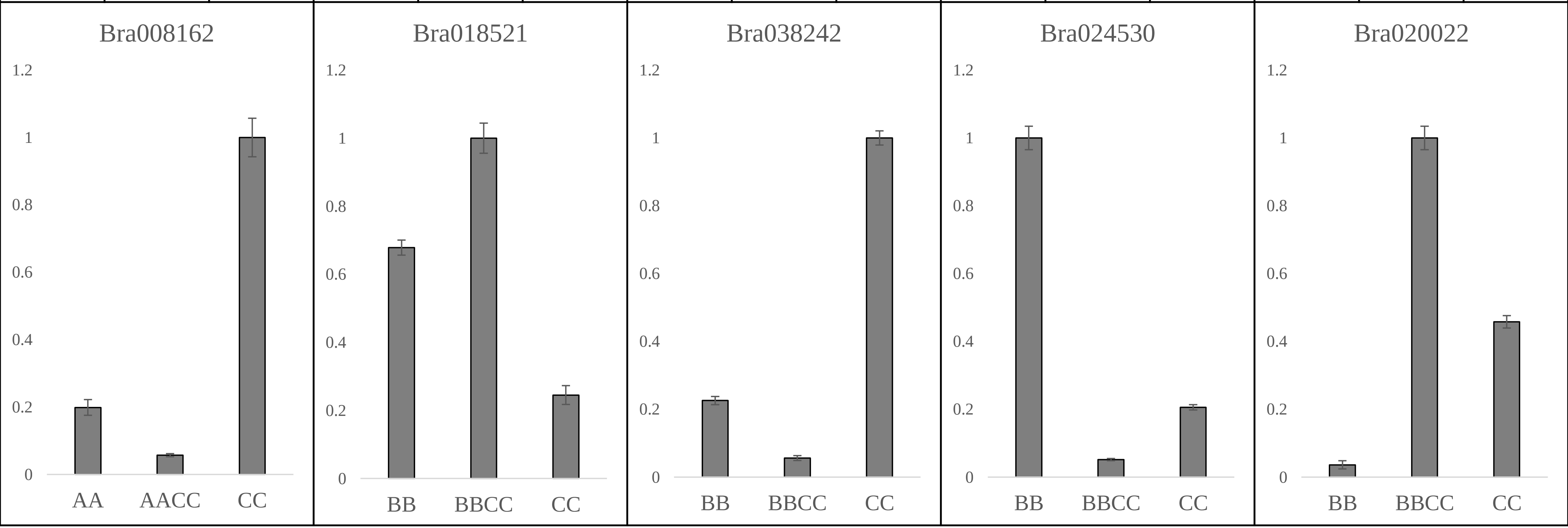

**39.96**	**4.73**	**5.29**	**11**	**4.12**	**25.33**	**0.73**	**10.47**	**0**	**17.05**

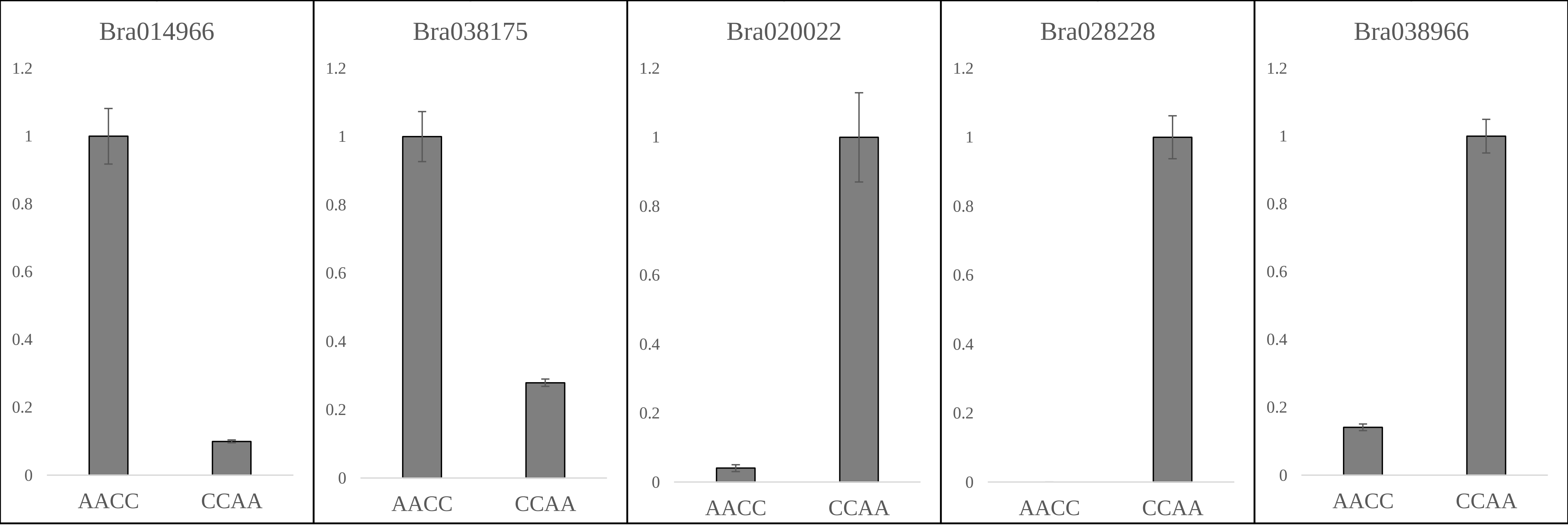

Supplement: Supplementary Figure 4 — RT-PCR confirmation of the differentially expressed genes. Columns and bars represent the means and standard error (n = 3), respectively. The gene expression levels from RNA-Seq data are added on the top of each gene. [file Image4.PDF]
