# Supplementary material for: Genome-specific differential gene expressions in resynthesized Brassica allotetraploids from pair-wise crosses of three cultivated diploids revealed by RNA-seq
Source: Front Plant Sci. 2015 Nov 4;6:957. doi: 10.3389/fpls.2015.00957 (PMC4631939; doi:10.3389/fpls.2015.00957)
Supplement: Supplementary Table 1 — Summary of alignment statistics of RNA-Seq in the 14 samples. [file Table1.DOC]

**Supplementary Table 1. Summary of alignment statistics of RNA-Seq in the 14 samples.**

| Sample^a^ | Clean reads | Unique mapped | expressed genes^b^ |
| --- | --- | --- | --- |
| L.AA | 9012106 | 6213580 | 28707(69.98%) |
| L.BB | 15700561 | 3134171 | 28353(69.12%) |
| L.CC | 16096161 | 6864032 | 29419(71.72%) |
| L.AABB | 16231589 | 2245884 | 27707(67.55%) |
| L.BBCC | 11668931 | 3619251 | 29015(70.73%) |
| L.CCAA | 14849065 | 8400626 | 29744(72.51%) |
| L.AACC | 16439906 | 2636941 | 28268(68.91%) |
| S.AA | 12020481 | 6542484 | 29695(72.39%) |
| S.BB | 9543315 | 1696594 | 27791(67.75%) |
| S.CC | 8491396 | 2614543 | 28103(68.51%) |
| S.AABB | 9406265 | 3000812 | 29491(71.89%) |
| S.BBCC | 13270297 | 2005119 | 29987(73.10%) |
| S.CCAA | 13768351 | 2052375 | 28193(68.73%) |
| S.AACC | 11984349 | 6318583 | 30739(74.49%) |
| Average | 12748770 | 3696642 | 28944(70.56%) |

^a^L.is short for young leaves , S. is short for silique wall , respectively.

^b^The genes with at least one unique mapped reads.
