# Supplementary material for: Genome-specific differential gene expressions in resynthesized Brassica allotetraploids from pair-wise crosses of three cultivated diploids revealed by RNA-seq
Source: Front Plant Sci. 2015 Nov 4;6:957. doi: 10.3389/fpls.2015.00957 (PMC4631939; doi:10.3389/fpls.2015.00957)
Supplement: Supplementary Table 2 — Top 10 GO items of transgressively regulated genes. [file Table2.DOC]

**Supplementary Table 2.** **Top ten GO items of transgressively regulated genes.**

**Top ten GO terms of transgressively up- regulated genes** in L.AABB

| **Term^a^** | **Count** | **%^b^** | **P-Value** |
| --- | --- | --- | --- |
| Cluster 1 ;Enrichment Score: 6.99 | | | |
| GO:0043228~non-membrane-bounded organelle | 68 | 12.90 | 2.85E-15 |
| GO:0043232~intracellular non-membrane-bounded organelle | 68 | 12.90 | 2.85E-15 |
| GO:0030529~ribonucleoprotein complex | 44 | 8.35 | 3.47E-11 |
| ribonucleoprotein | 32 | 6.07 | 4.73E-11 |
| GO:0005840~ribosome | 36 | 6.83 | 4.97E-11 |
| GO:0003735~structural constituent of ribosome | 30 | 5.69 | 1.48E-09 |
| ribosomal protein | 28 | 5.31 | 2.67E-09 |
| GO:0005198~structural molecule activity | 34 | 6.45 | 1.01E-08 |
| GO:0033279~ribosomal subunit | 24 | 4.55 | 4.99E-08 |
| GO:0044445~cytosolic part | 21 | 3.98 | 9.70E-07 |
| GO:0022626~cytosolic ribosome | 22 | 4.17 | 2.92E-06 |
| ath03010:Ribosome | 18 | 3.42 | 8.14E-05 |
| GO:0015934~large ribosomal subunit | 13 | 2.47 | 2.65E-04 |
| GO:0005829~cytosol | 30 | 5.69 | 3.70E-04 |
| GO:0015935~small ribosomal subunit | 11 | 2.09 | 4.53E-04 |
| GO:0022627~cytosolic small ribosomal subunit | 10 | 1.90 | 6.10E-04 |
| GO:0022625~cytosolic large ribosomal subunit | 10 | 1.90 | 0.002343 |
|  |  |  |  |
| Cluster 2; Enrichment Score: 5.07 | | | |
| GO:0005730~nucleolus | 22 | 4.17 | 6.02E-06 |
| GO:0070013~intracellular organelle lumen | 33 | 6.26 | 6.79E-06 |
| GO:0043233~organelle lumen | 33 | 6.26 | 6.79E-06 |
| GO:0031974~membrane-enclosed lumen | 33 | 6.26 | 8.74E-06 |
| GO:0031981~nuclear lumen | 26 | 4.93 | 1.75E-05 |
|  |  |  |  |
| Cluster 3; Enrichment Score: 2.52 | | | |
| GO:0006333~chromatin assembly or disassembly | 10 | 1.90 | 1.11E-04 |
| nucleosome core | 7 | 1.33 | 2.62E-04 |
| IPR007125:Histone core | 7 | 1.33 | 3.26E-04 |
| GO:0044427~chromosomal part | 12 | 2.28 | 4.13E-04 |
| GO:0005694~chromosome | 14 | 2.66 | 4.70E-04 |
| GO:0031497~chromatin assembly | 8 | 1.52 | 5.77E-04 |
| GO:0000785~chromatin | 9 | 1.71 | 5.98E-04 |
| GO:0006323~DNA packaging | 8 | 1.52 | 9.40E-04 |
| chromosomal protein | 7 | 1.33 | 0.001018 |
| IPR009072:Histone-fold | 8 | 1.52 | 0.001329 |
| GO:0000786~nucleosome | 7 | 1.33 | 0.001912 |
| SM00414:H2A | 4 | 0.76 | 0.002575 |
| IPR002119:Histone H2A | 4 | 0.76 | 0.002595 |
| GO:0006334~nucleosome assembly | 7 | 1.33 | 0.002719 |
| GO:0034728~nucleosome organization | 7 | 1.33 | 0.002719 |
| GO:0032993~protein-DNA complex | 7 | 1.33 | 0.003271 |
| GO:0065004~protein-DNA complex assembly | 7 | 1.33 | 0.00337 |
| GO:0006325~chromatin organization | 12 | 2.28 | 0.005214 |
| GO:0051276~chromosome organization | 12 | 2.28 | 0.016453 |
| GO:0034622~cellular macromolecular complex assembly | 10 | 1.90 | 0.026161 |
| PIRSF002048:histone H2A | 3 | 0.57 | 0.032896 |
| GO:0034621~cellular macromolecular complex subunit organization | 10 | 1.90 | 0.041753 |
| GO:0065003~macromolecular complex assembly | 11 | 2.09 | 0.048878 |
|  |  |  |  |
| Cluster 4; Enrichment Score: 2.26 | | | |
| GO:0009536~plastid | 90 | 17.08 | 0.002149 |
| GO:0009507~chloroplast | 87 | 16.51 | 0.003926 |
| GO:0044435~plastid part | 34 | 6.45 | 0.00432 |
| GO:0044434~chloroplast part | 33 | 6.26 | 0.004875 |
| GO:0009532~plastid stroma | 18 | 3.42 | 0.011153 |
| GO:0009570~chloroplast stroma | 17 | 3.23 | 0.014852 |
|  |  |  |  |
| Cluster 5; Enrichment Score: 1.79 | | | |
| GO:0044429~mitochondrial part | 14 | 2.66 | 0.013254 |
| GO:0031980~mitochondrial lumen | 6 | 1.14 | 0.017751 |
| GO:0005759~mitochondrial matrix | 6 | 1.14 | 0.017751 |
|  |  |  |  |
| Cluster 6; Enrichment Score: 1.54 | | | |
| GO:0007623~circadian rhythm | 6 | 1.14 | 0.001086 |
| GO:0048511~rhythmic process | 6 | 1.14 | 0.003051 |
| domain:CCT | 4 | 0.76 | 0.016239 |
| domain:Response regulatory | 4 | 0.76 | 0.03313 |
| IPR010402:CCT domain | 4 | 0.76 | 0.047763 |
| two-component regulatory system | 4 | 0.76 | 0.048673 |
|  |  |  |  |
| Cluster 7; Enrichment Score: 1.13 | | | |
| GO:0009532~plastid stroma | 18 | 3.42 | 0.011153 |
| transit peptide | 28 | 5.31 | 0.046907 |
| transit peptide:Chloroplast | 19 | 3.61 | 0.098825 |
| chloroplast | 20 | 3.80 | 0.194916 |
| plastid | 19 | 3.61 | 0.222659 |
|  |  |  |  |
| Cluster 8; Enrichment Score: 0.94 | | | |
| region of interest:Type E(+) motif | 8 | 1.52 | 0.026826 |
| repeat:PPR 10 | 13 | 2.47 | 0.048783 |
|  |  |  |  |

^a^ GO terms with enrichment score>0.5 and P<0.05 were considered to be significantly enriched.

^b^ Percentage of total [functional](javascript:void(0);) [annotation](javascript:void(0);) genes.

**Top ten GO terms of transgressively down- regulated genes in L.AABB**

| **Term^a^** | **Count** | **%^b^** | **P-Value** |
| --- | --- | --- | --- |
| Cluster 1; Enrichment Score: 3.85 | | | |
| GO:0010038~response to metal ion | 23 | 5.00 | 4.90E-05 |
| GO:0046686~response to cadmium ion | 20 | 4.35 | 1.34E-04 |
| GO:0010035~response to inorganic substance | 26 | 5.65 | 4.41E-04 |
|  |  |  |  |
| Cluster 2; Enrichment Score: 3.61 | | | |
| GO:0005618~cell wall | 33 | 7.17 | 3.91E-06 |
| GO:0030312~external encapsulating structure | 33 | 7.17 | 5.30E-06 |
| GO:0009505~plant-type cell wall | 14 | 3.04 | 0.006337 |
| GO:0005576~extracellular region | 38 | 8.26 | 0.025992 |
|  |  |  |  |
| Cluster 3; Enrichment Score: 3.50 | | | |
| GO:0009814~defense response, incompatible interaction | 13 | 2.83 | 1.38E-06 |
| GO:0042742~defense response to bacterium | 16 | 3.48 | 1.53E-05 |
| GO:0009617~response to bacterium | 18 | 3.91 | 3.26E-05 |
| GO:0009627~systemic acquired resistance | 6 | 1.30 | 6.45E-04 |
| GO:0006955~immune response | 16 | 3.48 | 0.00235 |
| GO:0045087~innate immune response | 15 | 3.26 | 0.003407 |
|  |  |  |  |
| Cluster 4; Enrichment Score: 2.91 | | | |
| membrane | 69 | 15.00 | 6.98E-06 |
| cell membrane | 21 | 4.57 | 2.94E-05 |
| transmembrane | 57 | 12.39 | 9.54E-05 |
| transport | 37 | 8.04 | 1.02E-04 |
| topological domain:Extracellular | 20 | 4.35 | 0.003633 |
| topological domain:Cytoplasmic | 25 | 5.43 | 0.007038 |
| transmembrane region | 53 | 11.52 | 0.008748 |
| GO:0016021~integral to membrane | 60 | 13.04 | 0.045898 |
|  |  |  |  |
| Cluster 5; Enrichment Score: 2.67 | | | |
| GO:0000272~polysaccharide catabolic process | 8 | 1.74 | 0.001258 |
| GO:0016052~carbohydrate catabolic process | 13 | 2.83 | 0.00204 |
| GO:0005976~polysaccharide metabolic process | 13 | 2.83 | 0.003631 |
|  |  |  |  |
| Cluster 6; Enrichment Score: 2.30 | | | |
| GO:0009628~response to abiotic stimulus | 46 | 10.00 | 3.25E-04 |
| GO:0009416~response to light stimulus | 21 | 4.57 | 0.003885 |
| GO:0009314~response to radiation | 21 | 4.57 | 0.005589 |
|  |  |  |  |
| Cluster 7; Enrichment Score: 2.24 | | | |
| GO:0009064~glutamine family amino acid metabolic process | 8 | 1.74 | 3.52E-04 |
| ath00330:Arginine and proline metabolism | 6 | 1.30 | 0.011284 |
| GO:0006525~arginine metabolic process | 3 | 0.65 | 0.048099 |
|  |  |  |  |
| Cluster 8; Enrichment Score: 2.21 | | | |
| GO:0009627~systemic acquired resistance | 6 | 1.30 | 6.45E-04 |
| GO:0031348~negative regulation of defense response | 4 | 0.87 | 0.005861 |
|  |  |  |  |
| Cluster 9; Enrichment Score: 2.16 | | | |
| Symport | 9 | 1.96 | 2.60E-05 |
| amino-acid transport | 5 | 1.09 | 4.61E-04 |
| GO:0015171~amino acid transmembrane transporter activity | 6 | 1.30 | 0.014462 |
| GO:0006865~amino acid transport | 6 | 1.30 | 0.015534 |
| GO:0015837~amine transport | 6 | 1.30 | 0.016491 |
| GO:0005275~amine transmembrane transporter activity | 6 | 1.30 | 0.021639 |
| GO:0046942~carboxylic acid transport | 6 | 1.30 | 0.028258 |
| GO:0015849~organic acid transport | 6 | 1.30 | 0.028258 |
| IPR013057:Amino acid transporter, transmembrane | 4 | 0.87 | 0.048505 |
|  |  |  |  |
| Cluster 10; Enrichment Score: 2.08 | | | |
| peroxisome | 6 | 1.30 | 0.002331 |
| GO:0005777~peroxisome | 10 | 2.17 | 0.015514 |
| GO:0042579~microbody | 10 | 2.17 | 0.015514 |

^a^ GO terms with enrichment score>0.5 and P<0.05 were considered to be significantly enriched.

^b^ Percentage of total [functional](javascript:void(0);) [annotation](javascript:void(0);) genes.

**Top ten GO terms of transgressively up- regulated genes in L.**BBCC

| **Term^a^** | **Count** | **%^b^** | **P-Value** |
| --- | --- | --- | --- |
| Cluster 1; Enrichment Score: 13.06 | | | |
| GO:0015630~microtubule cytoskeleton | 31 | 4.19 | 3.96E-16 |
| GO:0007018~microtubule-based movement | 23 | 3.11 | 5.26E-16 |
| GO:0044430~cytoskeletal part | 33 | 4.47 | 1.40E-15 |
| GO:0005856~cytoskeleton | 36 | 4.87 | 7.40E-15 |
| GO:0007017~microtubule-based process | 26 | 3.52 | 3.13E-14 |
| GO:0005874~microtubule | 25 | 3.38 | 7.72E-14 |
| GO:0003777~microtubule motor activity | 20 | 2.71 | 1.46E-13 |
| motor protein | 20 | 2.71 | 1.79E-13 |
| IPR001752:Kinesin, motor region | 18 | 2.44 | 1.86E-13 |
| SM00129:KISc | 18 | 2.44 | 3.61E-13 |
| microtubule | 21 | 2.84 | 6.79E-13 |
| GO:0003774~motor activity | 22 | 2.98 | 1.98E-12 |
| GO:0005875~microtubule associated complex | 17 | 2.30 | 2.12E-12 |
| IPR019821:Kinesin, motor region, conserved site | 15 | 2.03 | 4.70E-11 |
|  |  |  |  |
| Cluster 2; Enrichment Score: 8.37 | | | |
| GO:0007049~cell cycle | 39 | 5.28 | 2.52E-17 |
| GO:0051301~cell division | 33 | 4.47 | 1.11E-16 |
| cell division | 21 | 2.84 | 5.10E-12 |
| cell cycle | 22 | 2.98 | 6.20E-12 |
| GO:0051726~regulation of cell cycle | 22 | 2.98 | 3.14E-11 |
| IPR004367:Cyclin, C-terminal | 12 | 1.62 | 5.32E-10 |
| IPR014400:Cyclin, A/B/D/E | 10 | 1.35 | 2.83E-09 |
| PIRSF001771:Cyclin_A_B_D_E | 10 | 1.35 | 2.09E-08 |
| cell cycle control | 7 | 0.95 | 3.19E-08 |
| GO:0016538~cyclin-dependent protein kinase regulator activity | 11 | 1.49 | 6.37E-08 |
| PIRSF001771:cyclin, A/B/D/E types | 9 | 1.22 | 9.33E-08 |
| IPR006671:Cyclin, N-terminal | 12 | 1.62 | 1.14E-07 |
| cyclin | 12 | 1.62 | 1.57E-07 |
| GO:0019887~protein kinase regulator activity | 11 | 1.49 | 5.69E-07 |
| IPR006670:Cyclin | 12 | 1.62 | 5.86E-07 |
| GO:0019207~kinase regulator activity | 11 | 1.49 | 9.06E-07 |
| SM00385:CYCLIN | 12 | 1.62 | 9.55E-07 |
| IPR013763:Cyclin-related | 9 | 1.22 | 4.57E-05 |
| cell division control | 4 | 0.54 | 1.95E-04 |
|  |  |  |  |
| Cluster 3; Enrichment Score: 8.25 | | | |
| GO:0043232~intracellular non-membrane-bounded organelle | 110 | 14.88 | 2.64E-29 |
| GO:0043228~non-membrane-bounded organelle | 110 | 14.88 | 2.64E-29 |
| ribosomal protein | 36 | 4.87 | 8.10E-11 |
| GO:0022626~cytosolic ribosome | 35 | 4.74 | 8.81E-11 |
| ribonucleoprotein | 37 | 5.01 | 2.19E-10 |
| GO:0044445~cytosolic part | 31 | 4.19 | 5.48E-10 |
| GO:0005840~ribosome | 42 | 5.68 | 6.10E-10 |
| GO:0003735~structural constituent of ribosome | 38 | 5.14 | 3.88E-09 |
| GO:0005198~structural molecule activity | 43 | 5.82 | 7.34E-08 |
| GO:0030529~ribonucleoprotein complex | 47 | 6.36 | 1.03E-07 |
| GO:0033279~ribosomal subunit | 28 | 3.79 | 2.10E-07 |
| GO:0005829~cytosol | 46 | 6.22 | 1.21E-06 |
| GO:0022625~cytosolic large ribosomal subunit | 15 | 2.03 | 6.28E-05 |
| GO:0022627~cytosolic small ribosomal subunit | 13 | 1.76 | 1.46E-04 |
| GO:0022613~ribonucleoprotein complex biogenesis | 20 | 2.71 | 2.77E-04 |
| GO:0042254~ribosome biogenesis | 19 | 2.57 | 5.39E-04 |
| GO:0015935~small ribosomal subunit | 13 | 1.76 | 6.13E-04 |
| GO:0015934~large ribosomal subunit | 15 | 2.03 | 6.69E-04 |
| ath03010:Ribosome | 19 | 2.57 | 0.001455 |
|  |  |  |  |
| Cluster 4; Enrichment Score: 4.98 | | | |
| GO:0022402~cell cycle process | 23 | 3.11 | 1.15E-10 |
| GO:0022403~cell cycle phase | 15 | 2.03 | 7.23E-07 |
| GO:0000280~nuclear division | 10 | 1.35 | 3.47E-06 |
| GO:0000279~M phase | 13 | 1.76 | 5.16E-06 |
| mitosis | 7 | 0.95 | 1.14E-05 |
| GO:0000278~mitotic cell cycle | 10 | 1.35 | 2.29E-05 |
| GO:0007067~mitosis | 8 | 1.08 | 1.19E-04 |
| GO:0000087~M phase of mitotic cell cycle | 8 | 1.08 | 1.19E-04 |
| GO:0048285~organelle fission | 10 | 1.35 | 2.08E-04 |
| GO:0030261~chromosome condensation | 3 | 0.41 | 0.013185 |
|  |  |  |  |
| Cluster 5; Enrichment Score: 4.82 | | | |
| GO:0030312~external encapsulating structure | 43 | 5.82 | 5.39E-07 |
| GO:0005618~cell wall | 42 | 5.68 | 9.34E-07 |
| GO:0009505~plant-type cell wall | 17 | 2.30 | 0.007026 |
|  |  |  |  |
| Cluster 6; Enrichment Score: 3.58 | | | |
| GO:0005730~nucleolus | 28 | 3.79 | 2.29E-06 |
| GO:0031981~nuclear lumen | 32 | 4.33 | 3.20E-05 |
| GO:0070013~intracellular organelle lumen | 34 | 4.60 | 0.002429 |
| GO:0043233~organelle lumen | 34 | 4.60 | 0.002429 |
| GO:0031974~membrane-enclosed lumen | 34 | 4.60 | 0.00293 |
|  |  |  |  |
| Cluster 7; Enrichment Score: 3.56 | | | |
| GO:0009309~amine biosynthetic process | 19 | 2.57 | 6.46E-05 |
| GO:0046394~carboxylic acid biosynthetic process | 28 | 3.79 | 1.73E-04 |
| GO:0016053~organic acid biosynthetic process | 28 | 3.79 | 1.73E-04 |
| GO:0008652~cellular amino acid biosynthetic process | 17 | 2.30 | 1.93E-04 |
| GO:0044271~nitrogen compound biosynthetic process | 28 | 3.79 | 0.004231 |
|  |  |  |  |
| Cluster 8; Enrichment Score: 3.28 | | | |
| GO:0010564~regulation of cell cycle process | 8 | 1.08 | 1.02E-05 |
| GO:0007346~regulation of mitotic cell cycle | 5 | 0.68 | 0.001957 |
| GO:0033043~regulation of organelle organization | 6 | 0.81 | 0.007466 |
|  |  |  |  |
| Cluster 9; Enrichment Score: 3.06 | | | |
| GO:0016143~S-glycoside metabolic process | 10 | 1.35 | 6.46E-06 |
| GO:0019760~glucosinolate metabolic process | 10 | 1.35 | 6.46E-06 |
| GO:0019757~glycosinolate metabolic process | 10 | 1.35 | 6.46E-06 |
| GO:0016144~S-glycoside biosynthetic process | 7 | 0.95 | 2.19E-04 |
| GO:0019761~glucosinolate biosynthetic process | 7 | 0.95 | 2.19E-04 |
| GO:0019758~glycosinolate biosynthetic process | 7 | 0.95 | 2.19E-04 |
| GO:0016137~glycoside metabolic process | 11 | 1.49 | 2.58E-04 |
| GO:0019748~secondary metabolic process | 28 | 3.79 | 3.10E-04 |
| GO:0044272~sulfur compound biosynthetic process | 11 | 1.49 | 0.001751 |
| GO:0006790~sulfur metabolic process | 14 | 1.89 | 0.002555 |
| GO:0016138~glycoside biosynthetic process | 7 | 0.95 | 0.012075 |
| sequence variant | 9 | 1.22 | 0.026321 |
| ath00966:Glucosinolate biosynthesis | 4 | 0.54 | 0.030079 |
|  |  |  |  |
| Cluster 10; Enrichment Score: 2.29 | | | |
| GO:0000910~cytokinesis | 9 | 1.22 | 3.06E-05 |
| GO:0000911~cytokinesis by cell plate formation | 6 | 0.81 | 1.70E-04 |
| GO:0033205~cytokinesis during cell cycle | 6 | 0.81 | 3.74E-04 |
| GO:0032506~cytokinetic process | 4 | 0.54 | 0.008297 |
| GO:0031032~actomyosin structure organization | 3 | 0.41 | 0.013185 |
| GO:0000914~phragmoplast formation | 3 | 0.41 | 0.013185 |
| GO:0000912~formation of actomyosin apparatus involved in cytokinesis | 3 | 0.41 | 0.013185 |
| GO:0007010~cytoskeleton organization | 9 | 1.22 | 0.019764 |

^a^ GO terms with enrichment score>0.5 and P<0.05 were considered to be significantly enriched.

^b^ Percentage of total [functional](javascript:void(0);) [annotation](javascript:void(0);) genes.

**Top ten GO terms of transgressively down- regulated genes in L.**BBCC

| **Term^a^** | **Count** | **%^b^** | **P-Value** |
| --- | --- | --- | --- |
| Cluster 1; Enrichment Score: 40.64 | | | |
| GO:0044434~chloroplast part | 160 | 20.81 | 7.46E-63 |
| GO:0044435~plastid part | 162 | 21.07 | 1.51E-62 |
| GO:0055035~plastid thylakoid membrane | 80 | 10.40 | 2.20E-46 |
| GO:0009535~chloroplast thylakoid membrane | 80 | 10.40 | 2.20E-46 |
| GO:0009579~thylakoid | 101 | 13.13 | 2.43E-46 |
| GO:0044436~thylakoid part | 87 | 11.31 | 3.64E-46 |
| GO:0031976~plastid thylakoid | 86 | 11.18 | 6.30E-46 |
| GO:0009534~chloroplast thylakoid | 86 | 11.18 | 6.30E-46 |
| GO:0031984~organelle subcompartment | 86 | 11.18 | 1.03E-45 |
| GO:0042651~thylakoid membrane | 80 | 10.40 | 1.36E-44 |
| GO:0034357~photosynthetic membrane | 81 | 10.53 | 7.98E-43 |
| GO:0015979~photosynthesis | 54 | 7.02 | 1.61E-35 |
| GO:0031090~organelle membrane | 100 | 13.00 | 6.12E-25 |
| GO:0009570~chloroplast stroma | 67 | 8.71 | 1.06E-23 |
| GO:0009532~plastid stroma | 68 | 8.84 | 3.79E-23 |
| thylakoid | 34 | 4.42 | 6.48E-19 |
|  |  |  |  |
| Cluster 2; Enrichment Score: 35.59 | | | |
| GO:0044434~chloroplast part | 160 | 20.81 | 7.46E-63 |
| GO:0044435~plastid part | 162 | 21.07 | 1.51E-62 |
| GO:0009507~chloroplast | 271 | 35.24 | 3.42E-48 |
| GO:0009536~plastid | 274 | 35.63 | 4.50E-48 |
| GO:0009579~thylakoid | 101 | 13.13 | 2.43E-46 |
| chloroplast | 108 | 14.04 | 1.32E-42 |
| transit peptide | 113 | 14.69 | 4.86E-37 |
| plastid | 99 | 12.87 | 6.28E-37 |
| transit peptide:Chloroplast | 95 | 12.35 | 2.27E-36 |
| GO:0009526~plastid envelope | 71 | 9.23 | 3.30E-24 |
| GO:0009570~chloroplast stroma | 67 | 8.71 | 1.06E-23 |
| GO:0009532~plastid stroma | 68 | 8.84 | 3.79E-23 |
| GO:0009941~chloroplast envelope | 67 | 8.71 | 1.49E-22 |
| GO:0031967~organelle envelope | 76 | 9.88 | 3.26E-15 |
| GO:0031975~envelope | 76 | 9.88 | 5.02E-15 |
|  |  |  |  |
| Cluster 3; Enrichment Score: 21.17 | | | |
| GO:0015979~photosynthesis | 54 | 7.02 | 1.61E-35 |
| GO:0019684~photosynthesis, light reaction | 27 | 3.51 | 4.30E-18 |
| GO:0006091~generation of precursor metabolites and energy | 46 | 5.98 | 4.32E-12 |
|  |  |  |  |
| Cluster 4; Enrichment Score: 10.26 | | | |
| thylakoid | 34 | 4.42 | 6.48E-19 |
| photosynthesis | 23 | 2.99 | 4.06E-15 |
| GO:0009521~photosystem | 22 | 2.86 | 1.08E-13 |
| GO:0009523~photosystem II | 16 | 2.08 | 4.41E-10 |
| ath00195:Photosynthesis | 18 | 2.34 | 4.28E-08 |
| photosystem ii | 11 | 1.43 | 5.90E-08 |
| GO:0030095~chloroplast photosystem II | 9 | 1.17 | 1.20E-07 |
| GO:0009654~oxygen evolving complex | 8 | 1.04 | 2.29E-06 |
|  |  |  |  |
| Cluster 5; Enrichment Score: 8.06 | | | |
| GO:0010319~stromule | 14 | 1.82 | 7.60E-11 |
| GO:0009409~response to cold | 29 | 3.77 | 1.05E-08 |
| GO:0009266~response to temperature stimulus | 33 | 4.29 | 8.02E-07 |
|  |  |  |  |
| Cluster 6; Enrichment Score: 6.47 | | | |
| GO:0031977~thylakoid lumen | 23 | 2.99 | 1.48E-12 |
| transit peptide:Thylakoid | 17 | 2.21 | 3.30E-11 |
| GO:0009543~chloroplast thylakoid lumen | 19 | 2.47 | 3.61E-10 |
| GO:0031978~plastid thylakoid lumen | 19 | 2.47 | 3.61E-10 |
| GO:0030095~chloroplast photosystem II | 9 | 1.17 | 1.20E-07 |
|  |  |  |  |
| Cluster 7; Enrichment Score: 6.25 | | | |
| GO:0009628~response to abiotic stimulus | 91 | 11.83 | 1.27E-12 |
| GO:0009416~response to light stimulus | 37 | 4.81 | 6.43E-06 |
| GO:0009314~response to radiation | 37 | 4.81 | 1.37E-05 |
| GO:0009639~response to red or far red light | 16 | 2.08 | 8.82E-04 |
|  |  |  |  |
| Cluster 8; Enrichment Score:6.00 | | | |
| GO:0009767~photosynthetic electron transport chain | 11 | 1.43 | 2.62E-08 |
| GO:0009773~photosynthetic electron transport in photosystem I | 7 | 0.91 | 4.07E-06 |
| GO:0022900~electron transport chain | 20 | 2.60 | 9.42E-06 |
|  |  |  |  |
| Cluster 9; Enrichment Score: 5.93 | | | |
| GO:0019685~photosynthesis, dark reaction | 9 | 1.17 | 1.05E-08 |
| GO:0019253~reductive pentose-phosphate cycle | 8 | 1.04 | 1.64E-07 |
| GO:0015977~carbon utilization by fixation of carbon dioxide | 9 | 1.17 | 1.77E-07 |
| calvin cycle | 6 | 0.78 | 1.26E-05 |
| GO:0016051~carbohydrate biosynthetic process | 21 | 2.73 | 5.88E-04 |
|  |  |  |  |
| Cluster 10; Enrichment Score: 4.88 | | | |
| GO:0009853~photorespiration | 11 | 1.43 | 1.28E-06 |
| photorespiration | 6 | 0.78 | 2.00E-05 |
| GO:0043094~cellular metabolic compound salvage | 11 | 1.43 | 8.69E-05 |

^a^ GO terms with enrichment score>0.5 and P<0.05 were considered to be significantly enriched.

^b^ Percentage of total [functional](javascript:void(0);) [annotation](javascript:void(0);) genes.

**Top ten GO terms of transgressively up- regulated genes in L.**CCAA

| **Term^a^** | **Count** | **%^b^** | **P-Value** |
| --- | --- | --- | --- |
| Cluster 1; Enrichment Score: 7.58 | | | |
| GO:0016143~S-glycoside metabolic process | 14 | 2.33 | 3.43E-11 |
| GO:0019757~glycosinolate metabolic process | 14 | 2.33 | 3.43E-11 |
| GO:0019760~glucosinolate metabolic process | 14 | 2.33 | 3.43E-11 |
| GO:0019748~secondary metabolic process | 36 | 6.00 | 6.18E-10 |
| GO:0019758~glycosinolate biosynthetic process | 11 | 1.83 | 1.20E-09 |
| GO:0019761~glucosinolate biosynthetic process | 11 | 1.83 | 1.20E-09 |
| GO:0016144~S-glycoside biosynthetic process | 11 | 1.83 | 1.20E-09 |
| GO:0006790~sulfur metabolic process | 22 | 3.67 | 2.05E-09 |
| GO:0044272~sulfur compound biosynthetic process | 17 | 2.83 | 1.03E-08 |
| GO:0016137~glycoside metabolic process | 15 | 2.50 | 2.09E-08 |
| GO:0016138~glycoside biosynthetic process | 11 | 1.83 | 3.07E-06 |
| ath00966:Glucosinolate biosynthesis | 7 | 1.17 | 1.31E-05 |
| GO:0034637~cellular carbohydrate biosynthetic process | 13 | 2.17 | 0.003078 |
|  |  |  |  |
| Cluster 2; Enrichment Score: 3.70 | | | |
| GO:0005875~microtubule associated complex | 10 | 1.67 | 2.95E-06 |
| GO:0015630~microtubule cytoskeleton | 15 | 2.50 | 1.58E-05 |
| GO:0007018~microtubule-based movement | 11 | 1.83 | 1.99E-05 |
| GO:0003777~microtubule motor activity | 10 | 1.67 | 3.85E-05 |
| GO:0005874~microtubule | 12 | 2.00 | 7.84E-05 |
| GO:0044430~cytoskeletal part | 15 | 2.50 | 1.11E-04 |
| SM00129:KISc | 8 | 1.33 | 1.29E-04 |
| GO:0005856~cytoskeleton | 17 | 2.83 | 1.30E-04 |
| IPR019821:Kinesin, motor region, conserved site | 8 | 1.33 | 1.54E-04 |
| GO:0007017~microtubule-based process | 12 | 2.00 | 2.41E-04 |
| IPR001752:Kinesin, motor region | 8 | 1.33 | 4.09E-04 |
| GO:0003774~motor activity | 10 | 1.67 | 6.22E-04 |
| microtubule | 9 | 1.50 | 9.92E-04 |
| GO:0043232~intracellular non-membrane-bounded organelle | 43 | 7.17 | 0.001141 |
| GO:0043228~non-membrane-bounded organelle | 43 | 7.17 | 0.001141 |
| motor protein | 8 | 1.33 | 0.001474 |
|  |  |  |  |
| Cluster 3; Enrichment Score: 2.97 | | | |
| repeat:PPR 10 | 23 | 3.83 | 6.70E-05 |
| repeat:PPR 9 | 23 | 3.83 | 3.23E-04 |
| repeat:PPR 8 | 24 | 4.00 | 3.26E-04 |
| region of interest:Type E motif | 14 | 2.33 | 3.50E-04 |
| repeat:PPR 5 | 25 | 4.17 | 3.60E-04 |
| repeat:PPR 4 | 25 | 4.17 | 4.26E-04 |
| IPR002885:Pentatricopeptide repeat | 25 | 4.17 | 4.61E-04 |
| repeat:PPR 3 | 25 | 4.17 | 4.69E-04 |
| repeat:PPR 7 | 24 | 4.00 | 5.12E-04 |
| repeat:PPR 2 | 25 | 4.17 | 5.54E-04 |
| repeat:PPR 1 | 25 | 4.17 | 5.54E-04 |
| repeat:PPR 6 | 24 | 4.00 | 7.58E-04 |
| region of interest:Type E(+) motif | 12 | 2.00 | 0.001067 |
| repeat:PPR 11 | 17 | 2.83 | 0.002892 |
| repeat:PPR 12 | 14 | 2.33 | 0.014788 |
|  |  |  |  |
| Cluster 4; Enrichment Score: 2.21 | | | |
| GO:0009813~flavonoid biosynthetic process | 8 | 1.33 | 3.47E-04 |
| GO:0019438~aromatic compound biosynthetic process | 16 | 2.67 | 3.70E-04 |
| GO:0009812~flavonoid metabolic process | 8 | 1.33 | 5.46E-04 |
| GO:0009698~phenylpropanoid metabolic process | 13 | 2.17 | 0.001032 |
| GO:0009699~phenylpropanoid biosynthetic process | 11 | 1.83 | 0.001491 |
| GO:0042398~cellular amino acid derivative biosynthetic process | 12 | 2.00 | 0.010271 |
| GO:0006575~cellular amino acid derivative metabolic process | 15 | 2.50 | 0.011759 |
| flavonoid biosynthesis | 3 | 0.50 | 0.036473 |
|  |  |  |  |
| Cluster 5; Enrichment Score: 2.21 | | | |
| GO:0009267~cellular response to starvation | 8 | 1.33 | 8.27E-04 |
| GO:0031668~cellular response to extracellular stimulus | 9 | 1.50 | 9.79E-04 |
| GO:0042594~response to starvation | 8 | 1.33 | 0.00145 |
| GO:0031669~cellular response to nutrient levels | 8 | 1.33 | 0.001723 |
| GO:0016036~cellular response to phosphate starvation | 6 | 1.00 | 0.002226 |
| GO:0009991~response to extracellular stimulus | 9 | 1.50 | 0.002389 |
| GO:0031667~response to nutrient levels | 8 | 1.33 | 0.004298 |
| GO:0009247~glycolipid biosynthetic process | 4 | 0.67 | 0.008315 |
| GO:0006664~glycolipid metabolic process | 4 | 0.67 | 0.015099 |
| GO:0033554~cellular response to stress | 19 | 3.17 | 0.022793 |
| GO:0046467~membrane lipid biosynthetic process | 4 | 0.67 | 0.024252 |
|  |  |  |  |
| Cluster 6; Enrichment Score: 2.12 | | | |
| GO:0007049~cell cycle | 19 | 3.17 | 2.76E-05 |
| GO:0051301~cell division | 15 | 2.50 | 1.04E-04 |
| cell division | 9 | 1.50 | 0.001917 |
| cell cycle | 9 | 1.50 | 0.004009 |
| IPR013763:Cyclin-related | 6 | 1.00 | 0.005117 |
| SM00385:CYCLIN | 6 | 1.00 | 0.005925 |
| cyclin | 6 | 1.00 | 0.005931 |
| IPR004367:Cyclin, C-terminal | 5 | 0.83 | 0.006557 |
| IPR006671:Cyclin, N-terminal | 6 | 1.00 | 0.006707 |
| GO:0051726~regulation of cell cycle | 9 | 1.50 | 0.007796 |
| GO:0016538~cyclin-dependent protein kinase regulator activity | 5 | 0.83 | 0.009576 |
| IPR006670:Cyclin | 6 | 1.00 | 0.012573 |
| IPR014400:Cyclin, A/B/D/E | 4 | 0.67 | 0.013881 |
| cell cycle control | 3 | 0.50 | 0.015505 |
| PIRSF001771:Cyclin_A_B_D_E | 4 | 0.67 | 0.016823 |
| GO:0019887~protein kinase regulator activity | 5 | 0.83 | 0.019813 |
| GO:0019207~kinase regulator activity | 5 | 0.83 | 0.023126 |
|  |  |  |  |
| Cluster 7;Enrichment Score: 2.09 | | | |
| GO:0000910~cytokinesis | 6 | 1.00 | 0.003525 |
| GO:0000911~cytokinesis by cell plate formation | 4 | 0.67 | 0.009794 |
| GO:0033205~cytokinesis during cell cycle | 4 | 0.67 | 0.015099 |
|  |  |  |  |
| Cluster 8; Enrichment Score: 1.54 | | | |
| atp-binding | 55 | 9.17 | 0.004544 |
| nucleotide-binding | 58 | 9.67 | 0.013512 |
| GO:0030554~adenyl nucleotide binding | 79 | 13.17 | 0.02064 |
| GO:0001883~purine nucleoside binding | 79 | 13.17 | 0.02064 |
| GO:0001882~nucleoside binding | 79 | 13.17 | 0.022111 |
| nucleotide phosphate-binding region:ATP | 30 | 5.00 | 0.029366 |
| GO:0032559~adenyl ribonucleotide binding | 73 | 12.17 | 0.034922 |
| GO:0005524~ATP binding | 72 | 12.00 | 0.037623 |
| GO:0017076~purine nucleotide binding | 83 | 13.83 | 0.04805 |
|  |  |  |  |
| Cluster 9; Enrichment Score: 1.44 | | | |
| GO:0008017~microtubule binding | 5 | 0.83 | 0.006114 |
| GO:0015631~tubulin binding | 5 | 0.83 | 0.01411 |
|  |  |  |  |
| Cluster 10; Enrichment Score: 1.37 | | | |
| GO:0009851~auxin biosynthetic process | 4 | 0.67 | 0.015099 |
| GO:0042446~hormone biosynthetic process | 5 | 0.83 | 0.017786 |
| ath00380:Tryptophan metabolism | 5 | 0.83 | 0.022493 |
| GO:0010817~regulation of hormone levels | 8 | 1.33 | 0.031014 |

^a^ GO terms with enrichment score>0.5 and P<0.05 were considered to be significantly enriched.

^b^ Percentage of total [functional](javascript:void(0);) [annotation](javascript:void(0);) genes.

**Top ten GO terms of transgressively down- regulated genes in L.**CCAA

| **Term^a^** | **Count** | **%^b^** | **P-Value** |
| --- | --- | --- | --- |
| Cluster 1; Enrichment Score: 4.64 | | | |
| membrane | 98 | 15.46 | 2.62E-08 |
| transmembrane | 78 | 12.30 | 8.21E-06 |
| topological domain:Extracellular | 30 | 4.73 | 1.78E-05 |
| topological domain:Cytoplasmic | 38 | 5.99 | 2.42E-05 |
| transmembrane region | 74 | 11.67 | 1.57E-04 |
| GO:0031224~intrinsic to membrane | 96 | 15.14 | 3.52E-04 |
| GO:0016021~integral to membrane | 81 | 12.78 | 6.49E-04 |
|  |  |  |  |
| Cluster 2; Enrichment Score: 3.22 | | | |
| kinase | 48 | 7.57 | 2.70E-06 |
| topological domain:Extracellular | 30 | 4.73 | 1.78E-05 |
| topological domain:Cytoplasmic | 38 | 5.99 | 2.42E-05 |
| nucleotide-binding | 73 | 11.51 | 7.58E-05 |
| GO:0006468~protein amino acid phosphorylation | 51 | 8.04 | 9.44E-05 |
| active site:Proton acceptor | 33 | 5.21 | 1.06E-04 |
| IPR000719:Protein kinase, core | 47 | 7.41 | 1.09E-04 |
| GO:0006796~phosphate metabolic process | 58 | 9.15 | 1.10E-04 |
| GO:0006793~phosphorus metabolic process | 58 | 9.15 | 1.11E-04 |
| GO:0004674~protein serine/threonine kinase activity | 49 | 7.73 | 1.16E-04 |
| serine/threonine-protein kinase | 34 | 5.36 | 1.46E-04 |
| IPR017442:Serine/threonine protein kinase-related | 41 | 6.47 | 1.71E-04 |
| atp-binding | 65 | 10.25 | 1.83E-04 |
| IPR008271:Serine/threonine protein kinase, active site | 39 | 6.15 | 1.99E-04 |
| GO:0004672~protein kinase activity | 52 | 8.20 | 3.76E-04 |
| GO:0016310~phosphorylation | 52 | 8.20 | 5.70E-04 |
| GO:0004713~protein tyrosine kinase activity | 21 | 3.31 | 5.74E-04 |
| domain:Protein kinase | 26 | 4.10 | 6.40E-04 |
| binding site:ATP | 27 | 4.26 | 6.45E-04 |
| receptor | 22 | 3.47 | 9.03E-04 |
| GO:0001882~nucleoside binding | 97 | 15.30 | 0.001182 |
| GO:0001883~purine nucleoside binding | 96 | 15.14 | 0.001558 |
| GO:0030554~adenyl nucleotide binding | 96 | 15.14 | 0.001558 |
| IPR017441:Protein kinase, ATP binding site | 33 | 5.21 | 0.003036 |
| GO:0017076~purine nucleotide binding | 101 | 15.93 | 0.004882 |
| transferase | 52 | 8.20 | 0.006162 |
| GO:0032559~adenyl ribonucleotide binding | 87 | 13.72 | 0.007015 |
| GO:0005524~ATP binding | 84 | 13.25 | 0.01434 |
| GO:0032553~ribonucleotide binding | 92 | 14.51 | 0.017805 |
| GO:0032555~purine ribonucleotide binding | 92 | 14.51 | 0.017805 |
| nucleotide phosphate-binding region:ATP | 33 | 5.21 | 0.025345 |
|  |  |  |  |
| Cluster 3; Enrichment Score: 2.28 | | | |
| GO:0010033~response to organic substance | 55 | 8.68 | 8.92E-05 |
| GO:0007242~intracellular signaling cascade | 40 | 6.31 | 2.19E-04 |
| GO:0009719~response to endogenous stimulus | 46 | 7.26 | 3.21E-04 |
| GO:0009725~response to hormone stimulus | 43 | 6.78 | 5.02E-04 |
| GO:0009755~hormone-mediated signaling | 25 | 3.94 | 7.48E-04 |
| GO:0032870~cellular response to hormone stimulus | 25 | 3.94 | 7.48E-04 |
| GO:0000160~two-component signal transduction system (phosphorelay) | 16 | 2.52 | 9.24E-04 |
| Ethylene signaling pathway | 9 | 1.42 | 0.030549 |
| SM00380:AP2 | 9 | 1.42 | 0.030558 |
| GO:0009873~ethylene mediated signaling pathway | 10 | 1.58 | 0.042467 |
| IPR001471:Pathogenesis-related transcriptional factor and ERF, DNA-binding | 9 | 1.42 | 0.042869 |
|  |  |  |  |
| Cluster 4; Enrichment Score: 2.09 | | | |
| glycoprotein | 48 | 7.57 | 2.17E-05 |
| signal | 57 | 8.99 | 6.14E-04 |
| glycosylation site:N-linked (GlcNAc...) | 45 | 7.10 | 0.003082 |
| signal peptide | 57 | 8.99 | 0.029276 |
| GO:0005576~extracellular region | 34 | 5.36 | 0.431304 |
|  |  |  |  |
| Cluster 5; Enrichment Score: 2.00 | | | |
| PIRSF500186:response regulator, plant A-type | 4 | 0.63 | 0.003264 |
| SM00448:REC | 6 | 0.95 | 0.003699 |
| GO:0007623~circadian rhythm | 6 | 0.95 | 0.003869 |
| PIRSF002866:signal transduction receiver (phosphoacceptor) protein, CheY type | 4 | 0.63 | 0.004383 |
| IPR001789:Signal transduction response regulator, receiver region | 6 | 0.95 | 0.004919 |
| GO:0000156~two-component response regulator activity | 6 | 0.95 | 0.006307 |
| GO:0048511~rhythmic process | 6 | 0.95 | 0.010284 |
| domain:Response regulatory | 5 | 0.79 | 0.01625 |
| two-component regulatory system | 5 | 0.79 | 0.016726 |
| GO:0009735~response to cytokinin stimulus | 7 | 1.10 | 0.021471 |
| GO:0009736~cytokinin mediated signaling | 5 | 0.79 | 0.043483 |
|  |  |  |  |
| Cluster 6; Enrichment Score: 1.56 | | | |
| phosphotransferase | 10 | 1.58 | 0.004075 |
| serine/threonine-specific protein kinase | 4 | 0.63 | 0.048571 |
|  |  |  |  |
| Cluster 7; Enrichment Score: 1.49 | | | |
| GO:0000041~transition metal ion transport | 8 | 1.26 | 0.003596 |
|  |  |  |  |
| Cluster 8; Enrichment Score: 1.40 | | | |
| IPR010402:CCT domain | 5 | 0.79 | 0.02118 |
| domain:CCT | 4 | 0.63 | 0.038457 |
|  |  |  |  |
| Cluster 9; Enrichment Score: 1.39 | | | |
| Term | Count | % | PValue |
| GO:0006811~ion transport | 27 | 4.26 | 9.69E-04 |
| GO:0000041~transition metal ion transport | 8 | 1.26 | 0.003596 |
| GO:0030001~metal ion transport | 16 | 2.52 | 0.003802 |
|  |  |  |  |
| Cluster 10; Enrichment Score: 1.35 | | | |
| PIRSF017893:PIRSF017893 | 3 | 0.47 | 0.009517 |

^a^ GO terms with enrichment score>0.5 and P<0.05 were considered to be significantly enriched.

^b^ Percentage of total [functional](javascript:void(0);) [annotation](javascript:void(0);) genes.

**Top ten GO terms of transgressively up-regulated genes in L.AACC**

| **Term^a^** | **Count** | **%^b^** | **P-Value** |
| --- | --- | --- | --- |
| Cluster 1; Enrichment Score: 2.80 | | | |
| GO:0010033~response to organic substance | 53 | 8.94 | 6.36E-06 |
| GO:0009719~response to endogenous stimulus | 45 | 7.59 | 2.19E-05 |
| GO:0009725~response to hormone stimulus | 39 | 6.58 | 3.97E-04 |
| GO:0009737~response to abscisic acid stimulus | 17 | 2.87 | 0.001185 |
| GO:0009755~hormone-mediated signaling | 16 | 2.70 | 0.087151 |
|  |  |  |  |
| Cluster 2; Enrichment Score: 2.67 | | | |
| GO:0070469~respiratory chain | 11 | 1.85 | 4.49E-05 |
| GO:0045271~respiratory chain complex I | 5 | 0.84 | 0.01472 |
| GO:0030964~NADH dehydrogenase complex | 5 | 0.84 | 0.01472 |
|  |  |  |  |
| Cluster 3; Enrichment Score: 1.90 | | | |
| GO:0044429~mitochondrial part | 18 | 3.04 | 3.37E-04 |
| GO:0031966~mitochondrial membrane | 14 | 2.36 | 0.001096 |
| GO:0005740~mitochondrial envelope | 14 | 2.36 | 0.001876 |
| GO:0044455~mitochondrial membrane part | 7 | 1.18 | 0.0057 |
| GO:0005743~mitochondrial inner membrane | 11 | 1.85 | 0.006474 |
| GO:0005746~mitochondrial respiratory chain | 4 | 0.67 | 0.022713 |
| GO:0031967~organelle envelope | 25 | 4.22 | 0.026407 |
| GO:0031975~envelope | 25 | 4.22 | 0.02837 |
| GO:0019866~organelle inner membrane | 11 | 1.85 | 0.029463 |
| GO:0031090~organelle membrane | 27 | 4.55 | 0.03678 |
| GO:0005750~mitochondrial respiratory chain complex III | 3 | 0.51 | 0.049069 |
| GO:0045275~respiratory chain complex III | 3 | 0.51 | 0.049069 |
|  |  |  |  |
| Cluster 4; Enrichment Score: 1.79 | | | |
| GO:0009628~response to abiotic stimulus | 43 | 7.25 | 0.005605 |
| GO:0006970~response to osmotic stress | 17 | 2.87 | 0.026554 |
| GO:0009651~response to salt stress | 16 | 2.70 | 0.028019 |
|  |  |  |  |
| Cluster 5; Enrichment Score: 1.40 | | | |
| GO:0016053~organic acid biosynthetic process | 18 | 3.04 | 0.016444 |
| GO:0046394~carboxylic acid biosynthetic process | 18 | 3.04 | 0.016444 |
| amino-acid biosynthesis | 7 | 1.18 | 0.029403 |
| GO:0008652~cellular amino acid biosynthetic process | 10 | 1.69 | 0.030062 |
| GO:0044271~nitrogen compound biosynthetic process | 20 | 3.37 | 0.030256 |
| GO:0009309~amine biosynthetic process | 10 | 1.69 | 0.0521 |
|  |  |  |  |
| Cluster 6; Enrichment Score: 1.31 | | | |
| IPR000449:Ubiquitin-associated/translation elongation factor EF1B, N-terminal | 5 | 0.84 | 0.007248 |
|  |  |  |  |
| Cluster 7; Enrichment Score: 1.22 | | | |
| IPR002885:Pentatricopeptide repeat | 21 | 3.54 | 0.007001 |
| repeat:PPR 5 | 19 | 3.20 | 0.012823 |
| repeat:PPR 8 | 18 | 3.04 | 0.013758 |
| region of interest:Type E motif; degenerate | 4 | 0.67 | 0.014209 |
| repeat:PPR 4 | 19 | 3.20 | 0.014292 |
| repeat:PPR 3 | 19 | 3.20 | 0.015235 |
| repeat:PPR 1 | 19 | 3.20 | 0.016914 |
| repeat:PPR 2 | 19 | 3.20 | 0.016914 |
| repeat:PPR 7 | 18 | 3.04 | 0.018273 |
| repeat:PPR 6 | 18 | 3.04 | 0.023388 |
| repeat:PPR 9 | 16 | 2.70 | 0.031691 |
| region of interest:Type E motif | 9 | 1.52 | 0.039611 |
|  |  |  |  |
| Cluster 8; Enrichment Score: 1.14 | | | |
| GO:0019757~glycosinolate metabolic process | 5 | 0.84 | 0.019439 |
| GO:0019760~glucosinolate metabolic process | 5 | 0.84 | 0.019439 |
| GO:0016143~S-glycoside metabolic process | 5 | 0.84 | 0.019439 |
| GO:0019761~glucosinolate biosynthetic process | 4 | 0.67 | 0.029936 |
| GO:0019758~glycosinolate biosynthetic process | 4 | 0.67 | 0.029936 |
| GO:0016144~S-glycoside biosynthetic process | 4 | 0.67 | 0.029936 |
|  |  |  |  |
| Cluster 9; Enrichment Score: 1.12 | | | |
| GO:0009751~response to salicylic acid stimulus | 13 | 2.19 | 2.21E-04 |
| IPR006447:Myb-like DNA-binding region, SHAQKYF class | 6 | 1.01 | 0.048283 |
|  |  |  |  |
| Cluster 10; Enrichment Score: 1.087 | | | |
| GO:0009744~response to sucrose stimulus | 4 | 0.67 | 0.051931 |

^a^ GO terms with enrichment score>0.5 and P<0.05 were considered to be significantly enriched.

^b^ Percentage of total [functional](javascript:void(0);) [annotation](javascript:void(0);) genes.

**Top ten GO terms of transgressively down-regulated genes** in L.AACC

| **Term^a^** | **Count** | **%^b^** | **P-Value** |
| --- | --- | --- | --- |
| Cluster 1; Enrichment Score: 2.88 | | | |
| GO:0048364~root development | 20 | 2.18 | 4.71E-04 |
| GO:0022622~root system development | 20 | 2.18 | 4.71E-04 |
| GO:0010015~root morphogenesis | 10 | 1.09 | 0.010103 |
|  |  |  |  |
| Cluster 2; Enrichment Score: 2.41 | | | |
| GO:0003002~regionalization | 12 | 1.31 | 0.001245 |
| GO:0007389~pattern specification process | 13 | 1.41 | 0.002023 |
| GO:0010051~xylem and phloem pattern formation | 6 | 0.65 | 0.02324 |
|  |  |  |  |
| Cluster 3; Enrichment Score: 2.32 | | | |
| GO:0005618~cell wall | 38 | 4.13 | 0.003411 |
| GO:0030312~external encapsulating structure | 38 | 4.13 | 0.004364 |
| GO:0009505~plant-type cell wall | 20 | 2.18 | 0.007391 |
|  |  |  |  |
| Cluster 4; Enrichment Score: 2.25 | | | |
| GO:0051301~cell division | 17 | 1.85 | 0.001752 |
| GO:0007049~cell cycle | 20 | 2.18 | 0.003754 |
| cell cycle | 11 | 1.20 | 0.006109 |
| cell division | 9 | 0.98 | 0.02381 |
|  |  |  |  |
| Cluster 5; Enrichment Score: 2.20 | | | |
| GO:0043233~organelle lumen | 41 | 4.46 | 0.00228 |
| GO:0070013~intracellular organelle lumen | 41 | 4.46 | 0.00228 |
| GO:0031974~membrane-enclosed lumen | 41 | 4.46 | 0.002848 |
| GO:0031981~nuclear lumen | 28 | 3.05 | 0.022685 |
| GO:0005730~nucleolus | 21 | 2.29 | 0.028745 |
|  |  |  |  |
| Cluster 6; Enrichment Score: 2.16 | | | |
| signal recognition particle | 5 | 0.54 | 8.21E-05 |
| GO:0005786~signal recognition particle, endoplasmic reticulum targeting | 5 | 0.54 | 7.96E-04 |
| GO:0048500~signal recognition particle | 5 | 0.54 | 0.001516 |
| GO:0006605~protein targeting | 13 | 1.41 | 0.007779 |
| GO:0006612~protein targeting to membrane | 6 | 0.65 | 0.008111 |
| GO:0008312~7S RNA binding | 4 | 0.44 | 0.009878 |
| GO:0006614~SRP-dependent cotranslational protein targeting to membrane | 4 | 0.44 | 0.011546 |
| GO:0006613~cotranslational protein targeting to membrane | 4 | 0.44 | 0.011546 |
| GO:0045047~protein targeting to ER | 4 | 0.44 | 0.014558 |
|  |  |  |  |
| Cluster 7; Enrichment Score: 2.05 | | | |
| gtp-binding | 17 | 1.85 | 0.001745 |
| nucleotide phosphate-binding region:GTP | 11 | 1.20 | 0.00506 |
| GO:0005525~GTP binding | 21 | 2.29 | 0.009159 |
| GO:0032561~guanyl ribonucleotide binding | 22 | 2.39 | 0.010045 |
| GO:0019001~guanyl nucleotide binding | 22 | 2.39 | 0.011195 |
|  |  |  |  |
| Cluster 8; Enrichment Score: 1.80 | | | |
| GO:0031090~organelle membrane | 51 | 5.55 | 0.001312 |
| GO:0031976~plastid thylakoid | 26 | 2.83 | 0.001674 |
| GO:0009534~chloroplast thylakoid | 26 | 2.83 | 0.001674 |
| GO:0031984~organelle subcompartment | 26 | 2.83 | 0.001813 |
| GO:0009579~thylakoid | 33 | 3.59 | 0.002101 |
| GO:0055035~plastid thylakoid membrane | 21 | 2.29 | 0.007529 |
| GO:0009535~chloroplast thylakoid membrane | 21 | 2.29 | 0.007529 |
| GO:0044436~thylakoid part | 24 | 2.61 | 0.008725 |
| GO:0042651~thylakoid membrane | 21 | 2.29 | 0.01271 |
| GO:0034357~photosynthetic membrane | 22 | 2.39 | 0.014792 |
| chloroplast | 37 | 4.03 | 0.038872 |
| GO:0044434~chloroplast part | 48 | 5.22 | 0.039233 |
| transit peptide | 44 | 4.79 | 0.048176 |
|  |  |  |  |
| Cluster 9; Enrichment Score: 1.80 | | | |
| oxidoreductase | 54 | 5.88 | 1.80E-04 |
| iron | 36 | 3.92 | 0.003214 |
| GO:0005506~iron ion binding | 42 | 4.57 | 0.040074 |
|  |  |  |  |
| Cluster 10; Enrichment Score: 1.79 | | | |
| metalloprotein | 14 | 1.52 | 0.002728 |
| iron | 36 | 3.92 | 0.003214 |

^a^ GO terms with enrichment score>0.5 and P<0.05 were considered to be significantly enriched.

^b^ Percentage of total [functional](javascript:void(0);) [annotation](javascript:void(0);) genes.

**Top ten GO terms of transgressively up-regulated genes in S.AABB**

| **Term^a^** | **Count** | **%^b^** | **P-Value** |
| --- | --- | --- | --- |
| Cluster 1; Enrichment Score: 22.11 | | | |
| GO:0044434~chloroplast part | 131 | 12.83 | 6.35E-34 |
| GO:0044435~plastid part | 131 | 12.83 | 1.58E-32 |
| GO:0009534~chloroplast thylakoid | 75 | 7.35 | 1.31E-31 |
| GO:0031976~plastid thylakoid | 75 | 7.35 | 1.31E-31 |
| GO:0031984~organelle subcompartment | 75 | 7.35 | 1.95E-31 |
| GO:0044436~thylakoid part | 75 | 7.35 | 5.21E-31 |
| GO:0009579~thylakoid | 88 | 8.62 | 8.87E-31 |
| chloroplast | 104 | 10.19 | 5.18E-29 |
| GO:0055035~plastid thylakoid membrane | 64 | 6.27 | 1.84E-27 |
| GO:0009535~chloroplast thylakoid membrane | 64 | 6.27 | 1.84E-27 |
| GO:0042651~thylakoid membrane | 65 | 6.37 | 5.51E-27 |
| transit peptide:Chloroplast | 93 | 9.11 | 3.39E-26 |
| GO:0034357~photosynthetic membrane | 66 | 6.46 | 9.36E-26 |
| GO:0009536~plastid | 249 | 24.39 | 6.35E-24 |
| GO:0015979~photosynthesis | 47 | 4.60 | 8.20E-24 |
| plastid | 93 | 9.11 | 1.83E-23 |
| GO:0009507~chloroplast | 244 | 23.90 | 2.48E-23 |
| transit peptide | 103 | 10.09 | 8.20E-21 |
| thylakoid | 38 | 3.72 | 8.51E-19 |
| GO:0009532~plastid stroma | 62 | 6.07 | 4.09E-16 |
| GO:0009570~chloroplast stroma | 60 | 5.88 | 5.25E-16 |
| GO:0031090~organelle membrane | 84 | 8.23 | 1.02E-12 |
| GO:0009526~plastid envelope | 52 | 5.09 | 5.10E-10 |
| GO:0009941~chloroplast envelope | 50 | 4.90 | 9.11E-10 |
| GO:0031967~organelle envelope | 55 | 5.39 | 1.14E-04 |
| GO:0031975~envelope | 55 | 5.39 | 1.38E-04 |
|  |  |  |  |
| Cluster 2; Enrichment Score: 17.34 | | | |
| GO:0015979~photosynthesis | 47 | 4.60 | 8.20E-24 |
| GO:0019684~photosynthesis, light reaction | 31 | 3.04 | 5.54E-20 |
| GO:0006091~generation of precursor metabolites and energy | 49 | 4.80 | 2.06E-10 |
|  |  |  |  |
| Cluster 3; Enrichment Score: 10.64 | | | |
| thylakoid | 38 | 3.72 | 8.51E-19 |
| photosynthesis | 21 | 2.06 | 7.89E-11 |
| GO:0009521~photosystem | 20 | 1.96 | 1.16E-10 |
| ath00195:Photosynthesis | 15 | 1.47 | 3.68E-05 |
|  |  |  |  |
| Cluster 4; Enrichment Score: 8.30 | | | |
| thylakoid | 38 | 3.72 | 8.51E-19 |
| GO:0009543~chloroplast thylakoid lumen | 23 | 2.25 | 6.62E-13 |
| GO:0031978~plastid thylakoid lumen | 23 | 2.25 | 6.62E-13 |
| GO:0031977~thylakoid lumen | 24 | 2.35 | 2.61E-12 |
| transit peptide:Thylakoid | 19 | 1.86 | 1.04E-11 |
|  |  |  |  |
| Cluster 5; Enrichment Score: 7.11 | | | |
| GO:0019684~photosynthesis, light reaction | 31 | 3.04 | 5.54E-20 |
| IPR001344:Chlorophyll A-B binding protein | 13 | 1.27 | 1.86E-11 |
| ath00196:Photosynthesis | 13 | 1.27 | 2.41E-11 |
| GO:0009765~photosynthesis, light harvesting | 14 | 1.37 | 2.80E-10 |
| GO:0016168~chlorophyll binding | 12 | 1.18 | 1.54E-08 |
| GO:0030076~light-harvesting complex | 11 | 1.08 | 1.31E-07 |
| GO:0010287~plastoglobule | 14 | 1.37 | 1.22E-06 |
| PIRSF002925:chlorophyll a/b-binding protein | 7 | 0.69 | 1.09E-05 |
| GO:0009637~response to blue light | 12 | 1.18 | 1.98E-05 |
| chlorophyll | 6 | 0.59 | 1.67E-04 |
| GO:0010218~response to far red light | 9 | 0.88 | 5.36E-04 |
| GO:0010114~response to red light | 10 | 0.98 | 6.31E-04 |
| GO:0009639~response to red or far red light | 15 | 1.47 | 0.016278 |
|  |  |  |  |
| Cluster 6; Enrichment Score: 7.07 | | | |
| GO:0009767~photosynthetic electron transport chain | 13 | 1.27 | 1.07E-09 |
| GO:0009773~photosynthetic electron transport in photosystem I | 9 | 0.88 | 2.88E-08 |
| GO:0022900~electron transport chain | 22 | 2.15 | 1.98E-05 |
|  |  |  |  |
| Cluster 7; Enrichment Score: 4.85 | | | |
| GO:0016051~carbohydrate biosynthetic process | 32 | 3.13 | 1.95E-07 |
| GO:0034637~cellular carbohydrate biosynthetic process | 24 | 2.35 | 7.74E-06 |
| GO:0016138~glycoside biosynthetic process | 13 | 1.27 | 1.19E-05 |
| GO:0016137~glycoside metabolic process | 15 | 1.47 | 1.40E-05 |
| GO:0019757~glycosinolate metabolic process | 11 | 1.08 | 1.44E-05 |
| GO:0019760~glucosinolate metabolic process | 11 | 1.08 | 1.44E-05 |
| GO:0016143~S-glycoside metabolic process | 11 | 1.08 | 1.44E-05 |
| GO:0044272~sulfur compound biosynthetic process | 17 | 1.67 | 1.45E-05 |
| GO:0006790~sulfur metabolic process | 22 | 2.15 | 1.66E-05 |
| GO:0019761~glucosinolate biosynthetic process | 9 | 0.88 | 2.31E-05 |
| GO:0019758~glycosinolate biosynthetic process | 9 | 0.88 | 2.31E-05 |
| GO:0016144~S-glycoside biosynthetic process | 9 | 0.88 | 2.31E-05 |
| ath00966:Glucosinolate biosynthesis | 7 | 0.69 | 4.32E-04 |
|  |  |  |  |
| Cluster 8; Enrichment Score: 4.38 | | | |
| GO:0019253~reductive pentose-phosphate cycle | 7 | 0.69 | 1.46E-05 |
| GO:0019685~photosynthesis, dark reaction | 7 | 0.69 | 2.34E-05 |
| calvin cycle | 6 | 0.59 | 4.84E-05 |
| ath00710:Carbon fixation in photosynthetic organisms | 15 | 1.47 | 5.06E-05 |
| GO:0015977~carbon utilization by fixation of carbon dioxide | 7 | 0.69 | 1.51E-04 |
|  |  |  |  |
| Cluster 9; Enrichment Score: 3.88 | | | |
| IPR001344:Chlorophyll A-B binding protein | 13 | 1.27 | 1.86E-11 |
| ath00196:Photosynthesis | 13 | 1.27 | 2.41E-11 |
| GO:0009522~photosystem I | 12 | 1.18 | 1.46E-08 |
| GO:0016168~chlorophyll binding | 12 | 1.18 | 1.54E-08 |
| Photosystem I | 9 | 0.88 | 1.74E-06 |
| photosystem ii | 10 | 0.98 | 7.27E-06 |
| GO:0009523~photosystem II | 12 | 1.18 | 1.03E-05 |
| PIRSF002925:chlorophyll a/b-binding protein | 7 | 0.69 | 1.09E-05 |
| chlorophyll | 6 | 0.59 | 1.67E-04 |
| metal ion-binding site:Magnesium (chlorophyll-a 6 axial ligand) | 4 | 0.39 | 0.002689 |
| chromophore | 6 | 0.59 | 0.003082 |
| metal ion-binding site:Magnesium (chlorophyll-a 5 axial ligand) | 4 | 0.39 | 0.004161 |
| metal ion-binding site:Magnesium (chlorophyll-b 1 axial ligand); via carbonyl oxygen | 4 | 0.39 | 0.004161 |
| metal ion-binding site:Magnesium (chlorophyll-a 1 axial ligand) | 4 | 0.39 | 0.004161 |
| metal ion-binding site:Magnesium (chlorophyll-a 4 axial ligand) | 4 | 0.39 | 0.004161 |
| metal ion-binding site:Magnesium (chlorophyll-a 3 axial ligand) | 4 | 0.39 | 0.004161 |
| binding site:Chlorophyll-a 1; via amide nitrogen | 4 | 0.39 | 0.004161 |
| binding site:Chlorophyll-a 1 | 4 | 0.39 | 0.004161 |
| metal ion-binding site:Magnesium (chlorophyll-b 3 axial ligand) | 4 | 0.39 | 0.004161 |
| binding site:Chlorophyll-b 4 | 4 | 0.39 | 0.004161 |
| GO:0018298~protein-chromophore linkage | 6 | 0.59 | 0.005545 |
| metal ion-binding site:Magnesium (chlorophyll-b 2 axial ligand); via carbonyl oxygen | 3 | 0.29 | 0.018116 |
| binding site:Chlorophyll-b 2 | 3 | 0.29 | 0.018116 |
| binding site:Chlorophyll-a 5 | 3 | 0.29 | 0.018116 |
| metal ion-binding site:Magnesium (chlorophyll-a 2 axial ligand) | 3 | 0.29 | 0.035856 |
|  |  |  |  |
| Cluster 10; Enrichment Score: 3.70 | | | |
| GO:0042742~defense response to bacterium | 23 | 2.25 | 1.61E-05 |
| GO:0009617~response to bacterium | 26 | 2.55 | 6.15E-05 |
| GO:0006952~defense response | 62 | 6.07 | 0.007933 |

^a^ GO terms with enrichment score>0.5 and P<0.05 were considered to be significantly enriched.

^b^ Percentage of total [functional](javascript:void(0);) [annotation](javascript:void(0);) genes.

**Top ten GO terms of transgressively down- regulated genes in S.AABB**

| **Term^a^** | **Count** | **%^b^** | **P-Value** |
| --- | --- | --- | --- |
| Cluster 1; Enrichment Score: 8.88 | | | |
| GO:0005840~ribosome | 40 | 8.91 | 9.07E-14 |
| GO:0043228~non-membrane-bounded organelle | 64 | 14.25 | 2.84E-13 |
| GO:0043232~intracellular non-membrane-bounded organelle | 64 | 14.25 | 2.84E-13 |
| GO:0022626~cytosolic ribosome | 32 | 7.13 | 5.31E-13 |
| GO:0003735~structural constituent of ribosome | 34 | 7.57 | 1.95E-12 |
| GO:0033279~ribosomal subunit | 29 | 6.46 | 1.43E-11 |
| GO:0005829~cytosol | 45 | 10.02 | 4.32E-11 |
| GO:0044445~cytosolic part | 27 | 6.01 | 6.73E-11 |
| ribosomal protein | 28 | 6.24 | 7.51E-11 |
| GO:0030529~ribonucleoprotein complex | 43 | 9.58 | 1.01E-10 |
| ribonucleoprotein | 29 | 6.46 | 1.05E-10 |
| GO:0005198~structural molecule activity | 36 | 8.02 | 4.68E-10 |
| GO:0015934~large ribosomal subunit | 18 | 4.01 | 1.05E-07 |
| GO:0022625~cytosolic large ribosomal subunit | 14 | 3.12 | 5.45E-06 |
| GO:0022627~cytosolic small ribosomal subunit | 11 | 2.45 | 1.22E-04 |
| ath03010:Ribosome | 21 | 4.68 | 1.81E-04 |
| GO:0006412~translation | 44 | 9.80 | 2.53E-04 |
| GO:0015935~small ribosomal subunit | 11 | 2.45 | 4.29E-04 |
|  |  |  |  |
| Cluster 2; Enrichment Score: 7.26 | | | |
| GO:0043228~non-membrane-bounded organelle | 64 | 14.25 | 2.84E-13 |
| GO:0043232~intracellular non-membrane-bounded organelle | 64 | 14.25 | 2.84E-13 |
| GO:0005730~nucleolus | 26 | 5.79 | 2.37E-08 |
| GO:0031981~nuclear lumen | 28 | 6.24 | 1.66E-06 |
| GO:0031974~membrane-enclosed lumen | 31 | 6.90 | 4.92E-05 |
| GO:0043233~organelle lumen | 30 | 6.68 | 9.81E-05 |
| GO:0070013~intracellular organelle lumen | 30 | 6.68 | 9.81E-05 |
|  |  |  |  |
| Cluster 3; Enrichment Score: 5.54 | | | |
| GO:0006270~DNA replication initiation | 8 | 1.78 | 9.09E-10 |
| IPR018525:DNA-dependent ATPase MCM, conserved site | 6 | 1.34 | 1.38E-08 |
| GO:0006261~DNA-dependent DNA replication | 11 | 2.45 | 3.13E-08 |
| SM00350:MCM | 6 | 1.34 | 3.70E-08 |
| IPR001208:DNA-dependent ATPase MCM | 6 | 1.34 | 1.25E-07 |
| GO:0006260~DNA replication | 15 | 3.34 | 1.78E-07 |
| dna replication | 8 | 1.78 | 7.84E-07 |
| GO:0006268~DNA unwinding during replication | 6 | 1.34 | 2.26E-06 |
| ath03030:DNA replication | 10 | 2.23 | 5.39E-06 |
| IPR012340:Nucleic acid-binding, OB-fold | 11 | 2.45 | 1.02E-05 |
| GO:0032392~DNA geometric change | 6 | 1.34 | 1.17E-05 |
| GO:0032508~DNA duplex unwinding | 6 | 1.34 | 1.17E-05 |
| GO:0006259~DNA metabolic process | 21 | 4.68 | 2.87E-05 |
| GO:0008094~DNA-dependent ATPase activity | 7 | 1.56 | 8.90E-05 |
| GO:0042623~ATPase activity, coupled | 13 | 2.90 | 0.042569 |
| GO:0016887~ATPase activity | 16 | 3.56 | 0.043363 |
|  |  |  |  |
| Cluster 4; Enrichment Score: 5.29 | | | |
| GO:0010038~response to metal ion | 25 | 5.57 | 9.30E-07 |
| GO:0046686~response to cadmium ion | 21 | 4.68 | 1.07E-05 |
| GO:0010035~response to inorganic substance | 28 | 6.24 | 1.32E-05 |
|  |  |  |  |
| Cluster 5; Enrichment Score: 2.69 | | | |
| ath03010:Ribosome | 21 | 4.68 | 1.81E-04 |
| GO:0022613~ribonucleoprotein complex biogenesis | 13 | 2.90 | 0.004682 |
| GO:0042254~ribosome biogenesis | 12 | 2.67 | 0.010089 |
|  |  |  |  |
| Cluster 6; Enrichment Score: 2.05 | | | |
| GO:0019843~rRNA binding | 7 | 1.56 | 0.003784 |
| rrna-binding | 6 | 1.34 | 0.004823 |
| rna-binding | 10 | 2.23 | 0.037517 |
|  |  |  |  |
| Cluster 7; Enrichment Score: 1.80 | | | |
| IPR002015:Proteasome/cyclosome, regulatory subunit | 4 | 0.89 | 6.48E-05 |
| GO:0009894~regulation of catabolic process | 4 | 0.89 | 0.003729 |
| GO:0042176~regulation of protein catabolic process | 3 | 0.67 | 0.00575 |
| proteasome | 5 | 1.11 | 0.016694 |
| ath03050:Proteasome | 7 | 1.56 | 0.017826 |
| GO:0008540~proteasome regulatory particle, base subcomplex | 3 | 0.67 | 0.02438 |
| GO:0000502~proteasome complex | 5 | 1.11 | 0.035013 |
|  |  |  |  |
| Cluster 8; Enrichment Score: 1.68 | | | |
| GO:0006413~translational initiation | 7 | 1.56 | 0.003733 |
| GO:0006417~regulation of translation | 5 | 1.11 | 0.006946 |
| GO:0008135~translation factor activity, nucleic acid binding | 9 | 2.00 | 0.009277 |
| GO:0032268~regulation of cellular protein metabolic process | 5 | 1.11 | 0.014584 |
| GO:0003743~translation initiation factor activity | 7 | 1.56 | 0.015309 |
| GO:0043021~ribonucleoprotein binding | 3 | 0.67 | 0.018834 |
|  |  |  |  |
| Cluster 9; Enrichment Score: 1.51 | | | |
| GO:0044427~chromosomal part | 12 | 2.67 | 3.89E-04 |
| GO:0005694~chromosome | 13 | 2.90 | 0.001439 |
| GO:0032993~protein-DNA complex | 7 | 1.56 | 0.00316 |
| nucleosome core | 5 | 1.11 | 0.007079 |
| IPR007125:Histone core | 5 | 1.11 | 0.010908 |
| GO:0006333~chromatin assembly or disassembly | 7 | 1.56 | 0.011206 |
| GO:0031497~chromatin assembly | 6 | 1.34 | 0.014148 |
| GO:0065004~protein-DNA complex assembly | 6 | 1.34 | 0.014961 |
| chromosomal protein | 5 | 1.11 | 0.016694 |
| IPR009072:Histone-fold | 6 | 1.34 | 0.018551 |
| GO:0006323~DNA packaging | 6 | 1.34 | 0.019496 |
| GO:0000785~chromatin | 6 | 1.34 | 0.039836 |
| GO:0000786~nucleosome | 5 | 1.11 | 0.040798 |
| GO:0065003~macromolecular complex assembly | 11 | 2.45 | 0.047156 |
|  |  |  |  |
| Cluster 10; Enrichment Score: 1.47 | | | |
| er-golgi transport | 7 | 1.56 | 8.42E-05 |
| protein transport | 11 | 2.45 | 0.005049 |
| golgi apparatus | 10 | 2.23 | 0.005157 |
| GO:0005794~Golgi apparatus | 15 | 3.34 | 0.019323 |
| GO:0015031~protein transport | 17 | 3.79 | 0.038496 |
| GO:0045184~establishment of protein localization | 17 | 3.79 | 0.038496 |

^a^ GO terms with enrichment score>0.5 and P<0.05 were considered to be significantly enriched.

^b^ Percentage of total [functional](javascript:void(0);) [annotation](javascript:void(0);) genes.

**Top ten GO terms of transgressively up- regulated genes in S.**BBCC

| **Term^a^** | **Count** | **%^b^** | **P-Value** |
| --- | --- | --- | --- |
| Cluster 1; Enrichment Score: 10.65 | | | |
| topological domain:Extracellular | 58 | 6.88 | 9.85E-16 |
| topological domain:Cytoplasmic | 69 | 8.19 | 3.87E-14 |
| transmembrane | 122 | 14.47 | 3.85E-13 |
| membrane | 136 | 16.13 | 1.19E-12 |
| transmembrane region | 117 | 13.88 | 1.54E-09 |
| GO:0031224~intrinsic to membrane | 142 | 16.84 | 2.10E-07 |
| GO:0016021~integral to membrane | 121 | 14.35 | 4.93E-07 |
|  |  |  |  |
| Cluster 2; Enrichment Score: 9.17 | | | |
| kinase | 85 | 10.08 | 1.49E-17 |
| serine/threonine-protein kinase | 70 | 8.30 | 2.10E-17 |
| receptor | 51 | 6.05 | 3.27E-16 |
| topological domain:Extracellular | 58 | 6.88 | 9.85E-16 |
| IPR017442:Serine/threonine protein kinase-related | 77 | 9.13 | 2.37E-14 |
| topological domain:Cytoplasmic | 69 | 8.19 | 3.87E-14 |
| IPR017441:Protein kinase, ATP binding site | 70 | 8.30 | 5.87E-14 |
| IPR008271:Serine/threonine protein kinase, active site | 72 | 8.54 | 2.35E-13 |
| GO:0006468~protein amino acid phosphorylation | 93 | 11.03 | 3.80E-13 |
| IPR000719:Protein kinase, core | 82 | 9.73 | 8.82E-13 |
| transferase | 100 | 11.86 | 1.53E-12 |
| GO:0004674~protein serine/threonine kinase activity | 86 | 10.20 | 9.95E-12 |
| GO:0004672~protein kinase activity | 93 | 11.03 | 2.07E-11 |
| atp-binding | 105 | 12.46 | 4.42E-11 |
| GO:0016310~phosphorylation | 94 | 11.15 | 4.67E-11 |
| nucleotide-binding | 112 | 13.29 | 2.40E-10 |
| GO:0006796~phosphate metabolic process | 97 | 11.51 | 2.70E-10 |
| GO:0006793~phosphorus metabolic process | 97 | 11.51 | 2.82E-10 |
| binding site:ATP | 46 | 5.46 | 2.12E-08 |
| domain:Protein kinase | 44 | 5.22 | 3.21E-08 |
| active site:Proton acceptor | 48 | 5.69 | 8.12E-07 |
| nucleotide phosphate-binding region:ATP | 59 | 7.00 | 3.54E-06 |
| GO:0032559~adenyl ribonucleotide binding | 135 | 16.01 | 1.94E-05 |
| GO:0001883~purine nucleoside binding | 141 | 16.73 | 3.58E-05 |
| GO:0030554~adenyl nucleotide binding | 141 | 16.73 | 3.58E-05 |
| GO:0001882~nucleoside binding | 141 | 16.73 | 4.20E-05 |
| GO:0005524~ATP binding | 130 | 15.42 | 9.79E-05 |
| GO:0032553~ribonucleotide binding | 138 | 16.37 | 6.14E-04 |
| GO:0032555~purine ribonucleotide binding | 138 | 16.37 | 6.14E-04 |
| GO:0017076~purine nucleotide binding | 145 | 17.20 | 6.77E-04 |
| GO:0000166~nucleotide binding | 158 | 18.74 | 0.015997 |
|  |  |  |  |
| Cluster 3; Enrichment Score: 8.32 | | | |
| GO:0042742~defense response to bacterium | 36 | 4.27 | 5.83E-15 |
| GO:0009617~response to bacterium | 40 | 4.74 | 6.36E-14 |
| GO:0009814~defense response, incompatible interaction | 20 | 2.37 | 5.11E-09 |
| GO:0006952~defense response | 77 | 9.13 | 6.63E-08 |
| GO:0009627~systemic acquired resistance | 10 | 1.19 | 2.69E-06 |
| GO:0006955~immune response | 30 | 3.56 | 4.99E-06 |
| GO:0045087~innate immune response | 27 | 3.20 | 3.40E-05 |
|  |  |  |  |
| Cluster 4; Enrichment Score: 7.42 | | | |
| glycoprotein | 84 | 9.96 | 6.81E-15 |
| signal | 95 | 11.27 | 1.41E-10 |
| glycosylation site:N-linked (GlcNAc...) | 79 | 9.37 | 8.86E-09 |
| active site:Proton acceptor | 48 | 5.69 | 8.12E-07 |
| signal peptide | 95 | 11.27 | 1.06E-05 |
| Secreted | 40 | 4.74 | 0.039929 |
|  |  |  |  |
| Cluster 5; Enrichment Score: 6.58 | | | |
| PIRSF036788:receptor-like protein kinase | 15 | 1.78 | 2.81E-10 |
| IPR002902:Protein of unknown function DUF26 | 17 | 2.02 | 8.47E-07 |
| domain:Gnk2-homologous 2 | 17 | 2.02 | 4.45E-06 |
| domain:Gnk2-homologous 1 | 17 | 2.02 | 4.45E-06 |
|  |  |  |  |
| Cluster 6; Enrichment Score: 6.07 | | | |
| SM00774:WRKY | 16 | 1.90 | 6.09E-09 |
| IPR003657:DNA-binding WRKY | 16 | 1.90 | 3.62E-08 |
| DNA-binding region:WRKY | 14 | 1.66 | 5.40E-07 |
| GO:0043565~sequence-specific DNA binding | 33 | 3.91 | 0.004469 |
|  |  |  |  |
| Cluster 7; Enrichment Score: 5.81 | | | |
| GO:0005618~cell wall | 47 | 5.58 | 1.37E-06 |
| GO:0009505~plant-type cell wall | 28 | 3.32 | 1.38E-06 |
| GO:0030312~external encapsulating structure | 47 | 5.58 | 2.03E-06 |
|  |  |  |  |
| Cluster 8; Enrichment Score: 4.80 | | | |
| IPR001611:Leucine-rich repeat | 37 | 4.39 | 2.85E-06 |
| leucine-rich repeat | 39 | 4.63 | 2.94E-06 |
| IPR013210:Leucine-rich repeat, N-terminal | 19 | 2.25 | 4.75E-04 |
|  |  |  |  |
| Cluster 9; Enrichment Score: 3.79 | | | |
| IPR000152:EGF-type aspartate/asparagine hydroxylation conserved site | 7 | 0.83 | 2.82E-06 |
| SM00181:EGF | 8 | 0.95 | 1.53E-05 |
| IPR006210:EGF-like | 8 | 0.95 | 3.43E-05 |
| egf-like domain | 6 | 0.71 | 4.55E-05 |
| IPR018097:EGF-like calcium-binding, conserved site | 7 | 0.83 | 6.97E-05 |
| domain:EGF-like 1 | 6 | 0.71 | 7.01E-05 |
| SM00179:EGF_CA | 4 | 0.47 | 3.01E-04 |
| IPR001881:EGF-like calcium-binding | 4 | 0.47 | 4.30E-04 |
| IPR000742:EGF-like, type 3 | 5 | 0.59 | 4.36E-04 |
| domain:EGF-like 2; calcium-binding | 4 | 0.47 | 6.41E-04 |
| PIRSF000575:wall-associated protein kinase | 6 | 0.71 | 0.001368 |
| IPR013032:EGF-like region, conserved site | 7 | 0.83 | 0.001586 |
| IPR013091:EGF calcium-binding | 4 | 0.47 | 0.002222 |
|  |  |  |  |
| Cluster 10; Enrichment Score: 3.70 | | | |
| Lectin | 13 | 1.54 | 2.81E-06 |
| PIRSF036780:putative receptor-like protein kinase, plant type | 7 | 0.83 | 4.30E-04 |
| IPR001220:Legume lectin, beta chain | 9 | 1.07 | 5.45E-04 |
| IPR013320:Concanavalin A-like lectin/glucanase, subgroup | 11 | 1.30 | 0.002438 |

^a^ GO terms with enrichment score>0.5 and P<0.05 were considered to be significantly enriched.

^b^ Percentage of total [functional](javascript:void(0);) [annotation](javascript:void(0);) genes.

**Top ten GO terms of transgressively down- regulated genes in S.**BBCC

| **Term^a^** | **Count** | **%^b^** | **P-Value** |
| --- | --- | --- | --- |
| Cluster 1; Enrichment Score: 3.74 | | | |
| GO:0009628~response to abiotic stimulus | 43 | 11.53 | 1.10E-05 |
| GO:0006970~response to osmotic stress | 19 | 5.09 | 2.47E-04 |
| GO:0009651~response to salt stress | 16 | 4.29 | 0.002266 |
|  |  |  |  |
| Cluster 2; Enrichment Score: 3.46 | | | |
| GO:0046686~response to cadmium ion | 18 | 4.83 | 6.79E-05 |
| GO:0010038~response to metal ion | 18 | 4.83 | 4.57E-04 |
| GO:0010035~response to inorganic substance | 21 | 5.63 | 0.001379 |
|  |  |  |  |
| Cluster 3; Enrichment Score: 2.83 | | | |
| ribosomal protein | 17 | 4.56 | 4.90E-05 |
| GO:0022626~cytosolic ribosome | 17 | 4.56 | 8.55E-05 |
| GO:0003735~structural constituent of ribosome | 18 | 4.83 | 3.10E-04 |
| GO:0005840~ribosome | 20 | 5.36 | 3.44E-04 |
| ribonucleoprotein | 16 | 4.29 | 4.17E-04 |
| GO:0033279~ribosomal subunit | 15 | 4.02 | 4.29E-04 |
| GO:0022625~cytosolic large ribosomal subunit | 10 | 2.68 | 5.11E-04 |
| GO:0005198~structural molecule activity | 21 | 5.63 | 6.29E-04 |
| GO:0044445~cytosolic part | 14 | 3.75 | 6.42E-04 |
| GO:0015934~large ribosomal subunit | 11 | 2.95 | 6.56E-04 |
| GO:0005829~cytosol | 24 | 6.43 | 0.001739 |
| GO:0030529~ribonucleoprotein complex | 22 | 5.90 | 0.004233 |
| GO:0022613~ribonucleoprotein complex biogenesis | 12 | 3.22 | 0.004622 |
| GO:0043232~intracellular non-membrane-bounded organelle | 32 | 8.58 | 0.00493 |
| GO:0043228~non-membrane-bounded organelle | 32 | 8.58 | 0.00493 |
| GO:0042254~ribosome biogenesis | 11 | 2.95 | 0.010615 |
| ath03010:Ribosome | 12 | 3.22 | 0.031731 |
|  |  |  |  |
| Cluster 4; Enrichment Score: 2.46 | | | |
| GO:0045271~respiratory chain complex I | 6 | 1.61 | 8.80E-04 |
| GO:0030964~NADH dehydrogenase complex | 6 | 1.61 | 8.80E-04 |
| GO:0070469~respiratory chain | 8 | 2.14 | 0.001336 |
| GO:0031966~mitochondrial membrane | 12 | 3.22 | 0.001524 |
| GO:0043094~cellular metabolic compound salvage | 5 | 1.34 | 0.02872 |
| GO:0009853~photorespiration | 4 | 1.07 | 0.037235 |
|  |  |  |  |
| Cluster 5; Enrichment Score: 2.34 | | | |
| GO:0019438~aromatic compound biosynthetic process | 13 | 3.49 | 4.26E-04 |
| GO:0009809~lignin biosynthetic process | 6 | 1.61 | 0.001108 |
| GO:0009699~phenylpropanoid biosynthetic process | 9 | 2.41 | 0.001898 |
| GO:0009808~lignin metabolic process | 6 | 1.61 | 0.005642 |
| GO:0019748~secondary metabolic process | 16 | 4.29 | 0.007941 |
| GO:0009698~phenylpropanoid metabolic process | 9 | 2.41 | 0.00909 |
| GO:0042398~cellular amino acid derivative biosynthetic process | 9 | 2.41 | 0.02143 |
| GO:0006575~cellular amino acid derivative metabolic process | 11 | 2.95 | 0.026401 |
|  |  |  |  |
| Cluster 6; Enrichment Score: 2.06 | | | |
| GO:0016192~vesicle-mediated transport | 15 | 4.02 | 6.27E-04 |
| GO:0045184~establishment of protein localization | 20 | 5.36 | 0.001022 |
| GO:0015031~protein transport | 20 | 5.36 | 0.001022 |
| protein transport | 11 | 2.95 | 0.001471 |
| GO:0008104~protein localization | 20 | 5.36 | 0.001521 |
| golgi apparatus | 10 | 2.68 | 0.001652 |
| GO:0005794~Golgi apparatus | 14 | 3.75 | 0.008401 |
| GO:0046907~intracellular transport | 15 | 4.02 | 0.010586 |
| GO:0070727~cellular macromolecule localization | 12 | 3.22 | 0.01839 |
| GO:0006886~intracellular protein transport | 11 | 2.95 | 0.02306 |
| GO:0044431~Golgi apparatus part | 6 | 1.61 | 0.02802 |
| GO:0034613~cellular protein localization | 11 | 2.95 | 0.029468 |
| transport | 23 | 6.17 | 0.053859 |
| GO:0008565~protein transporter activity | 6 | 1.61 | 0.073672 |
|  |  |  |  |
| Cluster 7; Enrichment Score: 2.04 | | | |
| GO:0009809~lignin biosynthetic process | 6 | 1.61 | 0.001108 |
| GO:0009808~lignin metabolic process | 6 | 1.61 | 0.005642 |
| GO:0016621~cinnamoyl-CoA reductase activity | 3 | 0.80 | 0.011243 |
| IPR001509:NAD-dependent epimerase/dehydratase | 5 | 1.34 | 0.021463 |
| GO:0016620~oxidoreductase activity, acting on the aldehyde or oxo group of donors, NAD or NADP as acceptor | 4 | 1.07 | 0.042583 |
|  |  |  |  |
| Cluster 8; Enrichment Score: 1.97 | | | |
| GO:0031974~membrane-enclosed lumen | 22 | 5.90 | 0.003957 |
| GO:0070013~intracellular organelle lumen | 21 | 5.63 | 0.00725 |
| GO:0043233~organelle lumen | 21 | 5.63 | 0.00725 |
| GO:0005730~nucleolus | 12 | 3.22 | 0.023851 |
| GO:0031981~nuclear lumen | 15 | 4.02 | 0.027191 |
|  |  |  |  |
| Cluster 9; Enrichment Score: 1.94 | | | |
| GO:0044429~mitochondrial part | 19 | 5.09 | 4.81E-06 |
| GO:0005740~mitochondrial envelope | 14 | 3.75 | 2.08E-04 |
| GO:0005739~mitochondrion | 40 | 10.72 | 4.60E-04 |
| GO:0031966~mitochondrial membrane | 12 | 3.22 | 0.001524 |
| GO:0031090~organelle membrane | 27 | 7.24 | 0.002015 |
| mitochondrion | 14 | 3.75 | 0.035786 |
| GO:0005743~mitochondrial inner membrane | 8 | 2.14 | 0.0376 |
| GO:0031967~organelle envelope | 20 | 5.36 | 0.043631 |
| GO:0031975~envelope | 20 | 5.36 | 0.046632 |
|  |  |  |  |
| Cluster 10; Enrichment Score: 1.66 | | | |
| Term | Count | % | PValue |
| GO:0009629~response to gravity | 5 | 1.34 | 0.006172 |
| GO:0009630~gravitropism | 4 | 1.07 | 0.030791 |

^a^ GO terms with enrichment score>0.5 and P<0.05 were considered to be significantly enriched.

^b^ Percentage of total [functional](javascript:void(0);) [annotation](javascript:void(0);) genes.

**Top ten GO terms of transgressively up- regulated genes in S.**CCAA

| **Term^b^** | **Count** | **%^b^** | **P-Value** |
| --- | --- | --- | --- |
| Cluster 1; Enrichment Score: 3.03 | | | |
| GO:0009617~response to bacterium | 18 | 2.98 | 2.67E-05 |
| GO:0042742~defense response to bacterium | 15 | 2.48 | 5.32E-05 |
| GO:0006955~immune response | 14 | 2.31 | 0.0125401 |
| GO:0006952~defense response | 32 | 5.29 | 0.0432483 |
|  |  |  |  |
| Cluster 2; Enrichment Score: 2.72 | | | |
| GO:0009834~secondary cell wall biogenesis | 7 | 1.16 | 1.55E-06 |
| GO:0010382~cellular cell wall macromolecule metabolic process | 5 | 0.83 | 1.37E-04 |
| GO:0042546~cell wall biogenesis | 9 | 1.49 | 1.86E-04 |
| GO:0010383~cell wall polysaccharide metabolic process | 5 | 0.83 | 2.53E-04 |
| GO:0045492~xylan biosynthetic process | 4 | 0.66 | 5.34E-04 |
| GO:0070592~cell wall polysaccharide biosynthetic process | 4 | 0.66 | 5.34E-04 |
| GO:0010417~glucuronoxylan biosynthetic process | 4 | 0.66 | 5.34E-04 |
| GO:0010413~glucuronoxylan metabolic process | 4 | 0.66 | 5.34E-04 |
| GO:0009832~plant-type cell wall biogenesis | 7 | 1.16 | 0.0010055 |
| GO:0045491~xylan metabolic process | 4 | 0.66 | 0.0014983 |
| GO:0070589~cellular component macromolecule biosynthetic process | 4 | 0.66 | 0.0014983 |
| GO:0044038~cell wall macromolecule biosynthetic process | 4 | 0.66 | 0.0014983 |
| GO:0010410~hemicellulose metabolic process | 4 | 0.66 | 0.0014983 |
| GO:0044036~cell wall macromolecule metabolic process | 6 | 0.99 | 0.0045788 |
| GO:0034637~cellular carbohydrate biosynthetic process | 11 | 1.82 | 0.008753 |
| GO:0044264~cellular polysaccharide metabolic process | 9 | 1.49 | 0.0199537 |
| GO:0016051~carbohydrate biosynthetic process | 12 | 1.98 | 0.0243576 |
| GO:0033692~cellular polysaccharide biosynthetic process | 7 | 1.16 | 0.0254207 |
| GO:0000271~polysaccharide biosynthetic process | 7 | 1.16 | 0.0299227 |
|  |  |  |  |
| Cluster 3; Enrichment Score: 2.30 | | | |
| GO:0009808~lignin metabolic process | 9 | 1.49 | 8.14E-05 |
| GO:0009698~phenylpropanoid metabolic process | 13 | 2.15 | 2.91E-04 |
| GO:0019748~secondary metabolic process | 20 | 3.31 | 0.0024654 |
| GO:0006575~cellular amino acid derivative metabolic process | 15 | 2.48 | 0.0035086 |
| GO:0009699~phenylpropanoid biosynthetic process | 9 | 1.49 | 0.0070763 |
| GO:0019438~aromatic compound biosynthetic process | 12 | 1.98 | 0.0077974 |
| GO:0009809~lignin biosynthetic process | 5 | 0.83 | 0.0165115 |
|  |  |  |  |
| Cluster 4; Enrichment Score: 2.24 | | | |
| GO:0009063~cellular amino acid catabolic process | 7 | 1.16 | 3.64E-04 |
| GO:0009310~amine catabolic process | 7 | 1.16 | 5.25E-04 |
| GO:0046395~carboxylic acid catabolic process | 8 | 1.32 | 0.0022374 |
| GO:0016054~organic acid catabolic process | 8 | 1.32 | 0.0022374 |
| GO:0009074~aromatic amino acid family catabolic process | 3 | 0.50 | 0.0122248 |
|  |  |  |  |
| Cluster 5; Enrichment Score: 1.46 | | | |
| GO:0009808~lignin metabolic process | 9 | 1.49 | 8.14E-05 |
| GO:0019439~aromatic compound catabolic process | 6 | 0.99 | 3.14E-04 |
| GO:0042219~cellular amino acid derivative catabolic process | 5 | 0.83 | 0.003455 |
| GO:0016682~oxidoreductase activity, acting on diphenols and related substances as donors, oxygen as acceptor | 4 | 0.66 | 0.0230661 |
| copper | 5 | 0.83 | 0.0271221 |
| GO:0005507~copper ion binding | 9 | 1.49 | 0.036752 |
| apoplast | 7 | 1.16 | 0.0464058 |
|  |  |  |  |
| Cluster 6; Enrichment Score: 1.25 | | | |
| GO:0009628~response to abiotic stimulus | 40 | 6.61 | 0.0074655 |
|  |  |  |  |
| Cluster 7; Enrichment Score: 1.13 | | | |
| GO:0009751~response to salicylic acid stimulus | 9 | 1.49 | 0.0173154 |
| GO:0009863~salicylic acid mediated signaling pathway | 4 | 0.66 | 0.0248434 |
|  |  |  |  |
| Cluster 8; Enrichment Score: 1.12 | | | |
| IPR002912:Amino acid-binding ACT | 4 | 0.66 | 0.0191683 |
|  |  |  |  |
| Cluster 9; Enrichment Score: 0.88 | | | |
| GO:0009816~defense response to bacterium, incompatible interaction | 5 | 0.83 | 0.0022894 |
| GO:0006955~immune response | 14 | 2.31 | 0.0125401 |
| GO:0045087~innate immune response | 13 | 2.15 | 0.018141 |
| GO:0009814~defense response, incompatible interaction | 7 | 1.16 | 0.018675 |
| GO:0006915~apoptosis | 9 | 1.49 | 0.0354054 |
| domain:NB-ARC | 5 | 0.83 | 0.0420751 |
| PIRSF003136:disease resistance protein RPS2 | 3 | 0.50 | 0.048483 |
|  |  |  |  |
| Cluster 10; Enrichment Score: 0.76 | | | |
| GO:0042545~cell wall modification | 8 | 1.32 | 0.0288156 |
| GO:0005618~cell wall | 23 | 3.80 | 0.0297301 |
| GO:0030312~external encapsulating structure | 23 | 3.80 | 0.034115 |

^a^ GO terms with enrichment score>0.5 and P<0.05 were considered to be significantly enriched.

^b^ Percentage of total [functional](javascript:void(0);) [annotation](javascript:void(0);) genes.

**Top ten GO terms of transgressively down- regulated genes in S.**CCAA

| **Term^a^** | **Count** | **%^b^** | **P-Value** |
| --- | --- | --- | --- |
| Cluster 1; Enrichment Score: 9.02 | | | |
| GO:0005618~cell wall | 40 | 8.73 | 2.61E-10 |
| GO:0030312~external encapsulating structure | 40 | 8.73 | 4.00E-10 |
| GO:0009505~plant-type cell wall | 24 | 5.24 | 8.30E-09 |
|  |  |  |  |
| Cluster 2; Enrichment Score: 4.15 | | | |
| glycoprotein | 47 | 10.26 | 4.61E-09 |
| glycosylation site:N-linked (GlcNAc...) | 47 | 10.26 | 8.88E-07 |
| signal | 53 | 11.57 | 1.16E-06 |
| signal peptide | 53 | 11.57 | 5.62E-04 |
| Secreted | 28 | 6.11 | 0.005327 |
| disulfide bond | 23 | 5.02 | 0.015389 |
| GO:0005576~extracellular region | 35 | 7.64 | 0.043559 |
|  |  |  |  |
| Cluster 3; Enrichment Score: 3.93 | | | |
| IPR018525:DNA-dependent ATPase MCM, conserved site | 6 | 1.31 | 1.58E-08 |
| **DNA** replication | 9 | 1.97 | 4.65E-08 |
| SM00350:MCM | 6 | 1.31 | 8.00E-08 |
| IPR001208:DNA-dependent ATPase MCM | 6 | 1.31 | 1.43E-07 |
| GO:0006270~DNA replication initiation | 6 | 1.31 | 2.59E-06 |
| GO:0006268~DNA unwinding during replication | 6 | 1.31 | 2.59E-06 |
| ath03030:DNA replication | 9 | 1.97 | 3.58E-06 |
| GO:0008094~DNA-dependent ATPase activity | 8 | 1.75 | 1.27E-05 |
| GO:0032392~DNA geometric change | 6 | 1.31 | 1.34E-05 |
| GO:0032508~DNA duplex unwinding | 6 | 1.31 | 1.34E-05 |
| GO:0006260~DNA replication | 12 | 2.62 | 4.92E-05 |
| IPR012340:Nucleic acid-binding, OB-fold | 8 | 1.75 | 0.002219 |
| GO:0006259~DNA metabolic process | 17 | 3.71 | 0.002657 |
| GO:0006261~DNA-dependent DNA replication | 6 | 1.31 | 0.003047 |
|  |  |  |  |
| Cluster 4; Enrichment Score: 3.63 | | | |
| IPR013781:Glycoside hydrolase, subgroup, catalytic core | 14 | 3.06 | 1.41E-05 |
| glycosidase | 17 | 3.71 | 6.94E-05 |
| active site:Nucleophile | 14 | 3.06 | 9.27E-04 |
| active site:Proton donor | 13 | 2.84 | 0.003438 |
|  |  |  |  |
| Cluster 5; Enrichment Score: 3.34 | | | |
| metal ion-binding site:Calcium | 7 | 1.53 | 1.06E-05 |
| lyase | 14 | 3.06 | 1.23E-05 |
| SM00656:Amb_all | 6 | 1.31 | 7.30E-05 |
| IPR018082:AmbAllergen | 6 | 1.31 | 1.26E-04 |
| IPR002022:Pectate lyase/Amb allergen | 6 | 1.31 | 1.26E-04 |
| GO:0030570~pectate lyase activity | 6 | 1.31 | 1.87E-04 |
| GO:0016837~carbon-oxygen lyase activity, acting on polysaccharides | 6 | 1.31 | 1.87E-04 |
| calcium | 16 | 3.49 | 5.37E-04 |
| PIRSF006065:pectate lyase LAT59 | 5 | 1.09 | 8.64E-04 |
| IPR012334:Pectin lyase fold | 9 | 1.97 | 0.0117 |
| SM00710:PbH1 | 5 | 1.09 | 0.04862 |
|  |  |  |  |
| Cluster 6; Enrichment Score: 2.36 | | | |
| GO:0009628~response to abiotic stimulus | 49 | 10.70 | 6.39E-06 |
| GO:0009416~response to light stimulus | 18 | 3.93 | 0.017449 |
| GO:0009314~response to radiation | 18 | 3.93 | 0.023161 |
|  |  |  |  |
| Cluster 7; Enrichment Score: 2.28 | | | |
| GO:0006633~fatty acid biosynthetic process | 11 | 2.40 | 0.001766 |
| GO:0046394~carboxylic acid biosynthetic process | 19 | 4.15 | 0.002162 |
| GO:0016053~organic acid biosynthetic process | 19 | 4.15 | 0.002162 |
| GO:0006631~fatty acid metabolic process | 12 | 2.62 | 0.005101 |
| GO:0008610~lipid biosynthetic process | 18 | 3.93 | 0.005971 |
| lipid synthesis | 7 | 1.53 | 0.006148 |
|  |  |  |  |
| Cluster 8; Enrichment Score: 2.14 | | | |
| cell wall biogenesis/degradation | 14 | 3.06 | 7.54E-04 |
| GO:0007047~cell wall organization | 15 | 3.28 | 0.001952 |
| GO:0045229~external encapsulating structure organization | 15 | 3.28 | 0.003225 |
| cell wall | 8 | 1.75 | 0.018873 |
|  |  |  |  |
| Cluster 9; Enrichment Score: 1.50 | | | |
| membrane | 61 | 13.32 | 7.82E-04 |
| transmembrane | 51 | 11.14 | 0.002849 |
|  |  |  |  |
| Cluster 10; Enrichment Score: 1.42 | | | |
| SM00045:DAGKa | 3 | 0.66 | 0.004317 |
| IPR000756:Diacylglycerol kinase accessory region | 3 | 0.66 | 0.005344 |
| SM00046:DAGKc | 3 | 0.66 | 0.012373 |
| IPR001206:Diacylglycerol kinase, catalytic region | 3 | 0.66 | 0.015229 |
| GO:0007205~activation of protein kinase C activity by G-protein coupled receptor protein signaling pathway | 3 | 0.66 | 0.020794 |
| GO:0032147~activation of protein kinase activity | 3 | 0.66 | 0.020794 |
| GO:0004143~diacylglycerol kinase activity | 3 | 0.66 | 0.021601 |
| GO:0042325~regulation of phosphorylation | 4 | 0.87 | 0.030375 |
| GO:0045860~positive regulation of protein kinase activity | 3 | 0.66 | 0.033031 |
| GO:0033674~positive regulation of kinase activity | 3 | 0.66 | 0.033031 |
| GO:0019220~regulation of phosphate metabolic process | 4 | 0.87 | 0.038002 |
| GO:0051174~regulation of phosphorus metabolic process | 4 | 0.87 | 0.038002 |
| GO:0051347~positive regulation of transferase activity | 3 | 0.66 | 0.047406 |

^a^ GO terms with enrichment score>0.5 and P<0.05 were considered to be significantly enriched.

^b^ Percentage of total [functional](javascript:void(0);) [annotation](javascript:void(0);) genes.

**Top ten GO terms of transgressively up- regulated genes in S.AA**CC

| **Term^a^** | **Count** | **%^b^** | **P-Value** |
| --- | --- | --- | --- |
| Cluster 1; Enrichment Score: 9.11 | | | |
| GO:0009617~response to bacterium | 46 | 3.73 | 9.14E-13 |
| GO:0042742~defense response to bacterium | 36 | 2.92 | 1.33E-10 |
| GO:0006952~defense response | 93 | 7.55 | 3.77E-06 |
|  |  |  |  |
| Cluster 2;Enrichment Score: 8.83 | | | |
| GO:0006790~sulfur metabolic process | 44 | 3.57 | 2.15E-17 |
| GO:0044272~sulfur compound biosynthetic process | 33 | 2.68 | 1.53E-15 |
| GO:0019760~glucosinolate metabolic process | 17 | 1.38 | 7.38E-10 |
| GO:0019757~glycosinolate metabolic process | 17 | 1.38 | 7.38E-10 |
| GO:0016143~S-glycoside metabolic process | 17 | 1.38 | 7.38E-10 |
| GO:0019758~glycosinolate biosynthetic process | 14 | 1.14 | 1.38E-09 |
| GO:0016144~S-glycoside biosynthetic process | 14 | 1.14 | 1.38E-09 |
| GO:0019761~glucosinolate biosynthetic process | 14 | 1.14 | 1.38E-09 |
| GO:0016137~glycoside metabolic process | 21 | 1.70 | 2.48E-08 |
| GO:0016138~glycoside biosynthetic process | 18 | 1.46 | 2.91E-08 |
| ath00966:Glucosinolate biosynthesis | 11 | 0.89 | 4.50E-08 |
| GO:0034637~cellular carbohydrate biosynthetic process | 25 | 2.03 | 1.40E-04 |
| GO:0016051~carbohydrate biosynthetic process | 28 | 2.27 | 9.92E-04 |
|  |  |  |  |
| Cluster 3; Enrichment Score: 8.70 | | | |
| oxidoreductase | 116 | 9.42 | 7.02E-23 |
| iron | 75 | 6.09 | 7.99E-14 |
| heme | 51 | 4.14 | 2.48E-13 |
| GO:0055114~oxidation reduction | 126 | 10.23 | 1.28E-12 |
| Monooxygenase | 39 | 3.17 | 8.90E-11 |
| GO:0020037~heme binding | 55 | 4.46 | 1.14E-10 |
| metal ion-binding site:Iron (heme axial ligand) | 34 | 2.76 | 3.29E-10 |
| GO:0005506~iron ion binding | 84 | 6.82 | 3.43E-10 |
| GO:0046906~tetrapyrrole binding | 55 | 4.46 | 2.05E-09 |
| IPR017972:Cytochrome P450, conserved site | 35 | 2.84 | 1.61E-08 |
| IPR001128:Cytochrome P450 | 36 | 2.92 | 4.80E-08 |
| IPR002401:Cytochrome P450, E-class, group I | 34 | 2.76 | 6.78E-08 |
| IPR017973:Cytochrome P450, C-terminal region | 33 | 2.68 | 1.30E-07 |
| GO:0009055~electron carrier activity | 71 | 5.76 | 1.49E-07 |
| GO:0019825~oxygen binding | 35 | 2.84 | 2.64E-07 |
| PIRSF000045:cytochrome P450 CYP2D6 | 23 | 1.87 | 2.12E-06 |
| Secondary metabolites biosynthesis, transport, and catabolism | 38 | 3.08 | 9.06E-05 |
| ath00945:Stilbenoid, diarylheptanoid and gingerol biosynthesis | 13 | 1.06 | 6.86E-04 |
| ath00903:Limonene and pinene degradation | 12 | 0.97 | 0.003464 |
|  |  |  |  |
| Cluster 4; Enrichment Score: 6.29 | | | |
| iron | 75 | 6.09 | 7.99E-14 |
| GO:0005506~iron ion binding | 84 | 6.82 | 3.43E-10 |
| metal-binding | 142 | 11.53 | 8.99E-08 |
| GO:0043167~ion binding | 246 | 19.97 | 1.70E-04 |
| GO:0046872~metal ion binding | 234 | 18.99 | 2.32E-04 |
| GO:0043169~cation binding | 244 | 19.81 | 2.70E-04 |
| GO:0046914~transition metal ion binding | 194 | 15.75 | 3.73E-04 |
|  |  |  |  |
| Cluster 5; Enrichment Score: 6.13 | | | |
| amino-acid biosynthesis | 22 | 1.79 | 1.04E-08 |
| GO:0009309~amine biosynthetic process | 33 | 2.68 | 1.05E-07 |
| GO:0008652~cellular amino acid biosynthetic process | 31 | 2.52 | 1.09E-07 |
| GO:0016053~organic acid biosynthetic process | 49 | 3.98 | 7.76E-07 |
| GO:0046394~carboxylic acid biosynthetic process | 49 | 3.98 | 7.76E-07 |
| GO:0044271~nitrogen compound biosynthetic process | 45 | 3.65 | 0.002318 |
|  |  |  |  |
| Cluster 6; Enrichment Score: 6.10 | | | |
| SM00774:WRKY | 19 | 1.54 | 3.94E-10 |
| IPR003657:DNA-binding WRKY | 19 | 1.54 | 2.37E-08 |
| DNA-binding region:WRKY | 17 | 1.38 | 8.22E-08 |
| GO:0043565~sequence-specific DNA binding | 28 | 2.27 | 0.540333 |
|  |  |  |  |
| Cluster 7; Enrichment Score: 6.03 | | | |
| GO:0010033~response to organic substance | 117 | 9.50 | 6.09E-10 |
| GO:0009719~response to endogenous stimulus | 91 | 7.39 | 1.29E-06 |
| GO:0009725~response to hormone stimulus | 74 | 6.01 | 0.001012 |
|  |  |  |  |
| Cluster 8; Enrichment Score: 5.91 | | | |
| transmembrane | 155 | 12.58 | 1.75E-11 |
| membrane | 166 | 13.47 | 6.09E-09 |
| GO:0016021~integral to membrane | 160 | 12.99 | 1.83E-06 |
| transmembrane region | 135 | 10.96 | 4.90E-06 |
| GO:0031224~intrinsic to membrane | 178 | 14.45 | 7.47E-05 |
| transport | 77 | 6.25 | 1.22E-04 |
| topological domain:Cytoplasmic | 56 | 4.55 | 4.54E-04 |
|  |  |  |  |
| Cluster 9; Enrichment Score: 5.40 | | | |
| GO:0006970~response to osmotic stress | 48 | 3.90 | 9.76E-07 |
| GO:0009651~response to salt stress | 45 | 3.65 | 1.56E-06 |
| GO:0009628~response to abiotic stimulus | 100 | 8.12 | 4.09E-05 |
|  |  |  |  |
| Cluster 10; Enrichment Score: 4.35 | | | |
| GO:0010035~response to inorganic substance | 59 | 4.79 | 1.19E-06 |
| GO:0010038~response to metal ion | 40 | 3.25 | 2.18E-04 |
| GO:0046686~response to cadmium ion | 35 | 2.84 | 3.54E-04 |

^a^ GO terms with enrichment score>0.5 and P<0.05 were considered to be significantly enriched.

^b^ Percentage of total [functional](javascript:void(0);) [annotation](javascript:void(0);) genes.

**Top ten GO terms of transgressively down- regulated genes in S.AA**CC

| **Term^a^** | **Count** | **%^b^** | **P-Value** |
| --- | --- | --- | --- |
| Cluster 1; Enrichment Score: 3.28 | | | |
| GO:0009628~response to abiotic stimulus | 66 | 7.81 | 3.59E-05 |
| GO:0009651~response to salt stress | 25 | 2.96 | 0.001183 |
| GO:0006970~response to osmotic stress | 25 | 2.96 | 0.003313 |
|  |  |  |  |
| Cluster 2; Enrichment Score: 3.11 | | | |
| GO:0010025~wax biosynthetic process | 6 | 0.71 | 1.21E-04 |
| GO:0010166~wax metabolic process | 6 | 0.71 | 1.67E-04 |
| GO:0006633~fatty acid biosynthetic process | 16 | 1.89 | 2.39E-04 |
| GO:0016053~organic acid biosynthetic process | 27 | 3.20 | 0.001007 |
| GO:0046394~carboxylic acid biosynthetic process | 27 | 3.20 | 0.001007 |
| GO:0006631~fatty acid metabolic process | 17 | 2.01 | 0.001917 |
| GO:0008610~lipid biosynthetic process | 23 | 2.72 | 0.019189 |
|  |  |  |  |
| Cluster 3; Enrichment Score: 2.05 | | | |
| GO:0042335~cuticle development | 5 | 0.59 | 0.002441 |
| GO:0000038~very-long-chain fatty acid metabolic process | 5 | 0.59 | 0.004416 |
| domain:FAE | 5 | 0.59 | 0.004503 |
| IPR012392:Very-long-chain 3-ketoacyl-CoA synthase | 5 | 0.59 | 0.005199 |
| IPR013601:FAE1/Type III polyketide synthase-like protein | 5 | 0.59 | 0.005199 |
| PIRSF036417:3-ktacl-CoA_syn | 5 | 0.59 | 0.006864 |
| Acyltransferase | 11 | 1.30 | 0.012749 |
| IPR016038:Thiolase-like, subgroup | 5 | 0.59 | 0.025716 |
|  |  |  |  |
| Cluster 5; Enrichment Score: 2.00 | | | |
| GO:0005618~cell wall | 35 | 4.14 | 0.002137 |
| GO:0030312~external encapsulating structure | 35 | 4.14 | 0.002713 |
|  |  |  |  |
| Cluster 5; Enrichment Score: 1.69 | | | |
| GO:0000902~cell morphogenesis | 15 | 1.78 | 0.008923 |
| GO:0016049~cell growth | 15 | 1.78 | 0.011293 |
| GO:0040007~growth | 16 | 1.89 | 0.016157 |
| GO:0008361~regulation of cell size | 15 | 1.78 | 0.017444 |
| GO:0032989~cellular component morphogenesis | 15 | 1.78 | 0.020664 |
| GO:0032535~regulation of cellular component size | 15 | 1.78 | 0.025905 |
| GO:0060560~developmental growth involved in morphogenesis | 11 | 1.30 | 0.033258 |
| GO:0009826~unidimensional cell growth | 11 | 1.30 | 0.033258 |
| GO:0048589~developmental growth | 12 | 1.42 | 0.035593 |
|  |  |  |  |
| Cluster 6; Enrichment Score: 1.69 | | | |
| gpi-anchor | 10 | 1.18 | 0.005606 |
| propeptide:Removed in mature form | 12 | 1.42 | 0.005662 |
| GO:0046658~anchored to plasma membrane | 8 | 0.95 | 0.006005 |
| lipid moiety-binding region:GPI-anchor amidated serine | 8 | 0.95 | 0.008802 |
| cell membrane | 23 | 2.72 | 0.012619 |
| lipoprotein | 14 | 1.66 | 0.016133 |
|  |  |  |  |
| Cluster 7; Enrichment Score: 1.56 | | | |
| GO:0019319~hexose biosynthetic process | 4 | 0.47 | 0.019925 |
| GO:0046364~monosaccharide biosynthetic process | 4 | 0.47 | 0.023096 |
| GO:0046165~alcohol biosynthetic process | 5 | 0.59 | 0.03232 |
| GO:0019318~hexose metabolic process | 11 | 1.30 | 0.037162 |
|  |  |  |  |
| Cluster 8; Enrichment Score: 1.52 | | | |
| domain:Leucine-zipper | 8 | 0.95 | 0.002335 |
| SM00340:HALZ | 5 | 0.59 | 0.004399 |
| IPR003106:Leucine zipper, homeobox-associated | 5 | 0.59 | 0.005199 |
| DNA binding | 11 | 1.30 | 0.005918 |
| GO:0016563~transcription activator activity | 10 | 1.18 | 0.046867 |
|  |  |  |  |
| Cluster 9; Enrichment Score: 1.40 | | | |
| IPR003311:AUX/IAA protein | 7 | 0.83 | 8.10E-04 |
| short sequence motif:EAR-like (transcriptional repression) | 7 | 0.83 | 0.00162 |
| IPR011525:Aux/IAA-ARF-dimerisation | 7 | 0.83 | 0.005229 |
| domain:Aux/IAA-ARF | 7 | 0.83 | 0.00527 |
| repressor | 10 | 1.18 | 0.008032 |
| GO:0046983~protein dimerization activity | 16 | 1.89 | 0.018783 |
| GO:0009725~response to hormone stimulus | 41 | 4.85 | 0.036099 |
| GO:0009719~response to endogenous stimulus | 43 | 5.09 | 0.043102 |
| GO:0007242~intracellular signaling cascade | 36 | 4.26 | 0.044345 |
|  |  |  |  |
| Cluster 10; Enrichment Score: 1.39 | | | |
| GO:0009642~response to light intensity | 8 | 0.95 | 0.009959 |

^a^ GO terms with enrichment score>0.5 and P<0.05 were considered to be significantly enriched.

^b^ Percentage of total [functional](javascript:void(0);) [annotation](javascript:void(0);) genes.
