# Supplementary material for: Genome-specific differential gene expressions in resynthesized Brassica allotetraploids from pair-wise crosses of three cultivated diploids revealed by RNA-seq
Source: Front Plant Sci. 2015 Nov 4;6:957. doi: 10.3389/fpls.2015.00957 (PMC4631939; doi:10.3389/fpls.2015.00957)
Supplement: Supplementary Table 3 — Annotation of co-transgressively regulated genes among allotetraploids. [file Table3.DOC]

**Supplementary Table 3. Annotation of co-transgressively regulated genes among allotetraploids.**

**Co-transgressively up-regulat**ed genes in young leaves

| **Gene ID** | **GO-item** | **Annotation** |
| --- | --- | --- |
| **Bra000128** |  |  |
| **Bra000809** | GO:0006364 | rRNA processing |
|  | GO:0032040 | small-subunit processome |
| **Bra002046** | GO:0005634 | nucleus |
| **Bra002610** | GO:0032776 | DNA methylation on cytosine |
|  | GO:0015667 | site-specific DNA-methyltransferase (cytosine-N4-specific) activity |
|  | GO:0003677 | DNA binding |
| **Bra004083** | GO:0008270 | zinc ion binding |
|  | GO:0005198 | structural molecule activity |
|  | GO:0003676 | nucleic acid binding |
| **Bra004584** | GO:0008026 | ATP-dependent helicase activity |
|  | GO:0005524 | ATP binding |
|  | GO:0004386 | helicase activity |
|  | GO:0003676 | nucleic acid binding |
| **Bra007831** | GO:0055114 | oxidation reduction |
|  | GO:0020037 | heme binding |
|  | GO:0006979 | response to oxidative stress |
|  | GO:0004601 | peroxidase activity |
| **Bra009117** |  |  |
| **Bra011633** |  |  |
| **Bra018521** | GO:0016491 | oxidoreductase activity |
| **Bra020022** |  |  |
| **Bra022537** | GO:0008168 | methyltransferase activity |
| **Bra026724** | GO:0008026 | ATP-dependent helicase activity |
|  | GO:0005524 | ATP binding |
|  | GO:0004386 | helicase activity |
|  | GO:0003676 | nucleic acid binding |
| **Bra028228** |  |  |
| **Bra034294** |  |  |
| **Bra034380** | GO:0016538 | cyclin-dependent protein kinase regulator activity |
|  | GO:0007049 | cell cycle |
| **Bra034712** |  |  |

**Co-transgressively down-regulated genes in young leaves.**

| **Gene ID** | **GO-item** | **Annotation** |
| --- | --- | --- |
| **Bra000376** |  |  |
| **Bra000867** |  |  |
| **Bra003674** |  |  |
| **Bra003789** |  |  |
| **Bra008162** |  |  |
| **Bra009609** |  |  |
| **Bra010117** | GO:0016020 | membrane |
|  | GO:0006857 | oligopeptide transport |
|  | GO:0005215 | transporter activity |
| **Bra010482** |  |  |
| **Bra012218** |  |  |
| **Bra012747** |  |  |
| **Bra018594** | GO:0055085 | transmembrane transport |
|  | GO:0016021 | oligopeptide transport |
|  | GO:0016020 | membrane |
|  | GO:0006810 | transport |
|  | GO:0005215 | transporter activity |
| **Bra019315** | GO:0006468 | protein amino acid phosphorylation |
|  | GO:0005524 | ATP binding |
|  | GO:0004674 | protein serine/threonine kinase activity |
|  | GO:0004672 | protein kinase activity |
| **Bra024530** |  |  |
| **Bra027049** | GO:0016491 | oxidoreductase activity |
| **Bra028087** | GO:0016984 | ribulose-bisphosphate carboxylase activity |
|  | GO:0015977 | carbon utilization by fixation of carbon dioxide |
| **Bra031952** | GO:0006508 | proteolysis |
|  | GO:0004222 | metalloendopeptidase activity |
| **Bra032945** |  |  |
| **Bra038242** | GO:0055085 | transmembrane transport |
|  | GO:0016021 | integral to membrane |
|  | GO:0016020 | membrane |
|  | GO:0006810 | transport |
|  | GO:0005215 | transporter activity |

**Co-transgressively up-regulat**ed genes in silique walls.

| **Gene ID** | **GO-item** | **Annotation** |
| --- | --- | --- |
| **Bra000318** |  |  |
| **Bra001819** | GO:0008152 | metabolic process |
|  | GO:0003824 | catalytic activity |
| **Bra004333** |  |  |
| **Bra005762** | GO:0046872 | metal ion binding |
|  | GO:0030001 | metal ion transport |
| **Bra012548** | GO:0050660 | FAD binding |
|  | GO:0016614 | oxidoreductase activity, acting on CH-OH group of donors |
| **Bra016721** | GO:0016787 | hydrolase activity |
| **Bra020192** |  |  |
| **Bra020731** | GO:0055114 | oxidation reduction |
|  | GO:0006562 | proline catabolic process |
|  | GO:0004657 | proline dehydrogenase activity |
|  | GO:0006537 | glutamate biosynthetic process |
| **Bra022814** |  |  |
| **Bra023670** | GO:0045087 | innate immune response |
|  | GO:0031224 | intrinsic to membrane |
|  | GO:0017111 | nucleoside-triphosphatase activity |
|  | GO:0007156 | signal transduction |
|  | GO:0006915 | apoptosis |
|  | GO:0005524 | ATP binding |
|  | GO:0004888 | transmembrane receptor activity |
|  | GO:0000166 | nucleotide binding |
| **Bra024637** |  |  |
| **Bra024646** |  |  |
| **Bra025587** |  |  |
| **Bra027243** |  |  |
| **Bra027557** | GO:0050662 | coenzyme binding |
|  | GO:0044237 | cellular metabolic process |
|  | GO:0003824 | catalytic activity |
| **Bra028459** |  |  |
| **Bra028568** | GO:0006508 | proteolysis |
|  | GO:0004190 | aspartic-type endopeptidase activity |
| **Bra031029** | GO:0006629 | lipid metabolic process |
| **Bra034180** | GO:0016491 | oxidoreductase activity |
| **Bra035112** | GO:0008270 | zinc ion binding |
|  | GO:0005515 | protein binding |
| **Bra036724** | GO:0030170 | pyridoxal phosphate binding |
|  | GO:0008152 | metabolic process |
|  | GO:0006520 | cellular amino acid metabolic process |
|  | GO:0003824 | catalytic activity |
| **Bra039777** | GO:0016211 | ammonia ligase activity |
|  | GO:0009058 | biosynthetic process |
| **Bra040600** |  |  |

**Co-transgressively down-regulated genes in silique walls.**

| **Gene ID** | **GO-item** | **Annotation** |
| --- | --- | --- |
| **Bra006908** | GO:0016791 | phosphatase activity |
|  | GO:0016311 | dephosphorylation |
|  | GO:0008138 | protein tyrosine/serine/threonine phosphatase activity |
|  | GO:0006470 | protein dephosphorylation |
| **Bra013976** | GO:0046872 | metal ion binding |
|  | GO:0030001 | metal ion transport |
| **Bra022198** |  |  |
| **Bra031219** |  |  |
| **Bra038249** | GO:0055085 | transmembrane transport |
|  | GO:0008308 | voltage-gated anion channel activity |
|  | GO:0006820 | anion transport |
|  | GO:0005741 | mitochondrial outer membrane |
