# Supplementary material for: Genome-specific differential gene expressions in resynthesized Brassica allotetraploids from pair-wise crosses of three cultivated diploids revealed by RNA-seq
Source: Front Plant Sci. 2015 Nov 4;6:957. doi: 10.3389/fpls.2015.00957 (PMC4631939; doi:10.3389/fpls.2015.00957)
Supplement: Supplementary Table 4 — R-protein genes matched each Arabidopsis r-proteins. [file Table4.DOC]

**Supplementary Table 4. R-protein genes matched each *Arabidopsis*** r-proteins.

| **Ribosomal protein** | **Tair ID** | **Gene paralogs encoding the r-protein** | **Chromosome** |
| --- | --- | --- | --- |
| Sa | AT1G72370 | Bra008030 | A02 |
|  | AT1G72370 | Bra032101 | A04 |
|  | AT1G72370 | Bra016062 | A07 |
| **S2** | AT1G58380 | Bra027857 | A09 |
| **S3** | AT2G31610 | Bra022851 | A03 |
|  | AT2G31610 | Bra022859 | A03 |
|  | AT2G31610 | Bra018233 | A05 |
|  | AT3G53870 | Bra027914 | A09 |
|  | AT5G35530 | Bra021722 | A04 |
|  | AT5G35530 | Bra021724 | A04 |
| **S3a** | AT4G34670 | Bra011551 | A01 |
|  | AT4G34670 | Bra026324 | A01 |
|  | AT4G34670 | Bra040119 | A01 |
|  | AT4G34670 | Bra034643 | A08 |
|  | AT4G34670 | Bra001127 | A03 |
|  | AT4G34670 | Bra017669 | A03 |
|  | AT4G34670 | Bra040409 | Scaffold000203 |
| **S4** | AT5G07090 | Bra020369 | A02 |
|  | AT5G07090 | Bra028708 | A02 |
|  | AT5G07090 | Bra005118 | A05 |
|  | AT5G07090 | Bra005923 | A03 |
| **S5** | AT2G37270 | Bra038689 | A01 |
|  | AT2G37270 | Bra001428 | A03 |
|  | AT2G37270 | Bra023113 | A03 |
|  | AT2G37270 | Bra017199 | A04 |
|  | AT2G37270 | Bra005209 | A05 |
|  | AT2G37270 | Bra034799 | A05 |
| **S6** | AT5G10360 | Bra011291 | A01 |
|  | AT5G10360 | Bra028590 | A02 |
|  | AT5G10360 | Bra006056 | A03 |
|  | AT5G10360 | Bra010227 | A08 |
|  | AT5G10360 | Bra009039 | A10 |
| **S7** | AT3G02560 | Bra021431 | A01 |
|  | AT3G02560 | Bra001052 | A03 |
|  | AT3G02560 | Bra039182 | A05 |
|  | AT5G16130 | Bra006340 | A03 |
|  | AT5G16130 | Bra008643 | A10 |
| **S8** | AT5G59240 | Bra002290 | A10 |
|  | AT5G59240 | Bra006553 | A03 |
|  | AT5G59240 | Bra002290 | A10 |
| **S9** | AT5G15200 | Bra023511 | A02 |
|  | AT5G15200 | Bra006289 | A03 |
|  | AT5G15200 | Bra008713 | A10 |
| **S10** | AT4G25740 | Bra013915 | A01 |
|  | AT4G25740 | Bra019143 | A03 |
|  | AT4G25740 | Bra010448 | A08 |
|  | AT4G25740 | Bra003118 | A10 |
|  | AT5G41520 | Bra025492 | A04 |
|  | AT5G41520 | Bra028498 | A07 |
|  | AT5G52650 | Bra029112 | A03 |
| **S11** | AT5G23740 | Bra029928 | A01 |
|  | AT5G23740 | Bra029381 | A02 |
|  | AT5G23740 | Bra009706 | A06 |
|  | AT5G23740 | Bra026484 | A09 |
| **S12** | AT2G32060 | Bra022878 | A03 |
|  | AT2G32060 | Bra021773 | A04 |
|  | AT2G32060 | Bra005635 | A05 |
|  | AT2G32060 | Bra026092 | A06 |
|  | AT2G32060 | Bra016647 | A08 |
|  | AT2G32060 | Bra026739 | A09 |
| **S13** | AT4G00100 | Bra014472 | A04 |
|  | AT4G00100 | Bra040316 | A04 |
|  | AT4G00100 | Bra004901 | A05 |
|  | AT4G00100 | Bra003427 | A07 |
|  | AT4G00100 | Bra007556 | A09 |
| **S14** | AT3G11510 | Bra001406 | A03 |
|  | AT3G11510 | Bra023052 | A03 |
|  | AT3G11510 | Bra017258 | A04 |
|  | AT3G11510 | Bra005296 | A05 |
|  | AT3G11510 | Bra034833 | A05 |
|  | AT3G11510 | Bra002045 | A07 |
|  | AT3G11510 | Bra009165 | A10 |
| **S15** | AT1G04270 | Bra032519 | A09 |
|  | AT1G04270 | Bra015299 | A10 |
|  | AT5G63070 | Bra035862 | A09 |
| **S15a** | AT1G07770 | Bra018689 | A06 |
|  | AT1G07770 | Bra030697 | A08 |
|  | AT2G19720 | Bra039013 | A07 |
|  | AT4G29430 | Bra011108 | A01 |
|  | AT5G59850 | Bra020280 | A02 |
|  | AT5G59850 | Bra006689 | A03 |
|  | AT5G59850 | Bra031590 | A09 |
|  | AT5G59850 | Bra002524 | A10 |
| **S16** | AT5G18380 | Bra040145 | A01 |
|  | AT5G18380 | Bra001098 | A03 |
|  | AT5G18380 | Bra006454 | A03 |
|  | AT5G18380 | Bra025108 | A06 |
|  | AT5G18380 | Bra040207 | Scaffold000191 |
| **S17** | AT2G05220 | Bra005820 | A03 |
|  | AT2G05220 | Bra002983 | A10 |
|  | AT5G04800 | Bra028778 | A02 |
|  | AT5G04800 | Bra005887 | A03 |
|  | AT5G04800 | Bra009431 | A10 |
| **S18** | AT1G34030 | Bra028028 | A09 |
|  | AT4G09800 | Bra000692 | A03 |
|  | AT4G09800 | Bra034447 | A05 |
|  | AT4G09800 | Bra037884 | A09 |
| **S19** | AT3G02080 | Bra021478 | A01 |
|  | AT3G02080 | Bra001028 | A03 |
|  | AT3G02080 | Bra039150 | A05 |
|  | AT5G61170 | Bra029331 | A02 |
|  | AT5G61170 | Bra012980 | A03 |
|  | AT5G61170 | Bra035939 | A09 |
| **S20** | AT3G45030 | Bra038226 | A01 |
|  | AT3G45030 | Bra029167 | A03 |
|  | AT3G47370 | Bra035895 | A09 |
|  | AT3G47370 | Bra010078 | A06 |
|  | AT5G62300 | Bra022554 | A02 |
|  | AT5G62300 | Bra028263 | A10 |
| **S21** | AT5G27700 | Bra020579 | A02 |
|  | AT5G27700 | Bra014858 | A04 |
|  | AT5G27700 | Bra009947 | A06 |
|  | AT5G27700 | Bra003158 | A07 |
|  | AT5G27700 | Bra007033 | A09 |
|  | AT5G27700 | Bra036618 | A09 |
| **S23** | AT5G02960 | Bra034077 | A01 |
|  | AT5G02960 | Bra022086 | A02 |
|  | AT5G02960 | Bra028856 | A02 |
|  | AT5G02960 | Bra001300 | A03 |
|  | AT5G02960 | Bra005745 | A03 |
|  | AT5G02960 | Bra003985 | A07 |
|  | AT5G02960 | Bra009557 | A10 |
| **S24** | AT3G04920 | Bra040115 | A01 |
|  | AT3G04920 | Bra009989 | A06 |
|  | AT3G04920 | Bra036117 | A09 |
|  | AT5G28060 | Bra040415 | Scaffold000203 |
| **S25** | AT4G39200 | Bra011871 | A01 |
|  | AT4G39200 | Bra030292 | A04 |
|  | AT4G39200 | Bra033603 | A06 |
|  | AT4G39200 | Bra010696 | A08 |
|  | AT4G39200 | Bra031202 | A09 |
| **S26** | AT2G40510 | Bra000193 | A03 |
|  | AT2G40510 | Bra014697 | A04 |
|  | AT2G40510 | Bra003237 | A07 |
|  | AT2G40510 | Bra007238 | A09 |
| **S27** | AT3G61110 | Bra020722 | A02 |
|  | AT3G61110 | Bra000398 | A03 |
|  | AT3G61110 | Bra003437 | A07 |
|  | AT3G61110 | Bra007572 | A09 |
|  | AT3G61110 | Bra036186 | A09 |
| **S27a** | AT1G23410 | Bra024598 | A09 |
| **S28** | AT3G10090 | Bra037765 | A09 |
|  | AT5G03850 | Bra028814 | A02 |
|  | AT5G03850 | Bra005784 | A03 |
|  | AT5G03850 | Bra009507 | A10 |
| **S29** | AT4G33865 | Bra011477 | A01 |
|  | AT4G33865 | Bra034590 | A08 |
|  | AT4G33865 | Bra034985 | Scaffold000100 |
| **S30** | AT4G29390 | Bra039017 | A07 |
|  | AT4G29390 | Bra031096 | A09 |
|  | AT5G56670 | Bra035628 | A02 |
| **P0** | AT2G40010 | Bra000159 | A03 |
|  | AT3G11250 | Bra034055 | A01 |
| **P1** | AT1G01100 | Bra030495 | A08 |
|  | AT4G00810 | Bra000929 | A03 |
|  | AT4G00810 | Bra032620 | A09 |
|  | AT4G00810 | Bra037409 | A09 |
|  | AT5G47700 | Bra033285 | A10 |
| **P2** | AT2G27720 | Bra000501 | A03 |
|  | AT3G28500 | Bra033049 | A02 |
| **P3** | AT5G57290 | Bra020454 | A02 |
|  | AT5G57290 | Bra002741 | A10 |
| **L3** | AT1G43170 | Bra024985 | A06 |
|  | AT1G43170 | Bra027986 | A09 |
|  | AT1G43170 | Bra035298 | Scaffold000103 |
|  | AT1G43170 | Bra024986 | A06 |
|  | AT1G61580 | Bra031423 | A01 |
|  | AT1G61580 | Bra038589 | Scaffold000149 |
| **L4** | AT3G09630 | Bra034075 | A01 |
|  | AT3G09630 | Bra001302 | A03 |
|  | AT3G09630 | Bra005749 | A03 |
|  | AT3G09630 | Bra006658 | A03 |
|  | AT3G09630 | Bra029780 | A05 |
|  | AT3G09630 | Bra025887 | A06 |
|  | AT3G09630 | Bra009560 | A10 |
| **L5** | AT3G25520 | Bra013215 | A03 |
|  | AT5G39740 | Bra037493 | A06 |
| **L6** | AT1G18540 | Bra016555 | A08 |
|  | AT1G74050 | Bra008128 | A02 |
|  | AT1G74050 | Bra025895 | A06 |
|  | AT1G74050 | Bra031034 | A09 |
|  | AT1G74060 | Bra015940 | A07 |
| **L7** | AT1G80750 | Bra008443 | A02 |
|  | AT1G80750 | Bra003583 | A07 |
|  | AT2G01250 | Bra024895 | A06 |
|  | AT2G01250 | Bra014334 | A08 |
|  | AT2G01250 | Bra017461 | A09 |
|  | AT2G44120 | Bra000341 | A03 |
|  | AT2G44120 | Bra037665 | A04 |
|  | AT2G44120 | Bra004806 | A05 |
| **L7a** | AT2G47610 | Bra003512 | A07 |
|  | AT3G62870 | Bra013849 | A01 |
|  | AT3G62870 | Bra000455 | A03 |
|  | AT3G62870 | Bra019201 | A03 |
|  | AT3G62870 | Bra014387 | A04 |
|  | AT3G62870 | Bra004442 | A05 |
| **L8** | AT4G36130 | Bra011664 | A01 |
|  | AT4G36130 | Bra017749 | A03 |
|  | AT4G36130 | Bra024481 | A06 |
|  | AT4G36130 | Bra039911 | A07 |
|  | AT4G36130 | Bra010550 | A08 |
|  | AT4G36130 | Bra037242 | A09 |
| **L9** | AT1G33140 | Bra035980 | Scaffold000111 |
|  | AT1G33140 | Bra039999 | Scaffold000185 |
|  | AT4G10450 | Bra033171 | A02 |
|  | AT4G10450 | Bra037917 | A09 |
| **L10** | AT1G14320 | Bra019662 | A06 |
|  | AT1G14320 | Bra016706 | A08 |
|  | AT1G14320 | Bra026839 | A09 |
|  | AT1G66580 | Bra007877 | A02 |
|  | AT1G66580 | Bra003984 | A07 |
|  | AT1G66580 | Bra016269 | A07 |
| **L10a** | AT2G27530 | Bra012012 | A07 |
|  | AT5G22440 | Bra020193 | A02 |
|  | AT5G22440 | Bra002411 | A10 |
| **L11** | AT2G42740 | Bra021990 | A02 |
|  | AT2G42740 | Bra014558 | A04 |
|  | AT2G42740 | Bra017591 | A09 |
|  | AT3G58700 | Bra025058 | A06 |
|  | AT4G18730 | Bra013342 | A01 |
|  | AT4G18730 | Bra012580 | A03 |
| **L12** | AT3G53430 | Bra023104 | A03 |
|  | AT3G53430 | Bra017204 | A04 |
|  | AT3G53430 | Bra039782 | A04 |
|  | AT3G53430 | Bra039787 | A04 |
|  | AT3G53430 | Bra003134 | A07 |
|  | AT3G53430 | Bra006998 | A09 |
|  | AT5G60670 | Bra002460 | A10 |
| **L13** | AT3G49010 | Bra029931 | A01 |
|  | AT3G49010 | Bra018017 | A06 |
|  | AT3G49010 | Bra019574 | A06 |
|  | AT3G49010 | Bra026477 | A09 |
| **L13a** | AT3G24830 | Bra013239 | A03 |
|  | AT3G24830 | Bra015088 | A07 |
|  | AT3G24830 | Bra036669 | A09 |
|  | AT5G48760 | Bra020681 | A02 |
|  | AT5G48760 | Bra036161 | A09 |
| **L14** | AT2G20450 | Bra038852 | A07 |
|  | AT4G27090 | Bra026380 | A01 |
|  | AT4G27090 | Bra019070 | A03 |
|  | AT4G27090 | Bra010412 | A08 |
| **L15** | AT4G16720 | Bra038503 | A01 |
|  | AT4G16720 | Bra012667 | A03 |
|  | AT4G16720 | Bra012694 | A03 |
|  | AT4G16720 | Bra040747 | Scaffold000247 |
| **L17** | AT1G27400 | Bra010943 | A08 |
|  | AT1G27400 | Bra032828 | A09 |
|  | AT1G67430 | Bra033999 | A02 |
|  | AT1G67430 | Bra004225 | A07 |
| **L18** | AT3G05590 | Bra001156 | A03 |
|  | AT3G05590 | Bra039423 | A05 |
|  | AT3G05590 | Bra009940 | A06 |
|  | AT3G05590 | Bra040533 | Scaffold000215 |
|  | AT5G27850 | Bra036611 | A09 |
| **L18a** | AT1G29970 | Bra027325 | A05 |
|  | AT1G29970 | Bra032348 | A09 |
|  | AT2G34480 | Bra001559 | A03 |
|  | AT2G34480 | Bra022978 | A03 |
|  | AT2G34480 | Bra021915 | A04 |
|  | AT2G34480 | Bra005421 | A05 |
|  | AT2G34480 | Bra010805 | A08 |
| **L19** | AT1G02780 | Bra032586 | A09 |
|  | AT3G16780 | Bra000887 | A03 |
|  | AT3G16780 | Bra030509 | A08 |
|  | AT3G16780 | Bra036285 | A09 |
|  | AT3G16780 | Bra033346 | A10 |
| **L21** | AT1G09590 | Bra020000 | A06 |
|  | AT1G09590 | Bra020009 | A06 |
|  | AT1G57660 | Bra027910 | A09 |
| **L22** | AT3G05560 | Bra020575 | A02 |
|  | AT3G05560 | Bra001154 | A03 |
|  | AT3G05560 | Bra039428 | A05 |
|  | AT3G05560 | Bra009943 | A06 |
|  | AT3G05560 | Bra033351 | A10 |
|  | AT3G05560 | Bra040539 | Scaffold000215 |
| **L23** | AT1G04480 | Bra032506 | A09 |
|  | AT1G04480 | Bra015319 | A10 |
|  | AT3G04400 | Bra040138 | A01 |
|  | AT3G04400 | Bra030573 | A08 |
|  | AT3G04400 | Bra032388 | A09 |
| **L23a** | AT2G39460 | Bra000135 | A03 |
|  | AT2G39460 | Bra017070 | A04 |
|  | AT2G39460 | Bra005038 | A05 |
|  | AT3G55280 | Bra014758 | A04 |
|  | AT3G55280 | Bra014763 | A04 |
| **L24** | AT2G36620 | Bra005258 | A05 |
|  | AT3G53020 | Bra019632 | A04 |
| **L26** | AT3G49910 | Bra029984 | A01 |
|  | AT3G49910 | Bra012916 | A03 |
|  | AT3G49910 | Bra036038 | A09 |
| **L27** | AT4G15000 | Bra023843 | A01 |
|  | AT4G15000 | Bra036919 | A01 |
|  | AT4G15000 | Bra001857 | A03 |
|  | AT4G15000 | Bra031339 | A05 |
|  | AT4G15000 | Bra037080 | A05 |
|  | AT4G15000 | Bra039703 | A08 |
| **L27a** | AT1G23290 | Bra012370 | A07 |
|  | AT1G23290 | Bra016354 | A08 |
|  | AT1G23290 | Bra024577 | A09 |
|  | AT1G70600 | Bra007929 | A02 |
|  | AT1G70600 | Bra003951 | A07 |
| **L28** | AT2G19730 | Bra039014 | A07 |
|  | AT2G19730 | Bra031093 | A09 |
|  | AT2G19730 | Bra036710 | A09 |
|  | AT4G29410 | Bra011106 | A01 |
|  | AT4G29410 | Bra001548 | A03 |
|  | AT4G29410 | Bra024145 | A03 |
|  | AT4G29410 | Bra010329 | A08 |
| **L29** | AT3G06680 | Bra040286 | A01 |
|  | AT3G06680 | Bra001213 | A03 |
|  | AT3G06680 | Bra029600 | A05 |
| **L30** | AT1G36240 | Bra037566 | A01 |
|  | AT1G36240 | Bra022351 | A05 |
|  | AT1G77940 | Bra003662 | A07 |
|  | AT1G77940 | Bra003662 | A07 |
| **L31** | AT2G19740 | Bra031094 | A09 |
|  | AT2G19740 | Bra036709 | A09 |
|  | AT2G19740 | Bra038580 | Scaffold000149 |
|  | AT5G56710 | Bra026455 | A01 |
|  | AT5G56710 | Bra035629 | A02 |
|  | AT5G56710 | Bra002798 | A10 |
| **L32** | AT4G18100 | Bra013284 | A01 |
|  | AT4G18100 | Bra012616 | A03 |
|  | AT4G18100 | Bra021023 | A08 |
|  | AT4G18100 | Bra017545 | A09 |
| **L34** | AT1G26880 | Bra016295 | A08 |
|  | AT1G69620 | Bra007871 | A02 |
|  | AT1G69620 | Bra033066 | A02 |
|  | AT1G69620 | Bra003991 | A07 |
|  | AT1G69620 | Bra004400 | A07 |
|  | AT3G28900 | Bra039041 | A09 |
| **L35** | AT3G09500 | Bra034070 | A01 |
|  | AT3G09500 | Bra001306 | A03 |
|  | AT3G09500 | Bra029771 | A05 |
|  | AT2G39390 | Bra000131 | A03 |
|  | AT5G02610 | Bra028867 | A02 |
|  | AT5G02610 | Bra009577 | A10 |
| **L35a** | AT1G07070 | Bra008144 | A02 |
|  | AT1G07070 | Bra003808 | A07 |
|  | AT1G74270 | Bra007188 | A09 |
| **L36** | AT3G53740 | Bra000009 | A03 |
|  | AT3G53740 | Bra017170 | A04 |
|  | AT3G53740 | Bra005180 | A05 |
|  | AT3G53740 | Bra003155 | A07 |
| **L36a** | AT3G23390 | Bra001911 | A03 |
|  | AT4G14320 | Bra023728 | A01 |
|  | AT4G14320 | Bra033491 | A01 |
|  | AT4G14320 | Bra014945 | A07 |
|  | AT4G14320 | Bra010760 | A08 |
| **L37** | AT1G52300 | Bra001624 | A03 |
|  | AT1G52300 | Bra027192 | A05 |
|  | AT1G52300 | Bra030393 | A05 |
| **L37a** | AT3G60245 | Bra014503 | A04 |
|  | AT3G60245 | Bra003406 | A07 |
|  | AT3G60245 | Bra007519 | A09 |
| **L38** | AT2G43460 | Bra037714 | A04 |
|  | AT2G43460 | Bra003381 | A07 |
|  | AT3G59540 | Bra014535 | A04 |
| **L39** | AT3G02190 | Bra039161 | A05 |
|  | AT4G31985 | Bra023970 | A03 |
|  | AT4G31985 | Bra032033 | A04 |
|  | AT4G31985 | Bra040064 | A08 |
| **L40** | AT3G52590 | Bra033420 | A04 |
| **L41** | NONE |  |  |
