# Supplementary material for: Genome-specific differential gene expressions in resynthesized Brassica allotetraploids from pair-wise crosses of three cultivated diploids revealed by RNA-seq
Source: Front Plant Sci. 2015 Nov 4;6:957. doi: 10.3389/fpls.2015.00957 (PMC4631939; doi:10.3389/fpls.2015.00957)
Supplement: Supplementary Table 5 — The corresponding primers of qRT-PCR. [file Table5.DOCX]

**Supplementary Table 5.The corresponding primers of qRT-PCR**

| **Gene** | **Primer sequences (Left)** | **Primer sequences (Right)** |
| --- | --- | --- |
| Actin of B. napus | TCCATCCATCGTCCACAG | GCATCATCACAAGCATCCTT |
| Bra004584 | TAACGCTGCAACTGGAACTG | CCCATCACATAACCAGGGAC |
| Bra005702 | AAGTCCGCTCTCAAGGAACA | GGTTGATGTTTCCAGCGTCT |
| Bra008162 | CAATGTGGAGGGGAATGC | GTTTGTTGCCGTAAGTGCCT |
| Bra014966 | CCCTTCCCTTATCCAAGAGG | GTGAGCAGGTCTCAAGGAGG |
| Bra018521 | AATGGTGGTGCTGATGATGA | ATGTAGCCTGCGTTTGGAGT |
| Bra020022 | AAGTCATCTCGAGGCAGGAA | AGTCCTCTCGTGCAAATGCT |
| Bra024530 | AGACGAACGAGGAGAACCAA | CGGAGATTAGCGTAGCAAGG |
| Bra026915 | CCTTGACCGAACGCATAGAT | ACGCCAACAGAAACAACTCC |
| Bra027049 | GGATTGATGTTCCTCCACTCA | CTGAAACTTGACCCCAGCAT |
| Bra027133 | TTCCAACAGCCAAATACCTG | CTTCACCACCCTCTTTTTCG |
| Bra028228 | GGCTCAAAGGATTGTACGGA | TGCATCACATGGTGGAAGTT |
| Bra035862 | GGATGAGAAGCCTGAACTGG | CGGTAACAACCACAACCACA |
| Bra036614 | GGCCAATTCTCCAAAAGGTT | GGCTGCAATCACTCTTAGCC |
| Bra038175 | GTGGAGATGGCAGAAGAAGC | TATACATCCCGTCGTCCTCC |
| Bra038242 | CAGGGGACATAGGGAAAACA | TCGTCTCAGGCAACATCAAG |
| Bra038966 | AATGCCCTTTCTTGGTGATG | GGGTCTGCCTTTGTGTCAAT |
| Bra039182 | TCCTCGTCTTGTCAGAGAGC | TCCAACAATCTCAGCAGGGT |
